# Tetrahydrocurcumin Derivatives Enhanced the Anti-Inflammatory Activity of Curcumin: Synthesis, Biological Evaluation, and Structure–Activity Relationship Analysis

**DOI:** 10.3390/molecules28237787

**Published:** 2023-11-26

**Authors:** Yisett González, Randy Mojica-Flores, Dilan Moreno-Labrador, Marisín Pecchio, K. S. Jagannatha Rao, Maicol Ahumedo-Monterrosa, Patricia L. Fernández, Oleg V. Larionov, Johant Lakey-Beitia

**Affiliations:** 1 Center for Molecular and Cellular Biology of Diseases, Instituto de Investigaciones Científicas y Servicios de Alta Tecnología (INDICASAT AIP), Clayton, City of Knowledge, Panama City 0843-01103, Panama; ygonzalez@indicasat.org.pa (Y.G.); dmoreno@indicasat.org.pa (D.M.-L.); pllanes@indicasat.org.pa (P.L.F.); 2 Sistema Nacional de Investigación (SNI), SENACYT, Panama City 0816-02852, Panama; 3 Center for Biodiversity and Drug Discovery, Instituto de Investigaciones Científicas y Servicios de Alta Tecnología (INDICASAT AIP), Clayton, City of Knowledge, Panama City 0843-01103, Panama; rmojica@indicasat.org.pa; 4 Center for Academic Affairs and Collaboration, Instituto de Investigaciones Científicas y Servicios de Alta Tecnología (INDICASAT AIP), Clayton, City of Knowledge, Panama City 0843-01103, Panama; mpecchio@indicasat.org.pa; 5 Department of Biotechnology, Koneru Lakshmaiah Education Foundation (KLEF) Deemed to be University, Vaddeswaram 522 302, India; prochancellor@kluniversity.in; 6 Natural Products Group, School of Pharmaceutical Sciences, Zaragocilla Campus, University of Cartagena, Cartagena 130015, Colombia; mahumedom@unicartagena.edu.co; 7 Department of Chemistry, The University of Texas at San Antonio, San Antonio, TX 78249, USA

**Keywords:** curcumin, tetrahydrocurcumin, tetrahydrocurcumin derivatives, succinate, cytokine, TNF-α, IL-6, PGE_2_, anti-inflammatory activity, structure–activity relationship, 3D-QSAR

## Abstract

Tetrahydrocurcumin, the most abundant curcumin transformation product in biological systems, can potentially be a new alternative therapeutic agent with improved anti-inflammatory activity and higher bioavailability than curcumin. In this article, we describe the synthesis and evaluation of the anti-inflammatory activities of tetrahydrocurcumin derivatives. Eleven tetrahydrocurcumin derivatives were synthesized via Steglich esterification on both sides of the phenolic rings of tetrahydrocurcumin with the aim of improving the anti-inflammatory activity of this compound. We showed that tetrahydrocurcumin (**2**) inhibited TNF-α and IL-6 production but not PGE_2_ production. Three tetrahydrocurcumin derivatives inhibited TNF-α production, five inhibited IL-6 production, and three inhibited PGE_2_ production_._ The structure–activity relationship analysis suggested that two factors could contribute to the biological activities of these compounds: the presence or absence of planarity and their structural differences. Among the tetrahydrocurcumin derivatives, cyclic compound **13** was the most active in terms of TNF-α production, showing even better activity than tetrahydrocurcumin. Acyclic compound **11** was the most effective in terms of IL-6 production and retained the same effect as tetrahydrocurcumin. Moreover, acyclic compound **12** was the most active in terms of PGE_2_ production, displaying better inhibition than tetrahydrocurcumin. A 3D-QSAR analysis suggested that the anti-inflammatory activities of tetrahydrocurcumin derivatives could be increased by adding bulky groups at the ends of compounds **2**, **11**, and **12**.

## 1. Introduction

**Figure 1:**
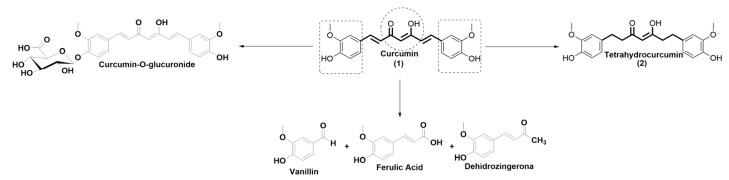
Curcumin and its metabolites.

Curcumin (**1**) is a natural polyphenol compound in the curcuminoid group that is found in the rhizome of *Curcuma longa* [1]. This molecule exhibits a wide spectrum of biological activities, such as antioxidant [2,3], anti-inflammatory [4], neuroprotective [5,6,7], antibacterial [8,9], and anticancer [3,10] activities. Curcumin (Figure 1) is composed of a diarylheptanoid with two O-methoxyphenols attached to a β-diketone moiety and two symmetrical olefinic bonds [11]. In this molecule, the phenolic rings and β-diketone moieties can be degraded in vitro and in vivo by oxidation and hydrolysis reactions [12,13,14]. At physiological pH, this pleiotropic molecule is transformed into vanillin, ferulic acid, dehydrozingerone, curcumin-O-glucuronide, and tetrahydrocurcumin [6,15] (Figure 1).

Tetrahydrocurcumin (**2**) has been isolated from Curcuma wenyujin [16]. This polyphenol is also the major curcumin metabolite in biological systems [6,17]. Tetrahydrocurcumin has similar biological activities to its precursor, including anti-inflammatory, antioxidant, and anticancer effects [10,17,18,19,20,21]. This polyphenolic compound provides renoprotection against several nephritic disorders by modulating inflammation and oxidative stress [19]. Previous research showed that this metabolite ameliorated the inflammatory response by decreasing the expression of the cytokines TNF-α and IL-6 in a mouse model of sepsis-induced acute kidney injury (AKI), and this effect was dependent on silent information regulator sirtuin 1 (SIRT1) signaling [19]. Tetrahydrocurcumin also inhibited the LPS-induced release of TNF-α and IL-6 by inhibiting IκB-α degradation [10]. In a mouse model of obesity-related inflammatory skin diseases, tetrahydrocurcumin reduced the levels of TNF-α and phosphorylated p65 in the animals’ skin [21].

Tetrahydrocurcumin could be a more favorable drug candidate than curcumin because it has multiple properties that curcumin does not have, such as being more stable under physiological conditions, more lipophilic, and more bioactive than curcumin. [17,18,22,23]. Tetrahydrocurcumin has the same reactive sites as curcumin (phenolic rings and β-diketone moieties), suggesting that it could also be degraded in biological systems under physiological conditions [23] (Figure 1). We hypothesized that structurally modifying tetrahydrocurcumin would improve the biological activity and increase the bioavailability of this compound. This approach could be an alternative to take advantage of the benefits of curcumin while eliminating its disadvantages. We focused on protecting the phenolic rings of tetrahydrocurcumin by incorporating a succinyl group into its structure [24,25]. Previous research has demonstrated that the succinylation of curcuminoids protects these molecules from hydrolysis [12]. Therefore, we added a succinyl group to the tetrahydrocurcumin structure to protect it from degradation [12,24,25].

In this study, we synthesized new tetrahydrocurcumin derivatives and performed a structure–activity relationship (SAR) analysis. A succinyl group was incorporated to protect the hydroxyl group, forming a diester on the aromatic ring of tetrahydrocurcumin. We evaluated the anti-inflammatory activities of the derivatives for comparison with that of tetrahydrocurcumin. A 3D-QSAR model including a group of compounds synthesized by our research group was developed, with the aim of improving the activity–structure relationship analysis and forecasting the activities of untested compounds.

## 2. Results

### 2.1. Synthesis of the Novel Tetrahydrocurcumin Derivatives

**Figure 2:**
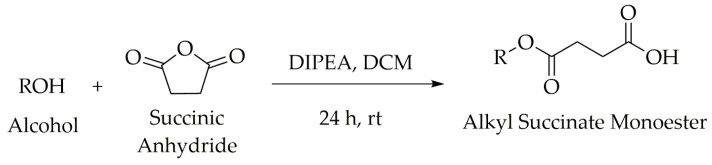
Methodology for synthesis of alkyl succinate monoesters (**S1**–**S11**).

The tetrahydrocurcumin derivatives were synthesized in two steps. First, each alkyl succinate derivative was synthesized from the alcohol of interest with succinic anhydride (Figure 2). In our recently published study [6], compounds **S1**, **S3**, and **S5**–**S9** (Figure S2) were synthesized on a large scale, and they were used here for coupling with tetrahydrocurcumin (**2**). Moreover, compounds **S2**, **S4**, and **S10**–**S11** were synthesized using our previously described methodology [5]. Compounds **S1**–**S11** were synthesized via succinylation reactions (Figure 2).

**Figure 3:**
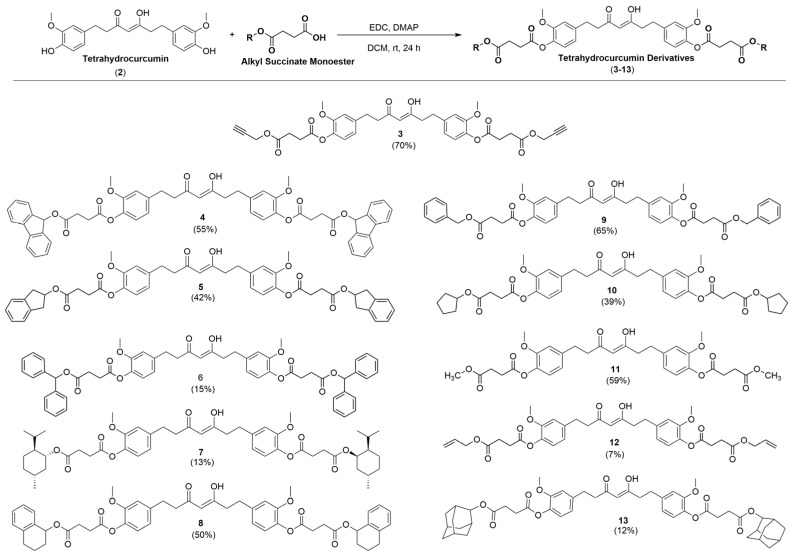
Synthesis of tetrahydrocurcumin derivatives (**3–13**).

In the second step, each alkyl succinate derivative (**S1**–**S11**) was coupled with tetrahydrocurcumin (**2**) to produce the corresponding tetrahydrocurcumin derivative (Figure 3). Compounds **3**–**13** were synthesized via Steglich esterification. Eleven novel tetrahydrocurcumin derivatives (**3**–**13**) were synthesized to determine their biological activities and establish the structure–activity relationship (SAR). The NMR signals of the protons of succinate are around 2.7 ppm and 2.9 ppm, while the carbon signals are around 28 ppm and 29 ppm. However, the signal proton of C-H of the tetrahydrocurcumin derivative is around 5.4 ppm, and the carbon signal is around 99 ppm, pointing to the presence of the *Z*-enol region, in line with previously observed values for other curcumin derivatives [26].

### 2.2. Anti-Inflammatory Activities of the Tetrahydrocurcumin Derivatives In Vitro

**Figure 4:**
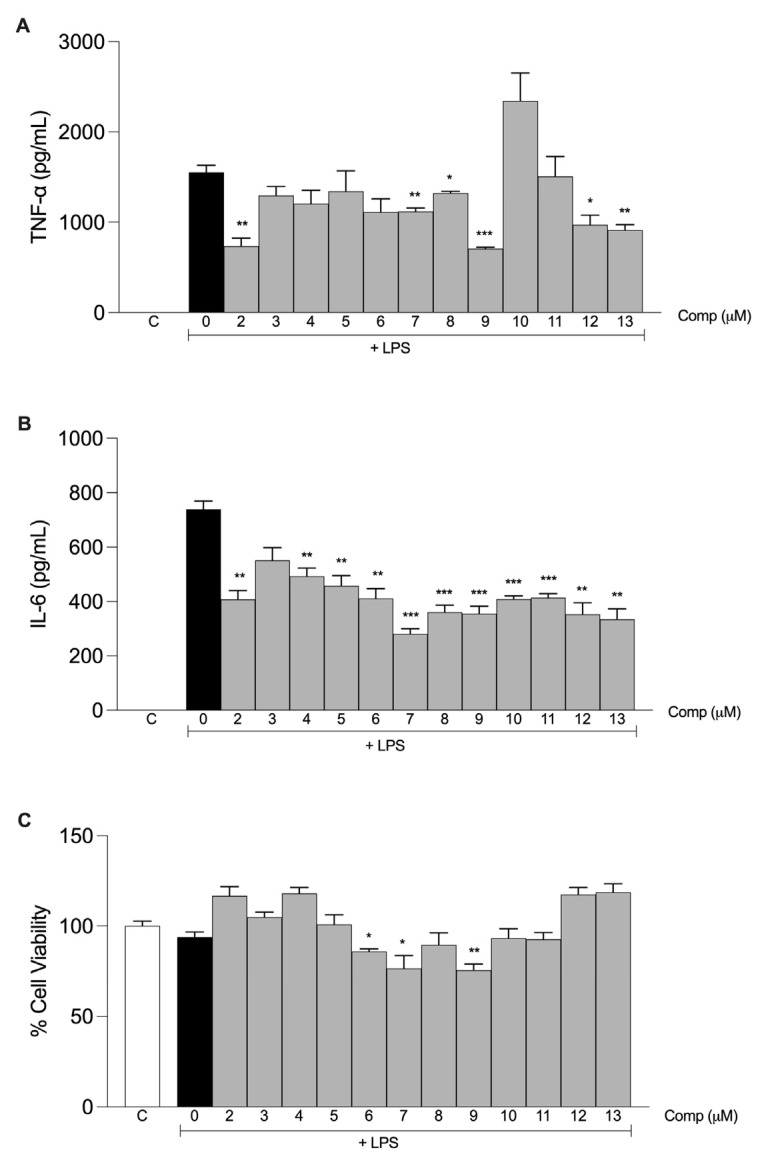
Anti-inflammatory activities of the tetrahydrocurcumin derivatives. Macrophages from the peritoneal cavities of C57BL/6 mice were treated with 30 μM of each compound. After 1 h, the cells were stimulated with 10 ng/mL LPS. The supernatants were harvested after 6 h of stimulus, and the levels of TNF-α (**A**) and IL-6 (**B**) were determined by ELISA. (**C**) After supernatant collection, cell viability was evaluated by using the MTT assay. The results are presented as the mean ± S.E.M. from two experiments performed in triplicate. *, *p* < 0.05; **, *p* < 0.01; ***, *p* < 0.001 compared to LPS stimulus alone (black bar). C, negative control.

The production of inflammatory mediators induced by LPS in macrophages treated with the compounds was measured to evaluate the anti-inflammatory activities of tetrahydrocurcumin and its derivatives. The effect of a single concentration (30 μM) of each compound on the secretion of TNF-α and IL-6 was evaluated in murine macrophages stimulated with LPS. Tetrahydrocurcumin (**2**) and derivatives **7**–**9** and **12**–**13** inhibited the production of TNF-α (Figure 4A). All compounds, except compound **3**, inhibited the production of IL-6 (Figure 4B). Tetrahydrocurcumin derivatives **6**, **7**, and **9** were cytotoxic (Figure 4C) and were excluded from further analysis. Tetrahydrocurcumin was used as a reference to evaluate which modifications had a greater influence on the anti-inflammatory activity.

**Figure 5:**
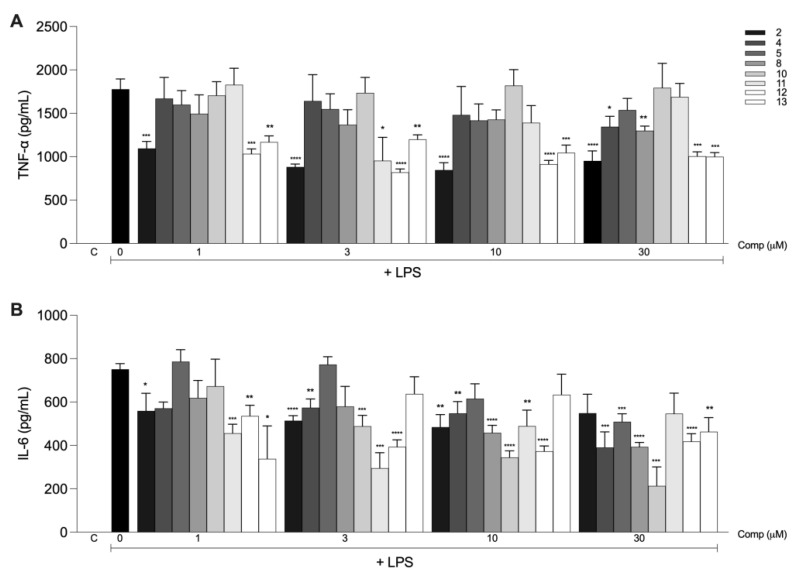
Tetrahydrocurcumin derivatives inhibit the secretion of inflammatory mediators induced by LPS in macrophages. Cells obtained from the peritoneal cavities of C57BL/6 mice were treated with different concentrations (1, 3, 10, or 30 μM) of different compounds and stimulated with LPS (10 ng/mL). Supernatants were harvested after 6 h of stimulus, and the levels of TNF-α (**A**) and IL-6 (**B**) were determined by ELISA. The results are presented as the mean ± S.E.M. from two experiments performed in triplicate. * *p* < 0.05; **, *p* < 0.01; ***, *p* < 0.001; ****, *p* < 0.0001 compared to LPS stimulus alone (black bar). C, negative control.

We then evaluated the effects of different concentrations of the selected compounds on TNF-α and IL-6 production (Figure 5). Tetrahydrocurcumin and derivatives **12**–**13** inhibited the production of TNF-α at all concentrations tested (Figure 5A). Moreover, compounds **4** and **8** inhibited TNF-α at the highest concentration (30 µM), while compound **11** showed an effect only when used at a concentration of 3 µM. Otherwise, compounds **4**, **5**, **8**, **10**, **12**, and **13** inhibited the production of IL-6 at 30 µM, whereas tetrahydrocurcumin and derivatives **4**, **8**, and **10**–**12** inhibited IL-6 at 10 µM (Figure 5B). At a concentration of 3 µM, tetrahydrocurcumin and derivatives **4** and **10**–**12** also exhibited this effect. At the lowest concentration (1 µM), only tetrahydrocurcumin and derivatives **11** and **12** inhibited the production of this cytokine.

**Figure 6:**
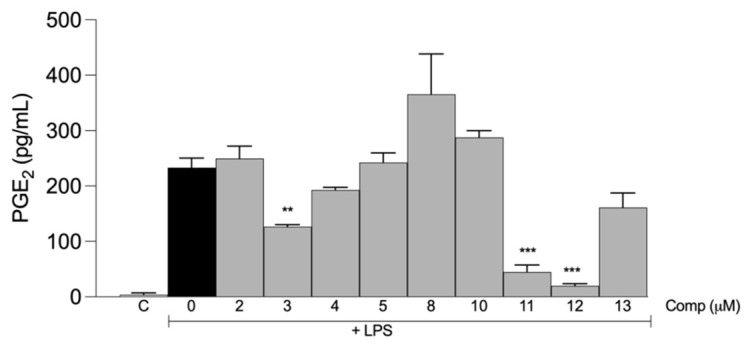
Tetrahydrocurcumin derivatives inhibit the production of PGE_2_ in macrophages stimulated with LPS. Macrophages from the peritoneal cavities of C57BL/6 mice were treated with 30 μM of each compound and then stimulated with 10 ng/mL LPS. Supernatants were collected after 6 h of stimulus, and levels of PGE_2_ were determined by ELISA. The results are presented as the mean ± S.E.M. from two experiments performed in triplicate. **, *p* < 0.01; ***, *p* < 0.001 compared to LPS stimulus alone (black bar). C, negative control.

PGE_2_ is a proinflammatory mediator produced by macrophages in response to LPS, and its synthesis is regulated by the enzyme cyclooxygenase (COX)-2 [27]. We evaluated the effect of tetrahydrocurcumin and its previously selected derivatives on the production of PGE_2_ (Figure 6). The levels of PGE_2_ were determined in the supernatants of macrophages stimulated with LPS with or without compound treatment (30 μM). Our results showed that compounds **3**, **11**, and **12** significantly reduced PGE_2_ production.

Table 1 shows the IC_50_ values of the compounds in terms of their effects on TNF-α (ranging from 0.18 ± 0.18 μM to 3.21 ± 4.52 μM) and IL-6 (ranging from 0.17 ± 0.20 μM to 9.13 ± 5.90 μM).

**Figure 7:**
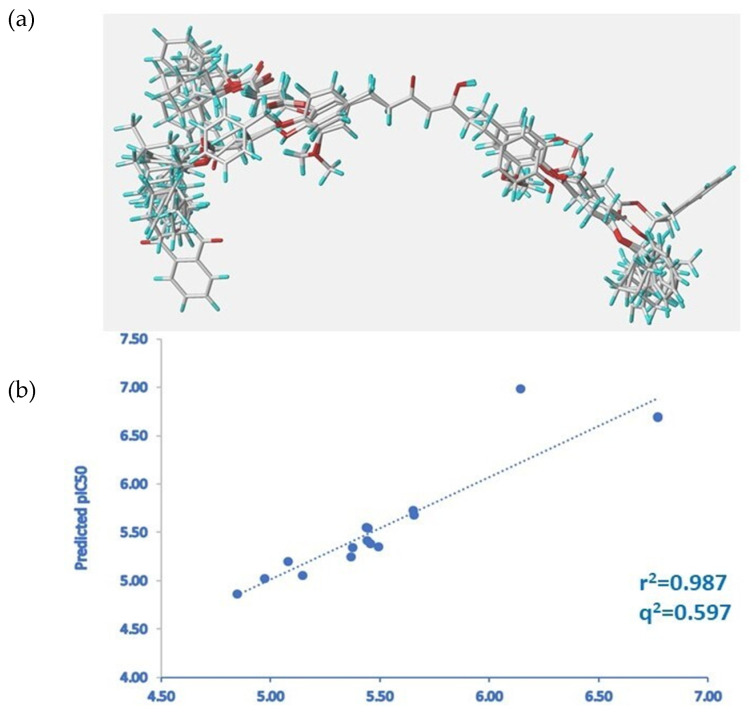
(**a**) Structural alignment used to obtain 3D-QSAR model. (**b**) Experimental pIC_50_ versus predicted pIC_50_ values for the 16 compounds in the training set.

Tetrahydrocurcumin was used as a template for the alignment of the compounds present in the database because it is one of the compounds with the best anti-inflammatory activity values. Figure 7a illustrates the alignment of all the compounds.

The results obtained from the PLS analysis are summarized in Table 2. For the CoMFA model, first with the model with STD, the leave-one-out cross-validated r^2^ value (q^2^) obtained was 0.424, and the non-cross-validated conventional r^2^ value was 0.973. Second, after applying region focusing (RF), the leave-one-out cross-validated r^2^ value (q^2^) obtained was 0.597, and the non-cross-validated conventional r^2^ value was 0.987. 

For the following explanations and discussions, the RF model will be taken as a reference. Figure 7b shows the linear scattering plots of experimental versus predicted activity for this model, with an r^2^ of 0.987 for the model, which is quite good, and a q^2^ of 0.597, with four being the optimal number of components. In both cases, the results indicate that the model is dominated by the steric component (65% for the SDT model and 57% for the RF model).

Table 3 and Table S2 in the supporting information show the experimental pIC_50_ values versus those predicted by the model and their respective residuals for the compounds in the training set and for the test set, respectively.

## 3. Discussion

Macrophages are cells of the innate immune system that are equipped with a variety of membrane receptors and intracellular machinery that can respond to harmful stimuli. These cells secrete a variety of mediators, including the cytokines TNF-α and IL-6 and prostaglandins, such as PGE_2_, when exposed to bacterial components. We evaluated the effect of tetrahydrocurcumin (**2**) and tetrahydrocurcumin derivatives (**3**–**13**) on the production of TNF-α, IL-6, and PGE_2_ by macrophages stimulated by the bacterial endotoxin LPS. Eleven tetrahydrocurcumin derivatives (**3**–**13**) were synthesized by first forming succinate and then esterifying tetrahydrocurcumin. The anti-inflammatory effects of the new tetrahydrocurcumin derivatives were analyzed to identify more active compounds with better structural stability in biological systems. Several tetrahydrocurcumin derivatives showed anti-inflammatory effects, with significant inhibition of TNF-α, IL-6, and PGE_2_ production.

Tetrahydrocurcumin was active in terms of both TNF-α and IL-6 production but not PGE_2_ production. The structural difference between curcumin and tetrahydrocurcumin is that the former has conjugated double bonds that give this molecule a planar structure, whereas the latter molecule is the hydrogenated form of curcumin, which has free rotation around these bonds. The structural difference between tetrahydrocurcumin and this new set of eleven derivatives is the incorporation of a succinyl group with different substituents (acyclic, cyclic, acyclic aromatic, and cyclic aromatic).

In terms of TNF-α production, tetrahydrocurcumin showed the best activity, with an IC_50_ value of 0.18 ± 0.18 μM, followed by compounds **8**, **12**, and **13**, with IC_50_ values of 3.21 ± 4.52 μM, 0.70 ± 0.10 μM, and 0.35 ± 0.047 μM, respectively (Table 1). Tetrahydrocurcumin and its difunctionalized derivatives (**8**, **12**, and **13**) were active in terms of TNF-α production. The difunctionalized derivatives are more attractive because both sides of the phenolic ring of tetrahydrocurcumin are protected. Although the activities of these new derivatives were lower than that of tetrahydrocurcumin, the protection they provide to both sides of the phenolic ring is relevant for increasing their bioavailability in biological systems. Compound **13**, with a bulky substituent, was the most active tetrahydrocurcumin derivative. However, including this same substituent in the difunctionalized curcumin derivative yielded a compound with no effect on TNF-α production [25].

Regarding IL-6 production, tetrahydrocurcumin and acyclic compound **11** showed the best activity, with IC_50_ values of 0.17 ± 0.20 μM and 0.17 ± 0.21 μM, respectively, followed by cyclic compound **12** and bulky compound **13**, with IC_50_ values of 0.72 ± 0.38 μM and 1.83 ± 2.55 μM, respectively (Table 1). Compounds **4**–**5**, **8**, and **10** showed moderate IC_50_ values (4.28 ± 4.88, 9.13 ± 5.90, 3.48 ± 4.39, and 3.66 ± 4.21 μM, respectively). The difunctionalized compounds showed activity in terms of IL-6 production.

In terms of PGE_2_ production, acyclic compounds **3**, **11**, and **12** showed the highest activity, followed by aromatic compound **4** and cyclic compound **13**. We found that compound **12** was more biologically active than tetrahydrocurcumin in terms of PGE_2_ production.

The biological activity of compounds **1**–**13** could be influenced by the presence or absence of planarity. The planarity and nonplanarity of curcumin and tetrahydrocurcumin confer different biological activities. In this research, tetrahydrocurcumin, a nonplanar molecule, and its difunctionalized derivatives (**3**–**13**) showed activity against TNF-α, IL-6, and PGE_2_ production. These difunctionalized derivatives have both sides of the phenolic ring of tetrahydrocurcumin protected. In contrast, curcumin, a planar molecule, and its monofunctionalized derivatives were active only in terms of IL-6 and PGE_2_ production [24,25]. Here, we explored different substituents: acyclic (**3**, **11**, and **12**), cyclic (**7**, **10**, and **13**), acyclic aromatic (**6** and **9**), and aromatic (**4**–**5** and **8**). In terms of TNF-α production, a cyclic substituent was the most active (**13**), followed by an acyclic compound (**12**) and a cyclic aromatic compound (**8**). In terms of IL-6 production, the most active derivatives were acyclic compounds **11** and **12** and cyclic compound **13**. Regarding PGE_2_ production, acyclic compound **12** was the most active, followed by the other acyclic compounds (**3** and **12**).

Further studies are necessary to evaluate the mechanism of action of the difunctionalized compounds and continue the search for protective groups (with or without succinyl moieties) that are more favorable for improving biological stability and preventing degradation.

The cross-validated q^2^ is an important criterion for measuring the internal predictive ability of the 3D-QSAR model. The metrics obtained in the 3D-QSAR model were as follows: the cross-validated q^2^ is 0.597 with an ONC value of 4, the non-cross-validated r^2^ is 0.987, the SEE value is 0.1077, and the F value is 204.088 (Table 2). The validation metrics for the generated model are within the accepted parameters q^2^ > 0.5 and r^2^ > 0.6, which indicates that the model has predictive potential [28]. The validity of the 3D-QSAR model is confirmed by the good predictions of the activity of the compounds belonging to the test set, with residues in six of the seven compounds less than unity (Table S2).

**Figure 8:**
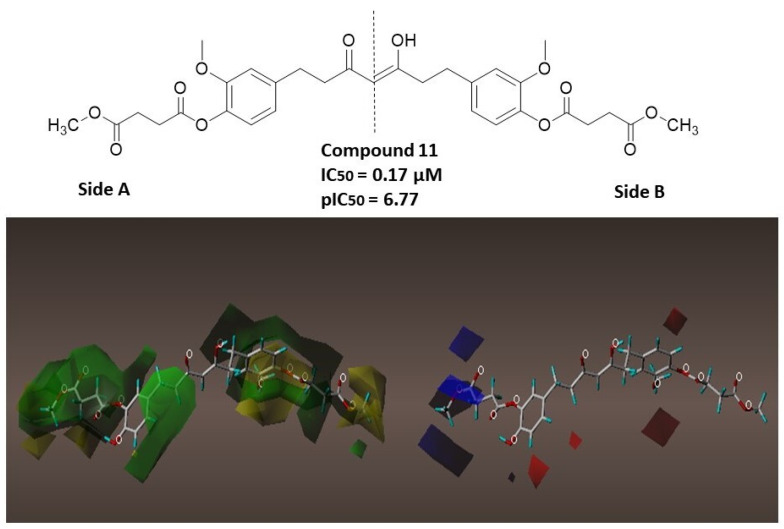
CoMFA contour maps are based on compound **11** as the template. Steric contour maps: green and yellow display the favorable and unfavorable regions. The red color means that the electronegative groups were beneficial for activity, while the blue color means that the electropositive groups were favorable.

The contributions of the steric and electrostatic fields were 57.3% and 42.7%, respectively; these results indicate that the model is slightly dominated by the steric component. The contour maps for compound **11** are shown in Figure 8 to explain the relationship between the structures and activities of the study compounds.

To better explain the generated contour maps, compound **11** will be divided into two sides, A and B, as shown at the top of Figure 8. Around the aromatic rings of compound **11** (pIC_50_ = 6.77) on both sides of the compound and the methyl of the methoxy group on the side, there is a green contour map indicating that the bulky substituents in this part of the compound lead to higher activity. A yellow contour map is observed around the oxygen atom of the methoxy groups present on both sides of compound **11**, indicating that the bulky substituents lead to a decrease in activity. The same behavior is observed in compounds **2** and **12** (Figures S4 and S5).

The blue contour near the position shows that the substitution of the electron-donating group will increase the activity of the compound. For compound **11**, a blue contour is observed in the region close to the hydrogens of the methoxy group on the A side of the compound, indicating that the increase in the positive charge of the substitute can help improve activity. The same behavior is observed in compound **1** (Figure S6). The red contours around regions indicate that the addition of an electronegative group can improve the activity. Small red regions can be observed near the hydroxyl groups present on both sides of the compound; therefore, increasing the electronegativity of the substituents in these areas could improve biological activity.

## 4. Materials and Methods

### 4.1. Synthesis

Chemical reagents were received from commercial sources (Tedia (Fairfield, OH, USA), Fischer (Ottawa, ON, Canada), AppliChem (Darmstadt, Germany), and Sigma Aldrich (St. Louis, MO, USA)). Curcumin was obtained commercially from Alfa Aesar with 95% total curcuminoid content from the turmeric rhizome. Tetrahydrocurcumin was obtained commercially from Sigma-Aldrich with 96% purity, as determined by HPLC. All reactions were conducted in borosilicate glass tubes (20 mL or 16 mL) fitted with screw caps and magnetically stirred under an argon atmosphere. Preparative thin-layer chromatography (prep-TLC) was carried out on hard-layer silica-gel-coated glass plates (Silicycle 60 F254 (MilliporeSigma, Burlington, MA, USA)). Plates were visualized under ultraviolet light (254 nm). The elution system was EtOAc/hexane (4:6, *v*/*v*).

^1^H and ^13^C NMR spectra were recorded at 500 MHz (^1^H) and 126 MHz (^13^C) on a Bruker AVANCE III 500 instrument in CDCl_3_, DMSO-d6, or other specified deuterated solvents with and without tetramethylsilane (TMS) as an internal standard at 25 °C unless specified otherwise. Chemical shifts (δ) are reported in parts per million (ppm) from tetramethylsilane (^1^H and ^13^C). Coupling constants (*J*) are given in Hz. Proton multiplicity is assigned using the following abbreviations: singlet (s), doublet (d), triplet (t), quartet (q), quintet (quint.), septet (sept.), and multiplet (m); additionally, the abbreviation for broad (br) is used. Infrared measurements were carried out neat on a Bruker Vector 22 FT-IR spectrometer fitted with a Specac diamond attenuated total reflectance (ATR) module. MS analyses were carried out on a Waters Xevo TQD spectrometer with an electrospray ionization (ESI) ion source (Waters Corporation, Milford, MA, USA). HRMS analyses were carried out on a maXis plus ESI-Q-TOF mass spectrometer (Bruker Daltonics, Billerica, MA, USA). The detailed synthetic procedures and spectral characterizations are described below.

### 4.2. General Procedure 1 (GP1) for the Synthesis of Alkyl Succinate Monoesters (***S1**–**S11***)

The alkyl succinate monoesters were prepared using our previously published methodology [24,25]. An oven-dried vial (20 mL) fitted with a screw cap and containing a magnetic stirrer was flushed with argon and charged with prop-2-yn-1-ol (104 μL, 3.44 mmol) and *N,N*-diisopropylethylamine (DIPEA) (466 μL, 2.68 mmol, 0.78 equiv.) and stirred in dichloromethane (DCM) (4 mL) at room temperature (RT). After 2 h, succinic anhydride (267.8 mg, 2.68 mmol, 0.78 equiv.) and 4-dimethylaminopyridine (DMAP) (326.9 mg, 2.68 mmol, 0.78 equiv.) were added, and the reaction was stirred at RT. After 48 h, the reaction mixture was diluted with brine/1 M HCl (3:1, 10 mL). The aqueous layer was extracted with DCM (3 × 20 mL), and the combined organic phases were dried over anhydrous sodium sulfate (Na_2_SO_4_) and concentrated under reduced pressure to obtain the desired product (Figure S1). 

In our recent publication [25], the following alkyl succinate monoesters (Figure S2) generated with this synthesis procedure were very well described, and because these compounds were produced on a large scale, they were used to bind to tetrahydrocurcumin: 4-((adamantan-2-yl)oxy)-4-oxobutanoic acid (**S1**), 4-(benzhydryloxy)-4-oxobutanoic acid (**S3**), 4-((9H-fluoren-9-yl)oxy)-4-oxobutanoic acid (**S5**), 4-((2,3-dihydro-1H-inden-2-yl)oxy)-4-oxobutanoic acid (**S6**), 4-(((1R,2S,5R)-2-isopropyl-5-methylcyclohexyl)oxy)-4-oxobutanoic acid (**S7**), 4-methoxy-4-oxobutanoic acid (**S8**), and 4-oxo-4-((1,2,3,4-tetrahydronaphthalen-1-yl)oxy)butanoic acid (**S9**).

#### 4.2.1. Synthesis of 4-oxo-4-(prop-2-yn-1-yloxy)butanoic Acid (**S2**)

According to GP1, prop-2-yn-1-ol (104 μL, 3.44 mmol) and DIPEA (466 μL, 2.68 mmol, 0.78 equiv.) were stirred in DCM (2 mL) at RT. After 2 h, succinic anhydride (267.8 mg, 2.68 mmol, 0.78 equiv.) and DMAP (326.9 mg, 2.68 mmol, 0.78 equiv.) were added, and the reaction mixture was stirred for 48 h at RT to yield monoester **S2** (339.3 mg, 63%). mp: 47.9 ± 0.2 °C. IR: 3286.1, 2954.3, 1711.9, 1419.5, 1206.6, 1152.1, 992.1 cm^−1^. MS (*m*/*z*) calcd. for C_7_H_8_NaO_4_: 179.13; found: 178.92 [M+Na^+^]. ^13^C NMR (126 MHz, DMSO): δ173.3, 171.6, 77.7, 51.8, 28.9, 28.6, 28.5 ppm.

#### 4.2.2. Synthesis of 4-(Benzyloxy)-4-oxobutanoic Acid (**S4**)

Phenylmethanol (481 µL, 500 mg, 4.62 mmol) and DIPEA (1200 μL, 6.94 mmol, 1.5 equiv.) were stirred in dichloromethane (DCM) (4 mL) at RT. After 2 h, succinic anhydride (694 mg, 6.93 mmol, 1.5 equiv.) and 4-dimethylaminopyridine (DMAP) (847.3 mg, 6.93 mmol, 1.50 equiv.) were added, and the reaction mixture was stirred for 48 h at RT to yield monoester **S4** (842.2 mg, 87%). IR: 2928.7, 1683.9, 1682.3, 1419.0, 1357.7, 1172.2, 731.5 cm^−1^. mp: 60.5 ± 0.2 °C. MS (*m*/*z*) calcd. for C_11_H_12_NaO_4_: 231.2; found: 231.9 [M + Na^+^].

#### 4.2.3. Synthesis of 4-(Cyclopentyloxy)-4-oxobutanoic Acid (**S10**)

Cyclopentanol (527 μL, 5.80 mmol) and DIPEA (1516 μL, 8.71 mmol, 1.5 equiv.) were stirred in DCM (4 mL) at RT. After 2 h, succinic anhydride (871.3 mg, 8.71 mmol, 1.5 equiv.) and DMAP (1063.8 mg, 8.71 mmol, 1.5 equiv.) were added, and the reaction mixture was stirred for 48 h at RT to yield monoester **S10** (704.5 mg, 65%). IR: 2962.9, 2875.5, 1716.9, 1227.6, 1156.6, 936.6 cm^−1^. mp: 37.3 ± 0.5 °C. MS (*m*/*z*) calcd. for C_9_H_14_NaO_4_: 209.2; found: 209.9 [M + Na^+^].

#### 4.2.4. Synthesis of 4-(Allyloxy)-4-oxobutanoic Acid (**S11**)

Prop-2-en-1-ol (585.5 μL, 8.61 mmol) and DIPEA (2249.2 μL, 12.9 mmol, 1.5 equiv.) were stirred in DCM (4 mL) at RT. After 2 h, succinic anhydride (1292.2 mg, 12.9 mmol, 1.5 equiv.) and DMAP (1577.6 mg, 12.9 mmol, 1.5 equiv.) were added, and the reaction mixture was stirred for 48 h at RT to yield monoester **S11** (677.1 mg, 50%). IR: 2989.2, 2929.8, 1699.1, 1680.3, 1418.4, 1304.9,1195.1, 906.6 cm^−1^. MS (*m*/*z*) calcd. for C_7_H_10_NaO_4_: 181.14; found: 180.92 [M + Na^+^].

### 4.3. General Procedure 2 (GP2) for the Synthesis of Tetrahydrocurcumin Derivatives

The tetrahydrocurcumin derivatives were prepared using a previously published methodology [5,6]. A borosilicate glass tube (16 mL) that was fitted with a screw cap and contained a magnetic stirrer was flushed with argon and charged with alkyl succinate (173.2 mg, 0.96 mmol, 1.8 equiv.), 4-dimethylaminopyridine (DMAP) (118.2 mg, 0.96 mmol, 1.8 equiv.), tetrahydrocurcumin (200 mg, 0.53 mmol), and dichloromethane (DCM) (3 mL); after combining, the reagents were stirred at 0 °C for 10 min. Then, N-(3-dimethylaminopropyl)-N′-ethylcarbodiimide hydrochloride (EDC) (185.4 mg, 0.96 mmol, 1.8 equiv.) was added, and the mixture was stirred at RT for 24 h. The reaction mixture was extracted with EtOAc and water (3 × 20 mL). The combined organic phases were concentrated under reduced pressure, and the remaining material was purified by HPLC to obtain the desired tetrahydrocurcumin derivative (Figure S3).

#### 4.3.1. Synthesis of (Z)-O,O′-((3-Hydroxy-5-oxohept-3-ene-1,7-diyl)bis(2-methoxy-4,1-phenylene)) di(prop-2-yn-1-yl) Disuccinate (**3**)

According to GP2, the reaction of succinate S2 (173.2 mg, 0.96 mmol, 1.8 equiv.), DMAP (118.2 mg, 0.96 mmol, 1.8 equiv.), tetrahydrocurcumin (200 mg, 0.53 mmol), and DCM (3 mL) was performed with stirring at 0 °C for 10 min. Then, EDC (185.4 mg, 0.96 mmol, 1.8 equiv.) was added, and the mixture was stirred at RT for 24 h. The crude product was purified by prep-TLC on silica gel (EtOAc/hexane, 4:6 *v*/*v*) to give compound **3** (243.7 mg, 70%). ^1^H NMR (500 MHz, CDCl_3_): 6.99–6.91 (m, 2H), 6.82–6.74 (m, 4H), 5.45 (s, 1H), 4.74 (d, *J* = 2.4 Hz, 4H), 3.87 (s, 2H), 3.81 (s, 6H), 2.98–2.89 (m, 8H), 2.82 (t, *J* = 6.9 Hz, 4H), 2.63–2.57 (m, 4H) ppm. ^13^C NMR (126 MHz, CDCl_3_): 193.0, 171.4, 170.5, 150.9, 139.8, 138.1, 122.7, 120.5, 112.7, 99.9, 77.6, 77.2, 75.2, 56.0, 52.4, 40.1, 31.5, 29.1, 28.9 ppm. IR: 3276.0, 2933.7, 1742.7, 1602.9, 1510.1, 1451.4, 1418.5, 1366.7, 1266.5, 1200.9, 1135.0 cm^−1^. HRMS (*m*/*z*): calcd. for C_35_H_36_O_12_ [M + Na^+^]^+^: 671.2099, found: 671.2084.

#### 4.3.2. Synthesis of (Z)-di(9H-Fluoren-9-yl)O,O′-((3-hydroxy-5-oxohept-3-ene-1,7-diyl)bis(2-methoxy-4,1-phenylene)) Disuccinate (**4**)

According to GP2, the reaction of succinate S5 (295.2 mg, 0.96 mmol, 1.8 equiv.), DMAP (118.2 mg, 0.96 mmol, 1.8 equiv.), tetrahydrocurcumin (200 mg, 0.53 mmol), and dichloromethane (3 mL) was performed with stirring at 0 °C for 10 min. Then, EDC (185.4 mg, 0.96 mmol, 1.8 equiv.) was added, and the mixture was stirred at RT for 24 h. The crude product was purified by prep-TLC on silica gel (EtOAc/hexane, 4:6 *v*/*v*) to give compound **4** (263.9 mg, 55%). ^1^H NMR (500 MHz, CDCl_3_): δ 7.67 (d, *J* = 7.6 Hz, 4H), 7.54 (d, *J* = 7.5 Hz, 4H), 7.41 (t, *J* = 7.5 Hz, 4H), 7.27 (t, *J* = 7.4 Hz, 4H), 6.90 (d, *J* = 7.9 Hz, 2H), 6.85 (s, 2H), 6.81–6.70 (m, 4H), 5.48–5.43 (m, 1H), 3.77 (s, 6H), 2.99 (t, *J* = 6.9 Hz, 4H), 2.93–2.85 (m, 8H), 2.63–2.54 (m, 4H) ppm. ^13^C NMR (126 MHz, CDCl_3_): δ 193.0, 172.9, 170.6, 150.9, 142.0, 141.1, 139.8, 138.1, 129.6, 128.0, 126.0, 122.8, 120.4, 120.1, 114.4, 112.7, 111.0, 99.9, 75.5, 55.9, 40.1, 31.5, 31.4, 29.7, 29.2 ppm. IR: 2934.5, 1758.5, 1731.6, 1602.2, 1509.6, 1451.5, 1417.9, 1361.5, 1239.1, 1200.2, 1132.3 cm^−1^. HRMS (*m*/*z*): calcd. for C_55_H_48_O_12_ [M + Na^+^]^+^: 923.3038, found: 923.3018.

#### 4.3.3. Synthesis of (Z)-bis(2,3-Dihydro-1H-inden-2-yl) O,O′-((3-hydroxy-5-oxohept-3-ene-1,7-diyl)bis(2-methoxy-4,1-phenylene)) Disuccinate (**5**)

According to GP2, the reaction of succinate S6 (248.5 mg, 0.96 mmol, 1.8 equiv.), DMAP (118.2 mg, 0.96 mmol, 1.8 equiv.), tetrahydrocurcumin (200 mg, 0.53 mmol), and DCM (3 mL) was performed with stirring at 0 °C for 10 min. Then, EDC (185.4 mg, 0.96 mmol, 1.8 equiv.) was added, and the mixture was stirred at RT for 24 h. The crude product was purified by prep-TLC on silica gel (EtOAc/hexane, 4:6 *v*/*v*) to give compound **5** (181.4 mg, 42%).^1^H NMR (500 MHz, CDCl_3_): δ 7.26–7.16 (m, 8H), 6.90 (d, *J* = 8.1 Hz, 2H), 6.80–6.69 (m, 4H), 5.62–5.54 (m, 2H), 5.45 (s, 1H), 3.78 (s, 6H), 3.33 (dd, *J* = 17.0, 6.5 Hz, 4H), 3.04 (d, *J* = 17.0 Hz, 4H), 2.93–2.86 (m, 8H), 2.70 (t, *J* = 7.0 Hz, 4H), 2.62–2.53 (m, 4H) ppm. ^13^C NMR (126 MHz, CDCl_3_): δ 193.0, 172.1, 170.7, 150.9, 140.4, 139.7, 138.1, 126.9, 124.7, 122.7, 122.7, 120.4, 120.4, 112.7, 99.9, 75.7, 55.9, 40.0, 39.6, 31.5, 31.4, 29.5, 29.0 ppm. IR: 2936.0, 1759.3, 1728.4, 1602.0, 1509.8, 1459.8, 1418.4, 1266.2, 1200.8, 1133.0 cm^−1^. HRMS (*m*/*z*): calcd. for C_47_H_48_O_12_ [M + Na^+^]^+^: 827.3038, found: 827.3024.

#### 4.3.4. Synthesis of (Z)-Dibenzhydryl O,O′-((3-hydroxy-5-oxohept-3-ene-1,7-diyl)bis(2-methoxy-4,1-phenylene)) Disuccinate (**6**)

According to GP2, the reaction of succinate S3 (297.2 mg, 0.96 mmol, 1.8 equiv.), DMAP (118.2 mg, 0.96 mmol, 1.8 equiv.), tetrahydrocurcumin (200 mg, 0.53 mmol), and DCM (3 mL) was performed with stirring at 0 °C for 10 min. Then, EDC (185.4 mg, 0.96 mmol, 1.8 equiv.) was added, and the mixture was stirred at RT for 24 h. The crude product was purified by prep-TLC on silica gel (EtOAc/hexane, 4:6 *v*/*v*) to give compound **6** (70.5 mg, 15%). ^1^H NMR (500 MHz, CDCl_3_): δ 7.43–7.25 (m, 20H), 6.95 (s, 2H), 6.87 (d, *J* = 8.1 Hz, 2H), 6.82–6.70 (m, 4H), 5.47 (s, 1H), 3.77 (s, 6H), 2.99–2.86 (m, 13H), 2.61 (t, *J* = 7.9 Hz, 4H) ppm. ^13^C NMR (126 MHz, CDCl_3_): δ 193.0, 171.1, 170.5, 150.9, 140.1, 139.7, 138.1, 128.7, 128.6, 128.6, 128.0, 127.2, 127.1, 122.8, 122.7, 120.4, 112.6, 99.9, 77.4, 77.4, 55.9, 40.1, 31.5, 29.7, 29.1 ppm. IR: 2934.8, 1734.5, 1602.1, 1509.9, 1453.2, 1418.1, 1362.5, 1266.9, 1201.1, 1134.2 cm^−1^. HRMS (*m*/*z*): calcd. for C_55_H_52_O_12_ [M + Na^+^]^+^: 927.3351, found: 927.3334.

#### 4.3.5. Synthesis of O,O′-(((Z)-3-Hydroxy-5-oxohept-3-ene-1,7-diyl)bis(2-methoxy-4,1-phenylene)) bis((1R,2S,5R)-2-Isopropyl-5-methylcyclohexyl) Disuccinate (**7**)

According to GP2, the reaction of succinate S7 (269.9 mg, 0.96 mmol, 1.8 equiv.), DMAP (118.2 mg, 0.96 mmol, 1.8 equiv.), tetrahydrocurcumin (200 mg, 0.53 mmol), and DCM (3 mL) was performed with stirring at 0 °C for 10 min. Then, EDC (185.4 mg, 0.96 mmol, 1.8 equiv.) was added, and the mixture was stirred at RT for 24 h. The crude product was purified by prep-TLC on silica gel (EtOAc/hexane, 4:6 *v*/*v*) to give compound **7** (57.4 mg, 13%). ^1^H NMR (500 MHz, CDCl_3_): δ 6.94 (d, *J* = 7.9 Hz, 2H), 6.80–6.69 (m, 4H), 5.44 (s, 1H), 4.76–4.67 (m, 2H), 3.79 (s, 6H), 2.90 (t, *J* = 7.9 Hz, 8H), 2.78–2.50 (m, 8H), 1.99 (d, *J* = 11.9 Hz, 2H), 1.91–1.82 (m, 2H), 1.72–1.22 (m, 10H), 1.09–0.93 (m, 4H), 0.88 (t, *J* = 7.0 Hz, 12H), 0.74 (d, *J* = 6.9 Hz, 6H) ppm. ^13^C NMR (126 MHz, CDCl_3_): δ 193.0, 171.7, 170.7, 151.0, 139.8, 138.2, 122.8, 122.8, 120.5, 112.7, 99.9, 77.4, 77.2, 76.9, 74.8, 56.0, 47.1, 41.0, 40.1, 34.4, 31.5, 31.5, 29.7, 29.2, 26.4, 23.6, 22.1, 20.9, 16.4 ppm. IR: 2953.8, 2869.5, 1763.3, 1729.2, 1604.1, 1510.9, 1454.5, 1418.8, 1367.9, 1137.6 cm^−1^. HRMS (*m*/*z*): calcd. for C_49_H_68_O_12_ [M + Na^+^]^+^: 871.4603, found: 871.4583.

#### 4.3.6. Synthesis of (Z)-O,O′-((3-Hydroxy-5-oxohept-3-ene-1,7-diyl)bis(2-methoxy-4,1-phenylene)) bis(1,2,3,4-Tetrahydronaphthalen-1-yl) Disuccinate (**8**)

According to GP2, the reaction of succinate S9 (262.3 mg, 0.96 mmol, 1.8 equiv.), DMAP (118.2 mg, 0.96 mmol, 1.8 equiv.), tetrahydrocurcumin (200 mg, 0.53 mmol), and DCM (3 mL) was performed with stirring at 0 °C for 10 min. Then, EDC (185.4 mg, 0.96 mmol, 1.8 equiv.) was added, and the mixture was stirred at RT for 24 h. The crude product was purified by prep-TLC on silica gel (EtOAc/hexane, 4:6 *v*/*v*) to give compound **8** (224.7 mg, 50%). ^1^H NMR (500 MHz, CDCl_3_): δ 7.30–7.07 (m, 8H), 6.88 (d, *J* = 8.0 Hz, 2H), 6.79–6.70 (m, 4H), 6.09–6.01 (m, 2H), 5.45 (s, 1H), 3.78 (s, 6H), 2.96–2.84 (m, 10H), 2.76 (t, *J* = 7.1 Hz, 6H), 2.59 (t, *J* = 7.9 Hz, 4H), 2.04–1.92 (m, 6H), 1.88–1.76 (m, 2H) ppm. ^13^C NMR (126 MHz, CDCl_3_): δ 193.0, 171.8, 170.7, 151.0, 139.8, 138.1, 138.1, 134.5, 129.6, 129.2, 128.3, 126.2, 122.8, 122.8, 120.5, 112.7, 99.9, 70.6, 56.0, 40.1, 31.5, 29.9, 29.2, 29.1, 18.9 ppm. IR: 2935.6, 1760.0, 1727.7, 1603.4, 1510.4, 1453.7, 1418.5, 1266.7, 1201.7, 1135.5 cm^−1^. HRMS (*m*/*z*): calcd. for C_49_H_52_O_12_ [M + Na]^+^: 855.3351, found: 855.3337.

#### 4.3.7. Synthesis of (Z)-Dibenzyl O,O′-((3-Hydroxy-5-oxohept-3-ene-1,7-diyl)bis(2-methoxy-4,1-phenylene)) Disuccinate (**9**)

According to GP2, the reaction of succinate S4 (201.3 mg, 0.96 mmol, 1.8 equiv.), DMAP (118.2 mg, 0.96 mmol, 1.8 equiv.), tetrahydrocurcumin (200 mg, 0.53 mmol), and DCM (3 mL) was performed with stirring at 0 °C for 10 min. Then, EDC (185.4 mg, 0.96 mmol, 1.8 equiv.) was added, and the mixture was stirred at RT for 24 h. The crude product was purified by prep-TLC on silica gel (EtOAc/hexane, 4:6 *v*/*v*) to give compound **9** (261.1 mg, 65%). ^1^H NMR (500 MHz, CDCl_3_): δ 7.36 (s, 9H), 6.90 (d, *J* = 8.0 Hz, 2H), 6.80–6.69 (m, 4H), 5.44 (s, 1H), 5.16 (s, 4H), 3.77 (s, 6H), 2.98–2.85 (m, 8H), 2.82–2.79 (m, 4H), 2.62–2.54 (m, 4H) ppm. ^13^C NMR (126 MHz, CDCl_3_): δ 193.0, 172.0, 170.6, 150.9, 139.8, 138.1, 135.9, 128.7, 128.4, 128.3, 122.7, 120.4, 114.4, 112.7, 111.0, 99.9, 77.2, 66.7, 55.9, 40.1, 31.5, 29.4, 29.1 ppm. IR: 2935.8, 1759.2, 1732.5, 1603.2, 1511.0, 1454.9, 1418.5, 1267.3, 1201.6, 1135.7 cm^−1^. HRMS (*m*/*z*): calcd. for C_43_H_44_O_12_ [M + Na^+^]^+^: 775.2725, found: 775.2710.

#### 4.3.8. Synthesis of (Z)-Dicyclopentyl O,O′-((3-Hydroxy-5-oxohept-3-ene-1,7-diyl)bis(2-methoxy-4,1-phenylene)) Disuccinate (**10**)

According to GP2, the reaction of succinate S10 (180.0 mg, 0.96 mmol, 1.8 equiv.), DMAP (118.2 mg, 0.96 mmol, 1.8 equiv.), tetrahydrocurcumin (200 mg, 0.53 mmol), and DCM (3 mL) was performed with stirring at 0 °C for 10 min. Then, EDC (185.4 mg, 0.96 mmol, 1.8 equiv.) was added, and the mixture was stirred at RT for 24 h. The crude product was purified by prep-TLC on silica gel (EtOAc/hexane, 4:6 *v*/*v*) to give compound **10** (147.7 mg, 39%). ^1^H NMR (500 MHz, CDCl_3_): δ 6.93 (d, *J* = 7.9 Hz, 2H), 6.79–6.69 (m, 4H), 5.43 (s, 1H), 5.25–5.13 (m, 2H), 3.78 (s, 6H), 2.93–2.82 (m, 8H), 2.68 (t, *J* = 7.0 Hz, 4H), 2.57 (t, *J* = 7.9 Hz, 4H), 1.90–1.78 (m, 4H), 1.75–1.63 (m, 8H), 1.64–1.51 (m, 4H) ppm. ^13^C NMR (126 MHz, CDCl_3_): δ 193.0, 171.9, 170.7, 150.9, 139.7, 138.1, 122.7, 120.4, 112.7, 99.9, 77.6, 55.9, 40.0, 32.7, 31.5, 29.6, 29.1, 23.8 ppm. IR: 2959.2, 1760.7, 1730.0, 1603.9, 1510.8, 1418.7, 1267.3, 1202.2, 1136.7 cm^−1^. HRMS (*m*/*z*): calcd. for C_39_H_48_O_12_ [M + Na^+^]^+^: 731.3038, found: 731.3033.

#### 4.3.9. Synthesis of (Z)-O,O′-((3-Hydroxy-5-oxohept-3-ene-1,7-diyl)bis(2-methoxy-4,1-phenylene)) dimethyl Disuccinate (**11**)

According to GP2, the reaction of succinate S8 (149.8 mg, 0.96 mmol, 1.8 equiv.), DMAP (118.2 mg, 0.96 mmol, 1.8 equiv.), tetrahydrocurcumin (200 mg, 0.53 mmol), and DCM (3 mL) was performed with stirring at 0 °C for 10 min. Then, EDC (185.4 mg, 0.96 mmol, 1.8 equiv.) was added, and the mixture was stirred at RT for 24 h. The crude product was purified by prep-TLC on silica gel (EtOAc/hexane, 4:6 *v*/*v*) to give compound **11** (189.6 mg, 59%). ^1^H NMR (500 MHz, CDCl_3_) δ 6.94 (d, *J* = 8.0 Hz, 2H), 6.79–6.69 (m, 4H), 5.42 (s, 1H), 3.78 (s, 6H), 3.71 (s, 6H), 2.94–2.84 (m, 8H), 2.74 (t, *J* = 7.0 Hz, 4H), 2.60–2.52 (m, 4H) ppm. ^13^C NMR (126 MHz, CDCl_3_): δ 193.0, 172.6, 170.7, 150.9, 139.8, 138.1, 122.7, 122.7, 120.4, 120.4, 112.7, 99.9, 77.4, 77.2, 76.9, 55.9, 52.0, 40.1, 31.5, 29.1, 29.1 ppm. IR: 2950.2, 1758.7, 1733.1, 1602.6, 1510.26, 1418.4, 1267.2, 1201.0, 1135.4, 1033.8 cm^−1^. HRMS (*m*/*z*): calcd. for C_31_H_36_O_12_ [M + Na^+^]^+^: 623.2099, found: 623.2095.

#### 4.3.10. Synthesis of (Z)-Diallyl O,O′-((3-hydroxy-5-oxohept-3-ene-1,7-diyl)bis(2-methoxy-4,1-phenylene)) Disuccinate (**12**)

According to GP2, the reaction of succinate S11 (152.9 mg, 0.96 mmol, 1.8 equiv.), 4-dimethylaminopyridine (118.2 mg, 0.96 mmol, 1.8 equiv.), tetrahydrocurcumin (200 mg, 0.53 mmol), and dichloromethane (3 mL) was performed with stirring at 0 °C for 10 min. Then, N-(3-dimethylaminopropyl)-N′-ethylcarbodiimide hydrochloride (185.4 mg, 0.96 mmol, 1.8 equiv.) was added, and the mixture was stirred at RT for 24 h. The crude product was purified by prep-TLC on silica gel (EtOAc/hexane, 4:6 *v*/*v*) to give compound **12** (23.9 mg, 7%). ^1^H NMR (500 MHz, CDCl_3_): 6.94 (d, *J* = 7.9 Hz, 2H), 6.79–6.67 (m, 4H), 5.97–5.86 (m, 2H), 5.43 (s, 1H), 5.32 (d, *J* = 17.1 Hz, 2H), 5.24 (d, *J* = 8.8 Hz, 2H), 4.62 (d, *J* = 5.7 Hz, 4H), 3.79 (s, 6H), 2.95–2.85 (m, 8H), 2.77 (t, *J* = 7.0 Hz, 4H), 2.57 (t, *J* = 7.8 Hz, 4H) ppm. ^13^C NMR (126 MHz, CDCl_3_) δ 193.0, 171.8, 170.7, 151.0, 139.8, 138.1, 132.1, 122.8, 122.8, 120.5, 120.4, 118.5, 112.7, 99.9, 65.6, 56.0, 40.1, 31.5, 31.5, 29.3, 29.1 ppm. IR: 2935.5, 1760.2, 1735.6, 1604.2, 1511.5, 1453.1, 1419.1, 1363.0, 1273.8, 1138.0 cm^−1^. HRMS (*m*/*z*): calcd. for C_35_H_40_O_12_ [M + Na^+^]^+^: 675.2412, found: 675.2406.

#### 4.3.11. Synthesis of di((1r,3r,5r,7r)-Adamantan-2-yl) O,O′-(((Z)-3-hydroxy-5-oxohept-3-ene-1,7-diyl)bis(2-methoxy-4,1-phenylene)) Disuccinate (**13**)

According to GP2, the reaction of succinate S1 (244.1 mg, 0.96 mmol, 1.8 equiv.), DMAP (118.2 mg, 0.96 mmol, 1.8 equiv.), tetrahydrocurcumin (200 mg, 0.53 mmol), and DCM (3 mL) was performed with stirring at 0 °C for 10 min. Then, EDC (185.4 mg, 0.96 mmol, 1.8 equiv.) was added, and the mixture was stirred at RT for 24 h. The crude product was purified by prep-TLC on silica gel (EtOAc/hexane, 4:6 *v*/*v*) to give compound **13** (52.7 mg, 12%). ^1^H NMR (500 MHz, CDCl_3_): δ 6.93 (d, *J* = 8.0 Hz, 2H), 6.79–6.69 (m, 4H), 5.43 (s, 1H), 4.96 (s, 2H), 3.79 (s, 6H), 2.96–2.83 (m, 8H), 2.77 (t, *J* = 7.0 Hz, 4H), 2.57 (t, *J* = 7.7 Hz, 3H), 2.06–1.96 (m, 8H), 1.87–1.71 (m, 16H), 1.58–1.51 (m, 4H) ppm. ^13^C NMR (126 MHz, CDCl_3_): δ 193.0, 171.4, 170.8, 151.0, 139.7, 138.1, 122.8, 122.7, 120.4, 112.7, 99.9, 77.6, 55.9, 40.1, 37.5, 36.4, 31.9, 31.9, 31.5, 29.9, 29.2, 27.3, 27.1 ppm. IR: 2906.6, 2854.6, 1762.4, 1728.8, 1603.6, 1510.2, 1451.2, 1360.1, 1266.8, 1201.8, 1136.8 cm^−1^. HRMS (*m*/*z*): calcd. for C_49_H_60_O_12_ [M + Na^+^]^+^: 863.3977, found: 863.3967.

### 4.4. Mice

In vitro assays with macrophage culture were carried out by using C57Bl/6 mice obtained from the mouse facility of the Instituto de Investigaciones Científicas y Servicios de Alta Tecnología (INDICASAT). Mice aged 8 weeks were kept at 25 °C under a 12 h light/dark cycle with free access to food and water. All experiments were performed in accordance with guidelines from the Institutional Animal Welfare Committee and the Guide for the Care and Use of Laboratory Animals of the National Institutes of Health. The protocol was also approved by the Institutional Animal Care and Use Committee of INDICASAT AIP (CICUA-18-007).

### 4.5. Macrophage Culture and Mediator Measurement

Peritoneal macrophages were obtained by washing the peritoneal cavities of C57B1/6 mice five days after intraperitoneal instillation of 2 mL of 3% thioglycollate. Macrophages were seeded in RPMI containing 10% FCS at 2 × 10^5^ cells/well in 96-well plates and cultured for 2 h at 37 °C under a 5% CO_2_ atmosphere. Adherent cells were pretreated with the compounds (30 μM) 1 h before stimulation with LPS (10 ng/mL). For dose–response experiments, compounds were used at concentrations of 1, 3, 10, and 30 μM. Supernatants were collected 6 h after LPS stimulation. Control and experimental treatments were performed in the presence of 0.5% DMSO, as the compounds were solubilized in this solvent. The concentrations of TNF-α, IL-6, and PGE_2_ were determined by ELISA (R&D System) following the manufacturer’s protocol.

### 4.6. Cytotoxicity Assay

After removing the supernatants, 100 μL of 0.5 mg/mL MTT in RPMI was added to each well. Cells were incubated overnight at 37 °C, and formazan crystals were dissolved in 100 µL of 0.04 M HCl in isopropanol. Then, the absorbance was measured at 570 nm using an ELISA plate reader. Cell viability was calculated using the following formula: % viability: [(OD sample) × 100%]/(OD control). Nonstimulated cells represented 100% viability.

### 4.7. Statistical Analysis

Results from the cytokine measurements and cytotoxicity assays were analyzed using the statistical software package GraphPad Prism 9.4.0. All data are presented as the mean ± S.E.M. Statistical analysis was performed by Student’s t-test. The differences between samples were considered significant when *p* < 0.05. The 50% inhibitory concentration (IC_50_) was calculated by adjusting a sigmoidal dose–response curve following the standard procedure in GraphPad Prism 5.

### 4.8. Three-Dimensional QSAR Model

Twenty-three compounds (Figure 3 and Figure 7) synthesized by our research group with their respective biological activities, reported as IC_50_ (Table S1), were used for the development of the 3D-QSAR model. Nine of these compounds are presented in the current work, and fourteen have been reported in previous works [24,25]. The 3D structures of all compounds were constructed by using the Gaussview program [29], and their geometry was optimized by using the density functional theory (DFT) with the B3LYP functional [30] and the 6-31G basis set by using the Gaussian program [31]. 

The optimized structures of the 23 compounds were aligned using compound 2 as a template in the Sybyl X (version 2.1.1, Tripos Inc. (Certara, L.P. St. Louis, MO, USA. 2014) program. The total compounds were divided into two sets: the training set consisting of 16 compounds (70% of total compounds) and the test set containing 7 compounds (30% of total compounds) for validating the reliability of the model. In the 3D-QSAR study, the anti-inflammatory activities of the studied compounds were expressed as IC_50_ values and converted into pIC_50_ values. The comparative molecular field analysis (CoMFA) descriptor was obtained by using the QSAR tool implemented in Sybyl X 2.1.1 [32]. The structures of derivatives used in the training and test sets and their anti-inflammatory activities (IC_50_ values) are shown in Table S1.

A partial least-squares (PLS) approach [33] was used to derive the 3D-QSAR models, in which the CoMFA descriptors were used as independent variables, and the experimental pIC_50_ values were used as dependent variables. Cross-validation with the leave-one-out (LOO) option in the SAMPLS program [34] was applied to obtain the optimal number of components to be used in the final analysis. The q^2^ (cross-validated r^2^), r^2^ (non-cross-validated r^2^), Spress (cross-validated standard error of prediction), and F values were computed.

## 5. Conclusions

In this research, we synthesized 11 new tetrahydrocurcumin derivatives and determined their effects on the production of the inflammatory mediators TNF-α, IL-6, and PGE_2_. A common feature among these derivatives was that a succinyl group was attached to tetrahydrocurcumin via a Steglich esterification reaction. The alkoxide group coupled to the connector moiety varied and was composed of acyclic, cyclic, acyclic aromatic, or cyclic aromatic groups. We added a succinyl group to the tetrahydrocurcumin structure to protect the new derivatives from degradation and determine how this group affects the anti-inflammatory response. These difunctionalized tetrahydrocurcumin derivatives showed biological activity against TNF-α, IL-6, and PGE_2_ production. An analysis of the contour maps suggests that the anti-inflammatory activity of the newly derived compounds could be increased by adding bulky groups at the ends of compounds **2**, **11**, and **12**. These compounds thus provide key components to build future anti-inflammatory molecules.

## Figures and Tables

**Table 1:** Anti-inflammatory activities of the novel tetrahydrocurcumin derivatives.

Compound	IC_50_ ± S.D. (μM) TNF-α	IC_50_ ± S.D. (μM) IL-6
**2**	0.18 ± 0.18	0.17 ± 0.20
**4**	N.A.	4.28 ± 4.88
**5**	N.A.	9.13 ± 5.90
**8**	3.21 ± 4.52	3.48 ± 4.39
**10**	N.A.	3.66 ± 4.21
**11**	N.A.	0.17 ± 0.21
**12**	0.70 ± 0.10	0.72 ± 0.38
**13**	0.35 ± 0.047	1.83 ± 2.55

Values represent the average IC_50_ values from two independent experiments performed in triplicate ± S.D.

**Table 2:** Statistical data for CoMFA models.

Model	q^2^ Cross-Validated r^2^	r^2^ Non-Cross-Validated r^2^	SE Standard Error	ONC NUMBER of Optimal Components	F Values	%E Contribution of Electrostatic Field	%S Contribution of Steric Field
STD standard scaling	0.424	0.973	0.108	4	97.620	35	65
RF region focusing	0.597	0.987	0.1077	4	204.088	42.7	0.573

**Table 3:** Experimental and predicted activities (pIC_50_) of the training set compounds and residual values.

Compound	Experimental pIC_50_	Predicted pIC_50_	Residues
**2**	6.77	6.69	0.08
**4**	5.37	5.25	0.12
**8**	5.46	5.38	0.07
**10**	5.44	5.55	−0.12
**11**	6.77	6.69	0.08
**12**	6.14	6.98	−0.84
**14**	5.65	5.72	−0.07
**15**	5.44	5.54	−0.10
**16**	5.66	5.68	−0.03
**18**	4.85	4.87	−0.02
**19**	5.08	5.20	−0.12
**20**	5.49	5.35	0.14
**21**	4.97	5.02	−0.05
**24**	5.44	5.41	0.03
**26**	5.38	5.35	0.03
**27**	5.15	5.06	0.09

## Data Availability

Data are contained within the article and Supplementary Material.

## Supplementary Materials

The following supporting information can be downloaded at https://www.mdpi.com/article/10.3390/molecules28237787/s1. General procedure 1 (GP1) for the synthesis of alkyl succinate monoesters, general procedure (GP2) for the synthesis of tetrahydrocurcumin derivatives (**3**–**13**).
