# Supplementary material for: Tetrahydrocurcumin Derivatives Enhanced the Anti-Inflammatory Activity of Curcumin: Synthesis, Biological Evaluation, and Structure–Activity Relationship Analysis
Source: Molecules. 2023 Nov 26;28(23):7787. doi: 10.3390/molecules28237787 (PMC10708537; doi:10.3390/molecules28237787)

Tetrahydrocurcumin Derivatives Enhanced the Anti-Inflammatory Activity of Curcumin: Synthesis, Biological Evaluation and Structure-Activity Relationship Analysis

Yisett González [1,2], Randy Mojica-Flores [3], Dilan Moreno-Labrador [1], Marisín Pecchio [4], K.S. Jagannatha Rao [5], Maicol Ahumedo-Monterrosa [6], Patricia L. Fernández [1,2], Oleg V. Larionov [7,*] and Johant Lakey-Beitia [2,3,7,*].

[1] Center for Molecular and Cellular Biology of Diseases, Instituto de Investigaciones Científicas y Servicios de Alta Tecnología (INDICASAT AIP), Clayton, City of Knowledge, Panama City 0843-01103, Panama; ygonzalez@indicasat.org.pa (Y.G.); dmoreno@indicasat.org.pa (D.M.-L.); pllanes@indicasat.org.pa (P.L.F.)

[2] Sistema Nacional de Investigación (SNI), SENACYT, Panama City 0816-02852, Panama; ygonzalez@indicasat.org.pa, pllanes@indicasat.org.pa, jlakey@indicasat.org.pa.

[3] Center for Biodiversity and Drug Discovery, Instituto de Investigaciones Científicas y Servicios de Alta Tecnología (INDICASAT AIP), Clayton, City of Knowledge, Panama City 0843-01103, Panama; jlakey@indicasat.org.pa (J.L-B.); rmojica@indicasat.org.pa (R.M-F)

[4] Center for Academic Affairs and Collaboration, Instituto de Investigaciones Científicas y Servicios de Alta Tecnología (INDICASAT AIP), Clayton, City of Knowledge, Panama City 0843-01103, Panama; mpecchio@indicasat.org.pa (M.P.)

[5] Department of Biotechnology, Koneru Lakshmaiah Education Foundation (KLEF) Deemed to be University, Vaddeswaram 522 302, India; prochancellor@kluniversity.in (K.S.R)

[6] Natural Products Group, School of Pharmaceutical Sciences, Zaragocilla Campus, University of Cartagena, Cartagena 130015, Colombia; mahumedom@unicartagena.edu.co

[7] Department of Chemistry, The University of Texas at San Antonio, San Antonio, TX 78249, USA; oleg.larionov@utsa.edu (O.V.L.); jlakey@indicasat.org.pa (J.L-B.).

* Correspondence: oleg.larionov@utsa.edu (O.V.L.); jlakey@indicasat.org.pa (J.L-B.); Tel.: +1-210-458-6050 (O.V.L.); +507-517-0700 (J.L.-B.)

General Procedures

Materials: Chemical reagents were received from commercial sources (Tedia, Fischer, AppliChem, and Sigma Aldrich). Curcumin was obtained commercially from Alfa Aesar with 95% total curcuminoid content from turmeric rhizome. Tetrahydrocurcumin was obtained commercially from Sigma–Aldrich with 96% purity by HPLC.

Experimental equipment: All reactions were conducted in borosilicate glass tubes (20 mL or 16 mL) fitted with a screw cap and magnetically stirred under an argon atmosphere.

Purification: Preparative thin-layer chromatography (prep-TLC) was carried out on hard-layer silica gel-coated glass plates (Silicycle 60 F254). Plates were visualized under ultraviolet light (254 nm). The elution system was EtOAc/hexane (4:6, v/v).

Characterization: ^{1}H and ^{13}C NMR spectra were recorded at 500 MHz (^{1}H) and 126 MHz (^{13}C) on Bruker AVANCE III 500 instruments in $CDCl_3$, DMSO-d6 or other specified deuterated solvents with and without tetramethylsilane (TMS) as an internal standard at 25 °C unless specified otherwise. Chemical shifts (δ) are reported in parts per million (ppm) from tetramethylsilane (^{1}H and ^{13}C). Coupling constants (*J*) are given in Hz. Proton multiplicity was assigned using the following abbreviations: singlet (s), doublet (d), triplet (t), quartet (q), quintet (quint.), septet (sept.), and multiplet (m); additionally, the abbreviation for broad (br) was used. Infrared measurements were carried out neat on a Bruker Vector 22 FT-IR spectrometer fitted with a Specac diamond attenuated total reflectance (ATR) module. MS analyses were carried out on a Waters Xevo TQD spectrometer with an electrospray ionization (ESI) ion source (Waters Corporation, Milford, MA, USA). HRMS analyses were carried out on a maXis plus ESI-Q-TOF mass spectrometer (Bruker Daltonics). The detailed synthetic procedures and spectral characterizations are described below.

General procedure 1 (GP1) for the synthesis of Alkyl succinate monoesters (S1-S11):

The alkyl succinate monoesters were prepared using our previously published methodology [1,2]. An oven-dried vial (20 mL) fitted with a screw cap and containing a magnetic stirrer was flushed with argon and charged with prop-2-yn-1-ol (104 µL, 3.44 mmol), and *N,N*-diisopropylethylamine (DIPEA) (466 µL, 2.68 mmol, 0.78 equiv.) and stirred in dichloromethane (DCM) (4 mL) at room temperature (RT). After 2 h, succinic anhydride (267.8 mg, 2.68 mmol, 0.78 equiv.) and 4-dimethylaminopyridine (DMAP) (326.9 mg, 2.68 mmol, 0.78 equiv.) were added, and the reaction was stirred at RT. After 48 h, the reaction mixture was diluted with brine/1 M HCl (3:1, 10 mL). The aqueous layer was extracted with DCM (3 × 20 mL), and the combined organic phases were dried over anhydrous sodium sulfate (Na_2SO_4) and concentrated under reduced pressure to obtain the desired product (Figure S1).

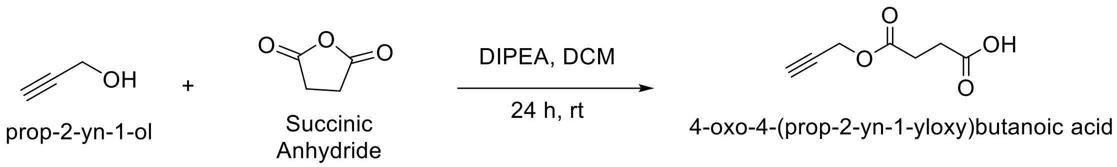

Figure S1. Methodology of synthesis of Alkyl succinate monoester (S1-S11).

In our recent publication [2], the following alkyl succinate monoesters (Figure S2) generated with this synthesis procedure were very well described, and because these compounds were produced on a large scale, they were used to bind with tetrahydrocurcumin: 4-((adamantan-2-yl)oxy)-4-oxobutanoic acid (S1), 4-(benzhydryloxy)-4-oxobutanoic acid (S3), 4-((9H-fluoren-9-yl)oxy)-4-oxobutanoic acid (S5), 4-((2,3-dihydro-1H-inden-2-yl)oxy)-4-oxobutanoic acid (S6), 4-(((1R,2S,5R)-2-isopropyl-5-methylcyclohexyl)oxy)-4-oxobutanoic acid (S7), 4-methoxy-4-oxobutanoic acid (S8), and 4-oxo-4-((1,2,3,4-tetrahydronaphthalen-1-yl)oxy)butanoic acid (S9).

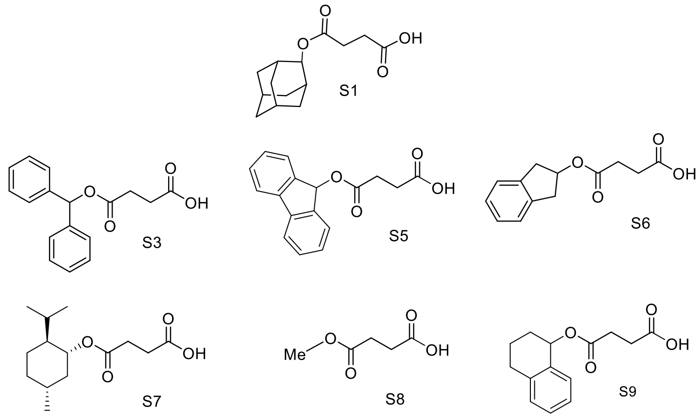

Figure S2. Alkyl succinate monoesters have been synthesized on a large scale [2].

4-oxo-4-(prop-2-yn-1-yloxy)butanoic acid (S2)

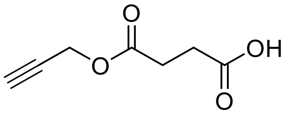

According to GP1, prop-2-yn-1-ol (104 µL, 3.44 mmol) and DIPEA (466 µL, 2.68 mmol, 0.78 equiv.) were stirred in DCM (2 mL) at RT. After 2 h, succinic anhydride (267.8 mg, 2.68 mmol, 0.78 equiv.) and DMAP (326.9 mg, 2.68 mmol, 0.78 equiv.) were added, and the reaction mixture was stirred for 48 h at RT to yield monoester **S2** (339.3 mg, 63%). mp: 47.9 ± 0.2 °C. IR: 3286.1, 2954.3, 1711.9, 1419.5, 1206.6, 1152.1, 992.1 cm^{-1}. MS (m/z) calcd. for $C_7H_8NaO_4$: 179.13; found: 178.92 [$M+Na^+$]. ^{13}C NMR (126 MHz, DMSO): δ173.3, 171.6, 77.7, 51.8, 28.9, 28.6, 28.5 ppm.

4-(benzyloxy)-4-oxobutanoic acid (S4)

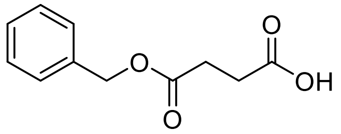

Phenylmethanol (481 µL, 500 mg, 4.62 mmol) and DIPEA (1200 µL, 6.94 mmol, 1.5 equiv.) were stirred in dichloromethane (DCM) (4 mL) at RT. After 2 h, succinic anhydride (694 mg, 6.93 mmol, 1.5 equiv.) and 4-dimethylaminopyridine (DMAP) (847.3 mg, 6.93 mmol, 1.50 equiv.) were added, and the reaction mixture was stirred for 48 h at RT to yield monoester **S4** (842.2 mg, 87%). IR: 2928.7, 1683.9, 1682.3, 1419.0, 1357.7, 1172.2, 731.5 cm^{-1}. mp: 60.5 ± 0.2 °C. MS (m/z) calcd. for $C_{11}H_{12}NaO_4$: 231.2; found: 231.9 [$M+Na^+$].

4-(cyclopentyloxy)-4-oxobutanoic acid (S10)

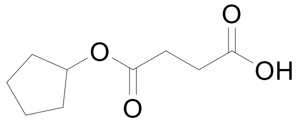

Cyclopentanol (527 µL, 5.80 mmol) and DIPEA (1516 µL, 8.71 mmol, 1.5 equiv.) were stirred in DCM (4 mL) at RT. After 2 h, succinic anhydride (871.3 mg, 8.71 mmol, 1.5 equiv.) and DMAP (1063.8 mg, 8.71 mmol, 1.5 equiv.) were added, and the reaction mixture was stirred for 48 h at RT to yield monoester **S10** (704.5 mg, 65%). IR: 2962.9, 2875.5, 1716.9, 1227.6, 1156.6, 936.6 cm^{-1}. mp: 37.3 ± 0.5 °C. MS (m/z) calcd. for $C_9H_{14}NaO_4$: 209.2; found: 209.9 [$M+Na^+$].

4-(allyloxy)-4-oxobutanoic acid (S11)

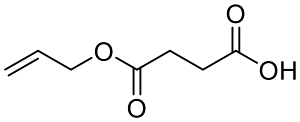

Prop-2-en-1-ol (585.5 µL, 8.61 mmol) and DIPEA (2249.2 µL, 12.9 mmol, 1.5 equiv.) were stirred in DCM (4 mL) at RT. After 2 h, succinic anhydride (1292.2 mg, 12.9 mmol, 1.5 equiv.) and DMAP (1577.6 mg, 12.9 mmol, 1.5 equiv.) were added, and the reaction mixture was stirred for 48 h at RT to yield monoester **S11** (677.1 mg, 50%). IR: 2989.2, 2929.8, 1699.1, 1680.3, 1418.4, 1304.9,1195.1, 906.6 cm^{-1}. MS (m/z) calcd. for $C_7H_{10}NaO_4$: 181.14; found: 180.92 [$M+Na^+$].

General Procedure 2 (GP2) of Synthesis of Tetrahydrocurcumin Derivatives

The tetrahydrocurcumin derivatives were prepared using a previously published methodology [5,6]. A borosilicate glass tube (16 ml) that was fitted with a screw cap and contained a magnetic stirrer was flushed with argon and charged with alkyl succinate (173.2 mg, 0.96 mmol, 1.8 equiv.), 4-dimethylaminopyridine (DMAP) (118.2 mg, 0.96 mmol, 1.8 equiv.), tetrahydrocurcumin (200 mg, 0.53 mmol) and dichloromethane (DCM) (3 mL); after combining, the reagents were stirred at 0 °C for 10 min. Then, N-(3-dimethylaminopropyl)-N′-ethylcarbodiimide hydrochloride (EDC) (185.4 mg, 0.96 mmol, 1.8 equiv.) was added, and the mixture was stirred at RT for 24 h. The reaction mixture was extracted with EtOAc and water (3 × 20 mL). The combined organic phases were concentrated under reduced pressure, and the remaining material was purified by HPLC to obtain the desired tetrahydrocurcumin derivative (Figure S3).

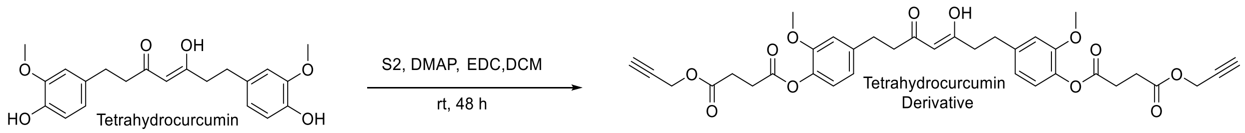

Figure S3. Methodology of synthesis of tetrahydrocurcumin derivatives (3-13)

(Z)-O,O'-((3-hydroxy-5-oxohept-3-ene-1,7-diyl)bis(2-methoxy-4,1-phenylene)) di(prop-2-yn-1-yl) disuccinate (3)

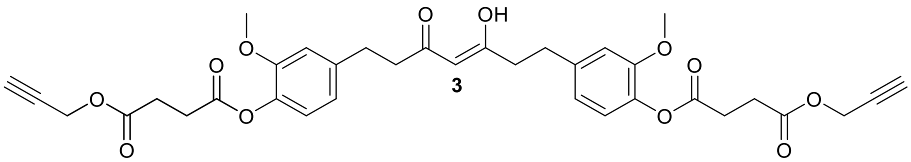

According to GP2, reaction of succinate S2, (173.2 mg, 0.96 mmol, 1.8 equiv.), DMAP (118.2 mg, 0.96 mmol, 1.8 equiv.), tetrahydrocurcumin (200 mg, 0.53 mmol), and DCM (3 mL) was performed with stirring at 0 °C for 10 min. Then, EDC (185.4 mg, 0.96 mmol, 1.8 equiv.) was added, and the mixture was stirred at RT for 24 h. The crude product was purified by prep-TLC on silica gel (EtOAc/hexane, 4:6 v/v) to give compound **3** (243.7 mg, 70%). ^{1}H NMR (500 MHz, $CDCl_3$): 6.99–6.91 (m, 2H), 6.82–6.74 (m, 4H), 5.45 (s, 1H), 4.74 (d, *J* = 2.4 Hz, 4H), 3.87 (s, 2H), 3.81 (s, 6H), 2.98–2.89 (m, 8H), 2.82 (t, *J* = 6.9 Hz, 4H), 2.63–2.57 (m, 4H) ppm. ^{13}C NMR (126 MHz, $CDCl_3$): 193.0, 171.4, 170.5, 150.9, 139.8, 138.1, 122.7, 120.5, 112.7, 99.9, 77.6, 77.2, 75.2, 56.0, 52.4, 40.1, 31.5, 29.1, 28.9 ppm. IR: 3276.0, 2933.7, 1742.7, 1602.9, 1510.1, 1451.4, 1418.5, 1366.7, 1266.5, 1200.9, 1135.0 cm^{-1}. HRMS (m/z): calcd. for $C_{35}H_{36}O_{12}$ $[M+Na^+]^+$: 671.2099, found: 671.2084.

(Z)-di(9H-fluoren-9-yl) O,O'-((3-hydroxy-5-oxohept-3-ene-1,7-diyl)bis(2-methoxy-4,1-phenylene)) disuccinate (4).

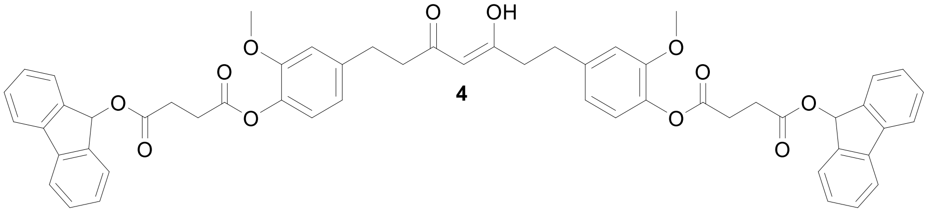

According to GP2, reaction of succinate S5 (295.2 mg, 0.96 mmol, 1.8 equiv.), DMAP (118.2 mg, 0.96 mmol, 1.8 equiv.), tetrahydrocurcumin (200 mg, 0.53 mmol), and dichloromethane (3 mL) was performed with stirring at 0 °C for 10 min. Then, EDC (185.4 mg, 0.96 mmol, 1.8 equiv.) was added, and the mixture was stirred at RT for 24 h. The crude product was purified by prep-TLC on silica gel (EtOAc/hexane, 4:6 v/v) to give compound **4** (263.9 mg, 55%). ^{1}H NMR (500 MHz, $CDCl_3$): δ 7.67 (d, *J* = 7.6 Hz, 4H), 7.54 (d, *J* = 7.5 Hz, 4H), 7.41 (t, *J* = 7.5 Hz, 4H), 7.27 (t, *J* = 7.4 Hz, 4H), 6.90 (d, *J* = 7.9 Hz, 2H), 6.85 (s, 2H), 6.81–6.70 (m, 4H), 5.48–5.43 (m, 1H), 3.77 (s, 6H), 2.99 (t, *J* = 6.9 Hz, 4H), 2.93–2.85 (m, 8H), 2.63–2.54 (m, 4H) ppm. ^{13}C NMR (126 MHz, $CDCl_3$): δ 193.0, 172.9, 170.6, 150.9, 142.0, 141.1, 139.8, 138.1, 129.6, 128.0, 126.0, 122.8, 120.4, 120.1, 114.4, 112.7, 111.0, 99.9, 75.5, 55.9, 40.1, 31.5, 31.4, 29.7, 29.2 ppm. IR: 2934.5, 1758.5, 1731.6, 1602.2, 1509.6, 1451.5, 1417.9, 1361.5, 1239.1, 1200.2, 1132.3 cm^{-1}. HRMS (m/z): calcd. for $C_{55}H_{48}O_{12}$ $[M+Na^+]^+$: 923.3038, found: 923.3018.

(Z)-bis(2,3-dihydro-1H-inden-2-yl) O,O'-((3-hydroxy-5-oxohept-3-ene-1,7-diyl)bis(2-methoxy-4,1-phenylene)) disuccinate (5)

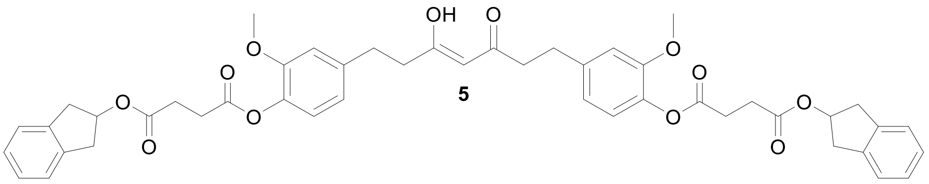

According to GP2, reaction of succinate S6 (248.5 mg, 0.96 mmol, 1.8 equiv.), DMAP (118.2 mg, 0.96 mmol, 1.8 equiv.), tetrahydrocurcumin (200 mg, 0.53 mmol), and DCM (3 mL) was performed with stirring at 0 °C for 10 min. Then, EDC (185.4 mg, 0.96 mmol, 1.8 equiv.) was added, and the mixture was stirred at RT for 24 h. The crude product was purified by prep-TLC on silica gel (EtOAc/hexane, 4 : 6 v/v) to give the compound **5** (181.4 mg, 42%).^{1}H NMR (500 MHz, $CDCl_3$): δ 7.26–7.16 (m, 8H), 6.90 (d, *J* = 8.1 Hz, 2H), 6.80–6.69 (m, 4H), 5.62–5.54 (m, 2H), 5.45 (s, 1H), 3.78 (s, 6H), 3.33 (dd, *J* = 17.0, 6.5 Hz, 4H), 3.04 (d, *J* = 17.0 Hz, 4H), 2.93–2.86 (m, 8H), 2.70 (t, *J* = 7.0 Hz, 4H), 2.62–2.53 (m, 4H) ppm. ^{13}C NMR (126 MHz, $CDCl_3$): δ 193.0, 172.1, 170.7, 150.9, 140.4, 139.7, 138.1, 126.9, 124.7, 122.7, 122.7, 120.4, 120.4, 112.7, 99.9, 75.7, 55.9, 40.0, 39.6, 31.5, 31.4, 29.5, 29.0 ppm. IR: 2936.0, 1759.3, 1728.4, 1602.0, 1509.8, 1459.8, 1418.4, 1266.2, 1200.8, 1133.0 cm^{-1}. HRMS (m/z): calcd. for $C_{47}H_{48}O_{12}$ [M+Na^{+}]$^{+}$: 827.3038, found: 827.3024.

(Z)-dibenzhydryl O,O'-((3-hydroxy-5-oxohept-3-ene-1,7-diyl)bis(2-methoxy-4,1-phenylene)) disuccinate (6)

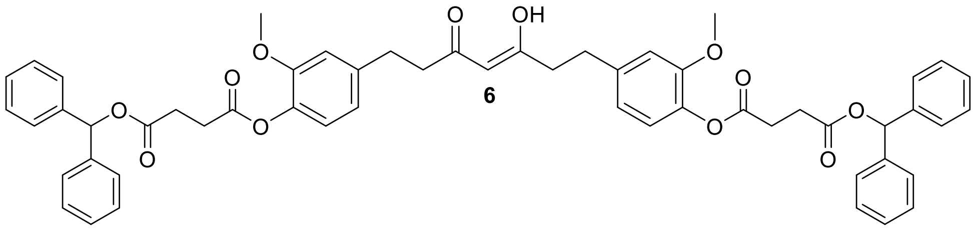

According to GP2, reaction of succinate S3 (297.2 mg, 0.96 mmol, 1.8 equiv.), DMAP (118.2 mg, 0.96 mmol, 1.8 equiv.), tetrahydrocurcumin (200 mg, 0.53 mmol), and DCM (3 mL) was performed with stirring at 0 °C for 10 min. Then, EDC (185.4 mg, 0.96 mmol, 1.8 equiv.) was added, and the mixture was stirred at RT for 24 h. The crude product was purified by prep-TLC on silica gel (EtOAc/hexane, 4:6 v/v) to give compound **6** (70.5 mg, 15%). ^{1}H NMR (500 MHz, $CDCl_3$): δ 7.43–7.25 (m, 20H), 6.95 (s, 2H), 6.87 (d, *J* = 8.1 Hz, 2H), 6.82–6.70 (m, 4H), 5.47 (s, 1H), 3.77 (s, 6H), 2.99–2.86 (m, 13H), 2.61 (t, *J* = 7.9 Hz, 4H) ppm. ^{13}C NMR (126 MHz, $CDCl_3$): δ 193.0, 171.1, 170.5, 150.9, 140.1, 139.7, 138.1, 128.7, 128.6, 128.6, 128.0, 127.2, 127.1, 122.8, 122.7, 120.4, 112.6, 99.9, 77.4, 77.4, 55.9, 40.1, 31.5, 29.7, 29.1 ppm. IR: 2934.8, 1734.5, 1602.1, 1509.9, 1453.2, 1418.1, 1362.5, 1266.9, 1201.1, 1134.2 cm^{-1}. HRMS (m/z): calcd. for $C_{55}H_{52}O_{12}$ [M+Na^{+}]$^{+}$: 927.3351, found: 927.3334.

O,O'-(((Z)-3-hydroxy-5-oxohept-3-ene-1,7-diyl)bis(2-methoxy-4,1-phenylene)) bis((1R,2S,5R)-2-isopropyl-5-methylcyclohexyl) disuccinate (7)

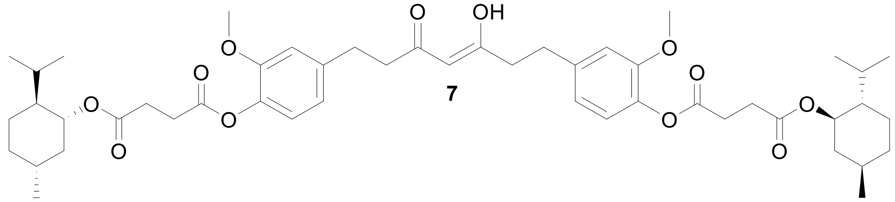

According to GP2, reaction of succinate S7 (269.9 mg, 0.96 mmol, 1.8 equiv.), DMAP (118.2 mg, 0.96 mmol, 1.8 equiv.), tetrahydrocurcumin (200 mg, 0.53 mmol), and DCM (3 mL) was performed with stirring at 0 °C for 10 min. Then, EDC (185.4 mg, 0.96 mmol, 1.8 equiv.) was added, and the mixture was stirred at RT for 24 h. The crude product was purified by prep-TLC on silica gel (EtOAc/hexane, 4:6 v/v) to give compound **7** (57.4 mg, 13%). ^{1}H NMR (500 MHz, $CDCl_3$): δ 6.94 (d, *J* = 7.9 Hz, 2H), 6.80–6.69 (m, 4H), 5.44 (s, 1H), 4.76–4.67 (m, 2H), 3.79 (s, 6H), 2.90 (t, *J* = 7.9 Hz, 8H), 2.78–2.50 (m, 8H), 1.99 (d, *J* = 11.9 Hz, 2H), 1.91–1.82 (m, 2H), 1.72–1.22 (m, 10H), 1.09–0.93 (m, 4H), 0.88 (t, *J* = 7.0 Hz, 12H), 0.74 (d, *J* = 6.9 Hz, 6H) ppm. ^{13}C NMR (126 MHz, $CDCl_3$): δ 193.0, 171.7, 170.7, 151.0, 139.8, 138.2, 122.8, 122.8, 120.5, 112.7, 99.9, 77.4, 77.2, 76.9, 74.8, 56.0, 47.1, 41.0, 40.1, 34.4, 31.5, 31.5, 29.7, 29.2, 26.4, 23.6, 22.1, 20.9, 16.4 ppm. IR: 2953.8, 2869.5, 1763.3, 1729.2, 1604.1, 1510.9, 1454.5, 1418.8, 1367.9, 1137.6 cm^{-1}. HRMS (m/z): calcd. for $C_{49}H_{68}O_{12}$ [M+Na$^+$]$^+$: 871.4603, found: 871.4583.

(Z)-O,O'-((3-hydroxy-5-oxohept-3-ene-1,7-diyl)bis(2-methoxy-4,1-phenylene)) bis(1,2,3,4-tetrahydronaphthalen-1-yl) disuccinate (8)

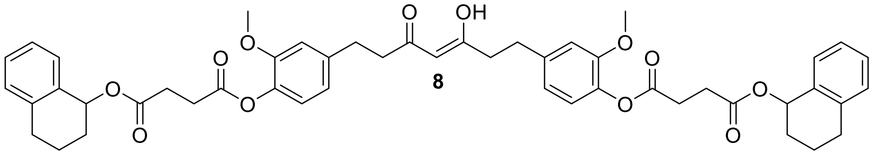

According to GP2, reaction of succinate S9 (262.3 mg, 0.96 mmol, 1.8 equiv.), DMAP (118.2 mg, 0.96 mmol, 1.8 equiv.), tetrahydrocurcumin (200 mg, 0.53 mmol), and DCM (3 mL) was performed with stirring at 0 °C for 10 min. Then, EDC (185.4 mg, 0.96 mmol, 1.8 equiv.) was added, and the mixture was stirred at RT for 24 h. The crude product was purified by prep-TLC on silica gel (EtOAc/hexane, 4:6 v/v) to give compound **8** (224.7 mg, 50%). ^{1}H NMR (500 MHz, $CDCl_3$): δ 7.30–7.07 (m, 8H), 6.88 (d, *J* = 8.0 Hz, 2H), 6.79–6.70 (m, 4H), 6.09–6.01 (m, 2H), 5.45 (s, 1H), 3.78 (s, 6H), 2.96–2.84 (m, 10H), 2.76 (t, *J* = 7.1 Hz, 6H), 2.59 (t, *J* = 7.9 Hz, 4H), 2.04–1.92 (m, 6H), 1.88–1.76 (m, 2H) ppm. ^{13}C NMR (126 MHz, $CDCl_3$): δ 193.0, 171.8, 170.7, 151.0, 139.8, 138.1, 138.1, 134.5, 129.6, 129.2, 128.3, 126.2, 122.8, 122.8, 120.5, 112.7, 99.9, 70.6, 56.0, 40.1, 31.5, 29.9, 29.2, 29.1, 18.9 ppm. IR: 2935.6, 1760.0, 1727.7, 1603.4, 1510.4, 1453.7, 1418.5, 1266.7, 1201.7, 1135.5 cm^{-1}. HRMS (m/z): calcd. for $C_{49}H_{52}O_{12}$ [M+Na]$^+$: 855.3351, found: 855.3337.

(Z)-dibenzyl O,O'-((3-hydroxy-5-oxohept-3-ene-1,7-diyl)bis(2-methoxy-4,1-phenylene)) disuccinate (9)

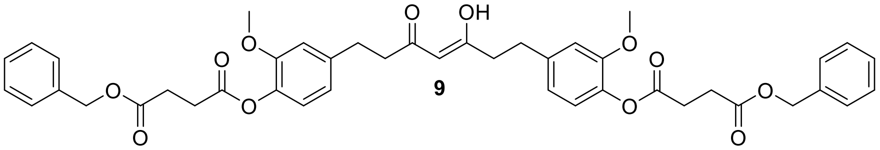

According to GP2, reaction of succinate S4 (201.3 mg, 0.96 mmol, 1.8 equiv.), DMAP (118.2 mg, 0.96 mmol, 1.8 equiv.), tetrahydrocurcumin (200 mg, 0.53 mmol), and DCM (3 mL) was performed with stirring at 0 °C for 10 min. Then, EDC (185.4 mg, 0.96 mmol, 1.8 equiv.) was added, and the mixture was stirred at RT for 24 h. The crude product was purified by prep-TLC on silica gel (EtOAc/hexane, 4:6 v/v) to give compound **9** (261.1 mg, 65%). ^{1}H NMR (500 MHz, $CDCl_3$): δ 7.36 (s, 9H), 6.90 (d, *J* = 8.0 Hz, 2H), 6.80–6.69 (m, 4H), 5.44 (s, 1H), 5.16 (s, 4H), 3.77 (s, 6H), 2.98–2.85 (m, 8H), 2.82–2.79 (m, 4H), 2.62–2.54 (m, 4H) ppm. ^{13}C NMR (126 MHz, $CDCl_3$): δ 193.0, 172.0, 170.6, 150.9, 139.8, 138.1, 135.9, 128.7, 128.4, 128.3, 122.7, 120.4, 114.4, 112.7, 111.0, 99.9, 77.2, 66.7, 55.9, 40.1, 31.5, 29.4, 29.1 ppm. IR: 2935.8, 1759.2, 1732.5, 1603.2, 1511.0, 1454.9, 1418.5, 1267.3, 1201.6, 1135.7 cm^{-1}. HRMS (m/z): calcd. for $C_{43}H_{44}O_{12}$ $[M+Na^+]^+$: 775.2725, found: 775.2710.

(Z)-dicyclopentyl O,O'-((3-hydroxy-5-oxohept-3-ene-1,7-diyl)bis(2-methoxy-4,1-phenylene)) disuccinate (10)

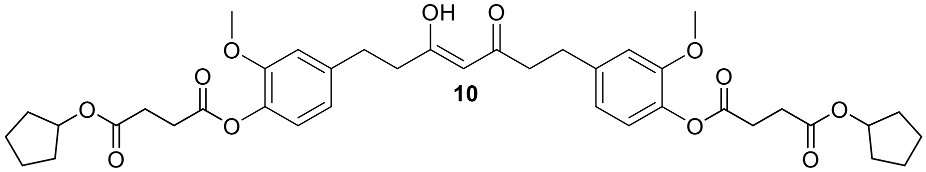

According to GP2, reaction of succinate S10 (180.0 mg, 0.96 mmol, 1.8 equiv.), DMAP (118.2 mg, 0.96 mmol, 1.8 equiv.), tetrahydrocurcumin (200 mg, 0.53 mmol), and DCM (3 mL) was performed with stirring at 0 °C for 10 min. Then, EDC (185.4 mg, 0.96 mmol, 1.8 equiv.) was added, and the mixture was stirred at RT for 24 h. The crude product was purified by prep-TLC on silica gel (EtOAc/hexane, 4:6 v/v) to give compound **10** (147.7 mg, 39%). ^{1}H NMR (500 MHz, $CDCl_3$): δ 6.93 (d, *J* = 7.9 Hz, 2H), 6.79–6.69 (m, 4H), 5.43 (s, 1H), 5.25–5.13 (m, 2H), 3.78 (s, 6H), 2.93–2.82 (m, 8H), 2.68 (t, *J* = 7.0 Hz, 4H), 2.57 (t, *J* = 7.9 Hz, 4H), 1.90–1.78 (m, 4H), 1.75–1.63 (m, 8H), 1.64–1.51 (m, 4H) ppm. ^{13}C NMR (126 MHz, $CDCl_3$): δ 193.0, 171.9, 170.7, 150.9, 139.7, 138.1, 122.7, 120.4, 112.7, 99.9, 77.6, 55.9, 40.0, 32.7, 31.5, 29.6, 29.1, 23.8 ppm. IR: 2959.2, 1760.7, 1730.0, 1603.9, 1510.8, 1418.7, 1267.3, 1202.2, 1136.7 cm^{-1}. HRMS (m/z): calcd. for $C_{39}H_{48}O_{12}$ $[M+Na^+]^+$: 731.3038, found: 731.3033.

(Z)-O,O'-((3-hydroxy-5-oxohept-3-ene-1,7-diyl)bis(2-methoxy-4,1-phenylene)) dimethyl disuccinate (11)

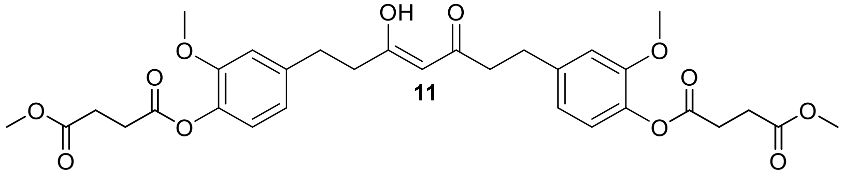

According to GP2, reaction of succinate S8 (149.8 mg, 0.96 mmol, 1.8 equiv.), DMAP (118.2 mg, 0.96 mmol, 1.8 equiv.), tetrahydrocurcumin (200 mg, 0.53 mmol), and DCM (3 mL) was performed with stirring at 0 °C for 10 min. Then, EDC (185.4 mg, 0.96 mmol, 1.8 equiv.) was added, and the mixture was stirred at RT for 24 h. The crude product was purified by prep-TLC on silica gel (EtOAc/hexane, 4:6 v/v) to give compound **11** (189.6 mg, 59%). ^{1}H NMR (500 MHz, $CDCl_3$) δ 6.94 (d, *J* = 8.0 Hz, 2H), 6.79–6.69 (m, 4H), 5.42 (s, 1H), 3.78 (s, 6H), 3.71 (s, 6H), 2.94–2.84 (m, 8H), 2.74 (t, *J* = 7.0 Hz, 4H), 2.60–2.52 (m, 4H) ppm. ^{13}C NMR (126 MHz, $CDCl_3$): δ 193.0, 172.6, 170.7, 150.9, 139.8, 138.1, 122.7, 122.7, 120.4, 120.4, 112.7, 99.9, 77.4, 77.2, 76.9, 55.9, 52.0, 40.1, 31.5, 29.1, 29.1 ppm. IR: 2950.2, 1758.7, 1733.1, 1602.6, 1510.26, 1418.4, 1267.2, 1201.0, 1135.4, 1033.8 cm^{-1}. HRMS (m/z): calcd. for $C_{31}H_{36}O_{12}$ [M+Na^{+}]$^{+}$: 623.2099, found: 623.2095.

(Z)-diallyl O,O'-((3-hydroxy-5-oxohept-3-ene-1,7-diyl)bis(2-methoxy-4,1-phenylene)) disuccinate (12)

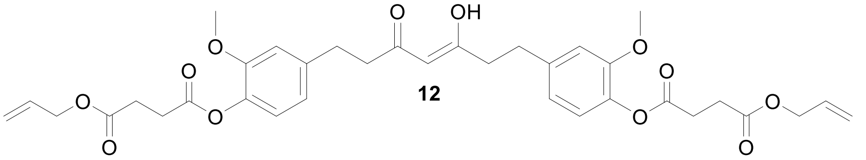

According to GP2, reaction of succinate S11 (152.9 mg, 0.96 mmol, 1.8 equiv.), 4-dimethylaminopyridine (118.2 mg, 0.96 mmol, 1.8 equiv.), tetrahydrocurcumin (200 mg, 0.53 mmol), and dichloromethane (3 mL) was performed with stirring at 0 °C for 10 min. Then, N-(3-dimethylaminopropyl)-N′-ethylcarbodiimide hydrochloride (185.4 mg, 0.96 mmol, 1.8 equiv.) was added, and the mixture was stirred at RT for 24 h. The crude product was purified by prep-TLC on silica gel (EtOAc/hexane, 4:6 v/v) to give compound **12** (23.9 mg, 7%). ^{1}H NMR (500 MHz, $CDCl_3$): 6.94 (d, *J* = 7.9 Hz, 2H), 6.79–6.67 (m, 4H), 5.97–5.86 (m, 2H), 5.43 (s, 1H), 5.32 (d, *J* = 17.1 Hz, 2H), 5.24 (d, *J* = 8.8 Hz, 2H), 4.62 (d, *J* = 5.7 Hz, 4H), 3.79 (s, 6H), 2.95–2.85 (m, 8H), 2.77 (t, *J* = 7.0 Hz, 4H), 2.57 (t, *J* = 7.8 Hz, 4H) ppm. ^{13}C NMR (126 MHz, $CDCl_3$) δ 193.0, 171.8, 170.7, 151.0, 139.8, 138.1, 132.1, 122.8, 122.8, 120.5, 120.4, 118.5, 112.7, 99.9, 65.6, 56.0, 40.1, 31.5, 31.5, 29.3, 29.1 ppm. IR: 2935.5, 1760.2, 1735.6, 1604.2, 1511.5, 1453.1, 1419.1, 1363.0, 1273.8, 1138.0 cm^{-1}. HRMS (m/z): calcd. for $C_{35}H_{40}O_{12}$ [M+Na^{+}]$^{+}$: 675.2412, found: 675.2406.

di((1r,3r,5r,7r)-adamantan-2-yl) O,O'-(((Z)-3-hydroxy-5-oxohept-3-ene-1,7-diyl)bis(2-methoxy-4,1-phenylene)) disuccinate (13)

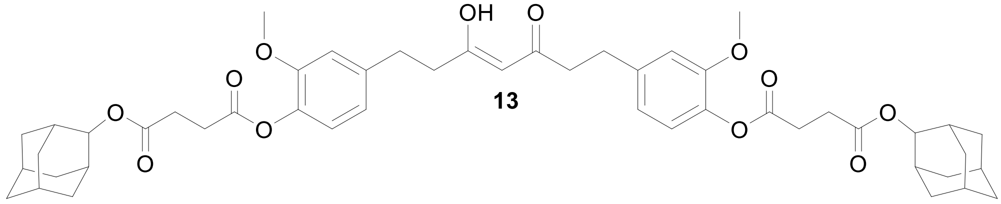

According to GP2, reaction of succinate S1 (244.1 mg, 0.96 mmol, 1.8 equiv.), DMAP (118.2 mg, 0.96 mmol, 1.8 equiv.), tetrahydrocurcumin (200 mg, 0.53 mmol), and DCM (3 mL) was performed with stirring at 0 °C for 10 min. Then, EDC (185.4 mg, 0.96 mmol, 1.8 equiv.) was added, and the mixture was stirred at RT for 24 h. The crude product was purified by prep-TLC on silica gel (EtOAc/hexane, 4:6 v/v) to give compound **13** (52.7 mg, 12%). ^{1}H NMR (500 MHz, $CDCl_3$): δ 6.93 (d, *J* = 8.0 Hz, 2H), 6.79–6.69 (m, 4H), 5.43 (s, 1H), 4.96 (s, 2H), 3.79 (s, 6H), 2.96–2.83 (m, 8H), 2.77 (t, *J* = 7.0 Hz, 4H), 2.57 (t, *J* = 7.7 Hz, 3H), 2.06–1.96 (m, 8H), 1.87–1.71 (m, 16H), 1.58–1.51 (m, 4H) ppm. ^{13}C NMR (126 MHz, $CDCl_3$): δ 193.0, 171.4, 170.8, 151.0, 139.7, 138.1, 122.8, 122.7, 120.4, 112.7, 99.9, 77.6, 55.9, 40.1, 37.5, 36.4, 31.9, 31.9, 31.5, 29.9, 29.2, 27.3, 27.1 ppm. IR: 2906.6, 2854.6, 1762.4, 1728.8, 1603.6, 1510.2, 1451.2, 1360.1, 1266.8, 1201.8, 1136.8 cm^{-1}. HRMS (m/z): calcd. for $C_{49}H_{60}O_{12}$ [M+Na^{+}]$^{+}$: 863.3977, found: 863.3967.

Table S1. Structure of the 23 compounds used for the generation of the 3D-QSAR model and their anti-inflammatory activity.

COMPOUNDS	STRUCTURE	IC50 ± S.D. (uM) IL-6
1	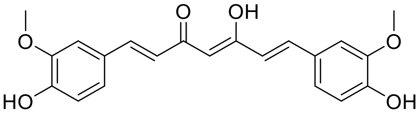	**6.85 ± 2.50**
2	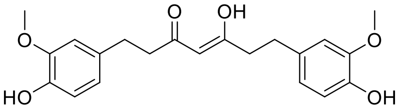	0.17 ± 0.20
4	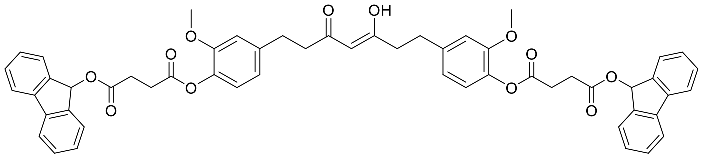	4.28 ± 4.88
5	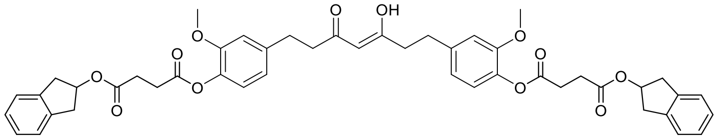	9.13 ± 5.90
8	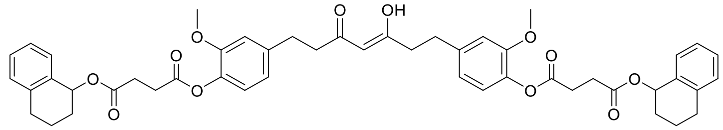	3.48 ± 4.39
10	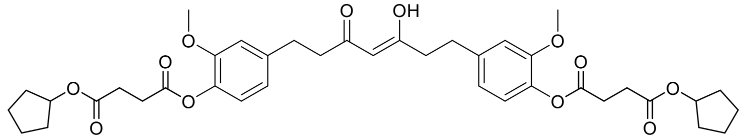	3.66 ± 4.21
11	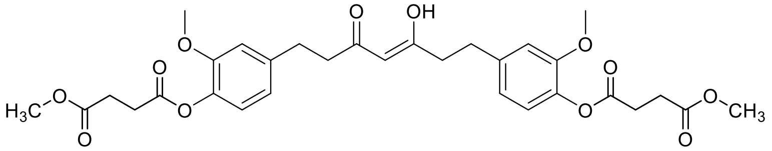	0.17 ± 0.21
12	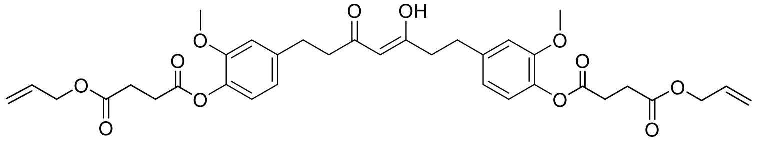	0.72 ± 0.38
13	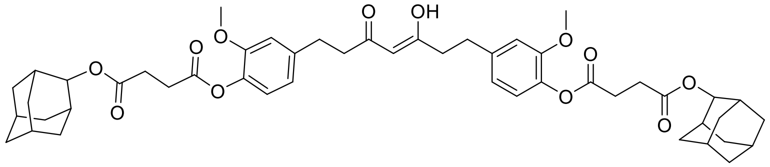	1.83 ± 2.55
14	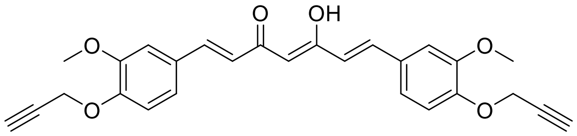	2.23±0.84
15	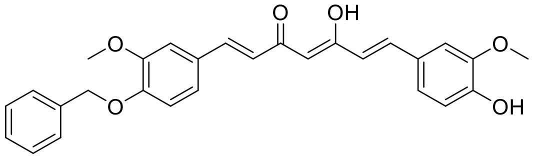	**3.59 ± 0.27**

16	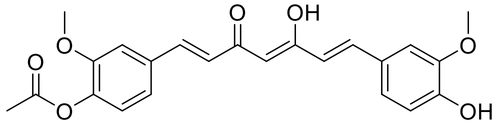	2.21±0.93
17	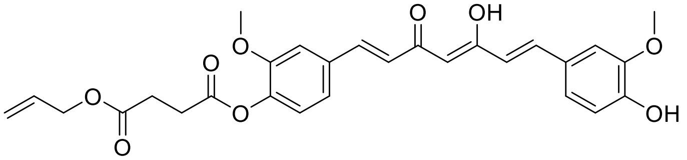	2.50±0.92
18	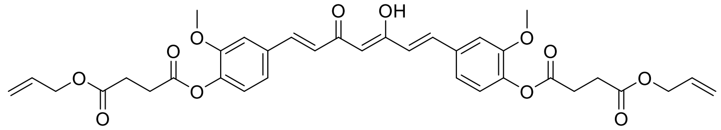	14.2±12.8
19	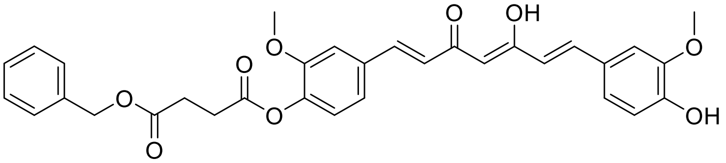	8.28±3.08
20	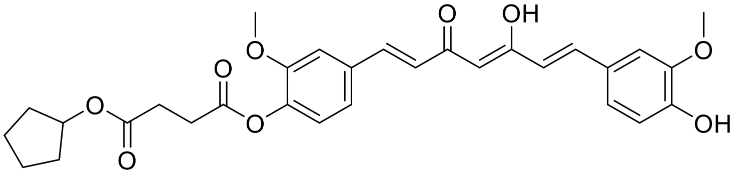	3.22±1.34
21	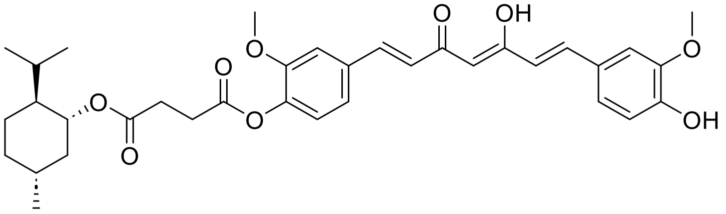	10.6 ± 0.33
22	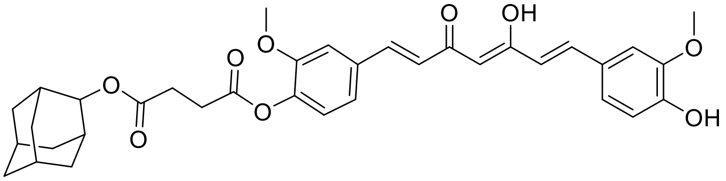	10.5 ± 0.6
23	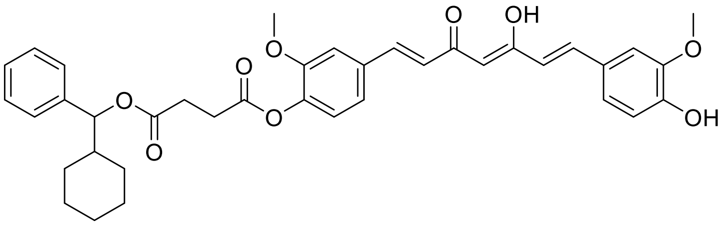	1.94 ± 0.66
24	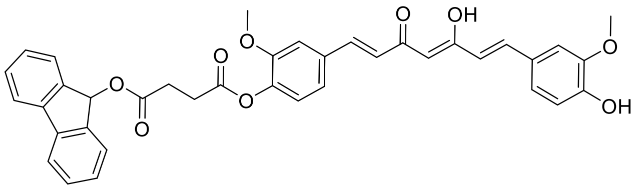	3.60 ± 0.21

25	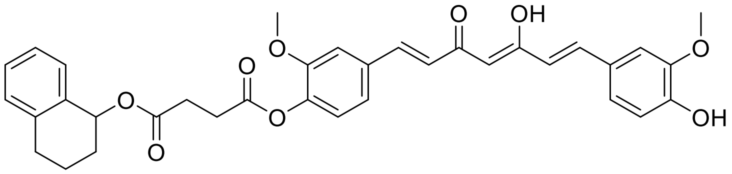	10.4 ± 0.75
26	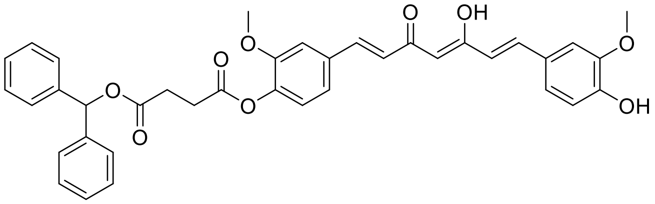	4.21 ± 0.73
27	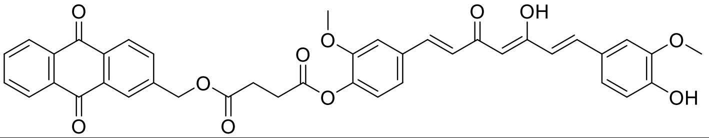	7.11 ± 0.75

Tabla S2. Experimental and predicted activities (pIC_{50}) of the Test set compounds and Residual Values

Compound	Experimental pIC_{50}	Predicted pIC_{50}	Residues
1	5,16	5,73	-0,57
5	5,04	6,05	-1,01
13	5,74	5,35	0,39
17	5,60	5,35	0,25
22	4,98	5,29	-0,32
23	5,71	5,35	0,36
25	4,98	5,38	-0,39

Figure S4. CoMFA contour maps were based on compound **2** as the template

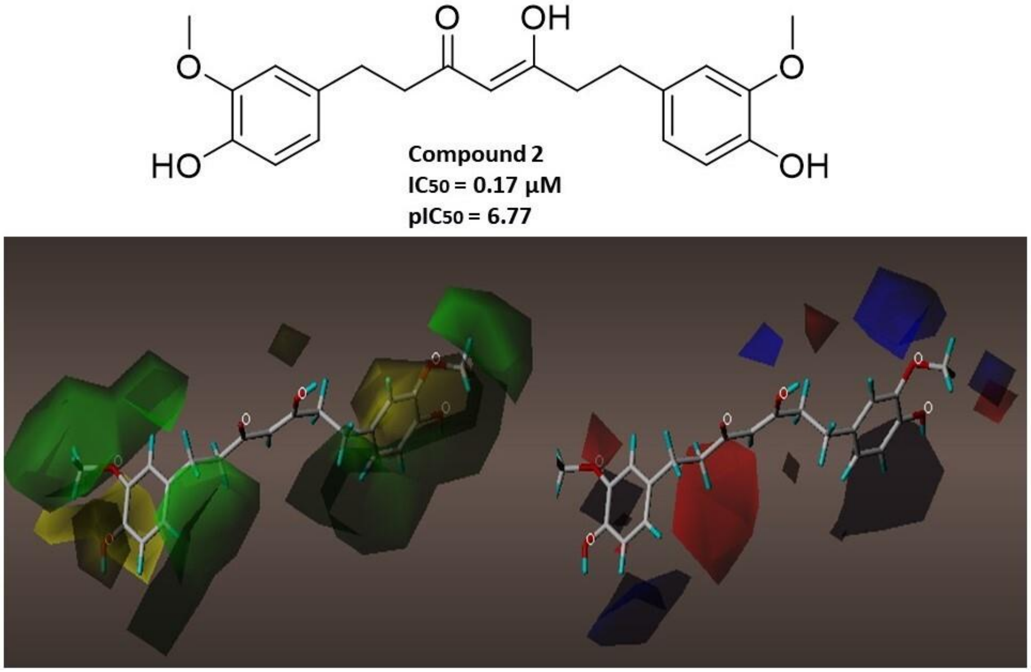

Figure S5. CoMFA contour maps were based on compound 12 as the template

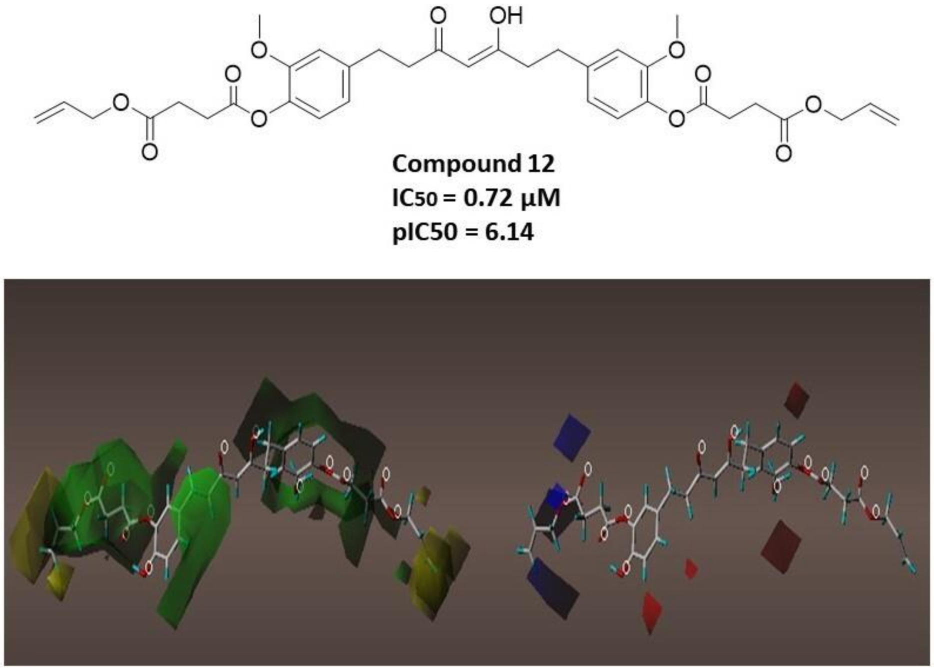

Figure S6. CoMFA contour maps were based on compound 1 as the template

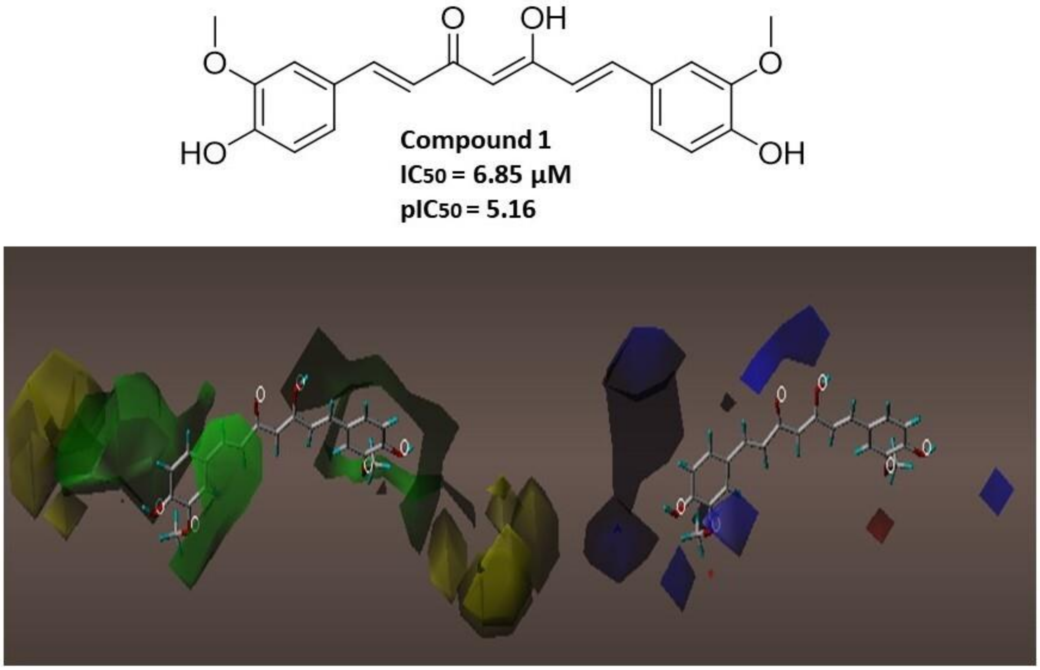

Reference

1. Lakey-Beitia, J.; Gonzalez, Y.; Doens, D.; Stephens, D.E.; Santamaria, R.; Murillo, E.; Gutierrez, M.; Fernandez, P.L.; K.S.Rao; Larionov, O. V.; et al. Assessment of Novel Curcumin Derivatives as Potent Inhibitors of Inflammation and Amyloid-Beta Aggregation in Alzheimer Disease. *J Alzheimers Dis* **2017**, *2017*, 1–10.

2. González, Y.; Mojica-Flores, R.; Moreno-Labrador, D.; Cubilla-Rios, L.; Rao, K.S.J.; Fernández, P.L.; Larionov, O. V.; Lakey-Beitia, J. Polyphenols with Anti-Inflammatory Properties: Synthesis and Biological Activity of Novel Curcumin Derivatives. *Int. J. Mol. Sci.* **2023**, *24*, 3691.

4-oxo-4-(prop-2-yn-1-yloxy)butanoic acid (S2)

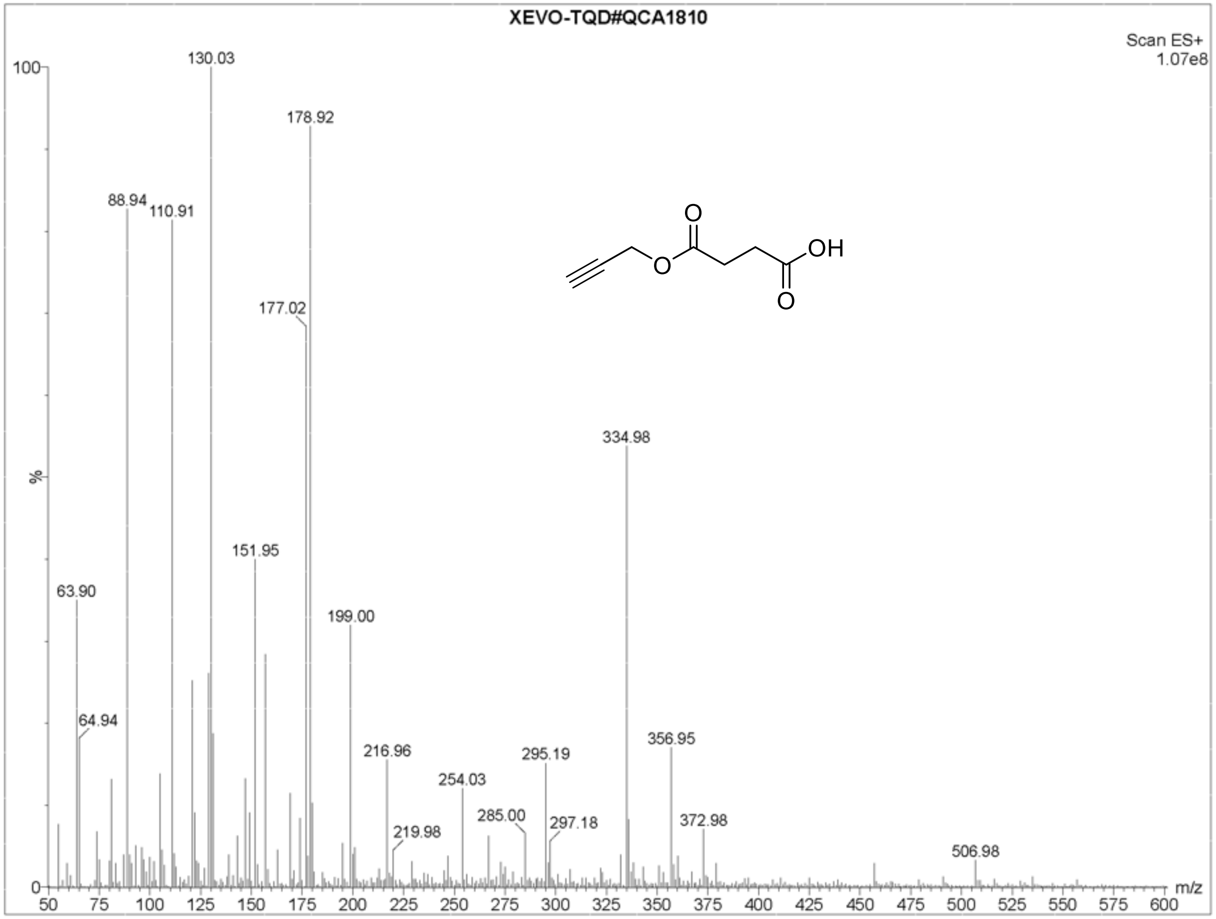

4-oxo-4-(prop-2-yn-1-yloxy)butanoic acid (S2)

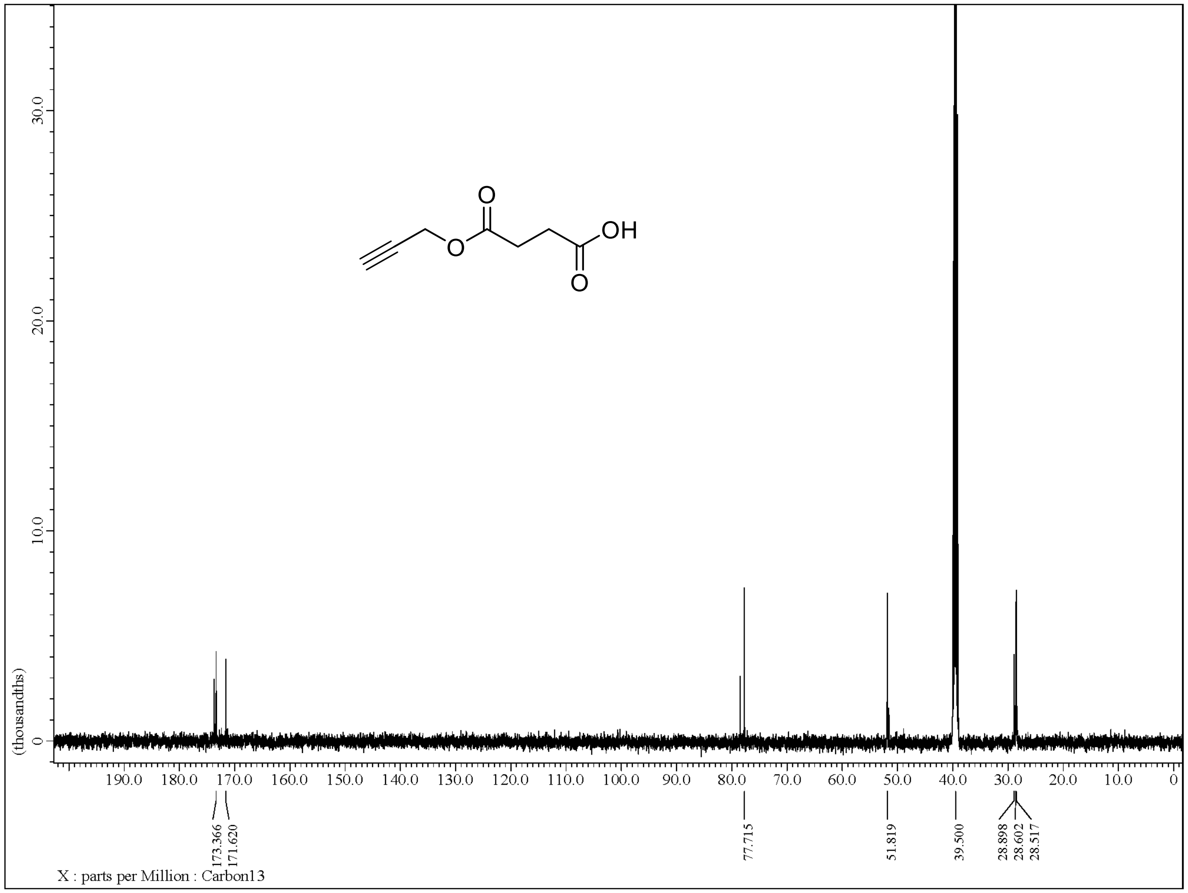

4-(benzyloxy)-4-oxobutanoic acid (S4)

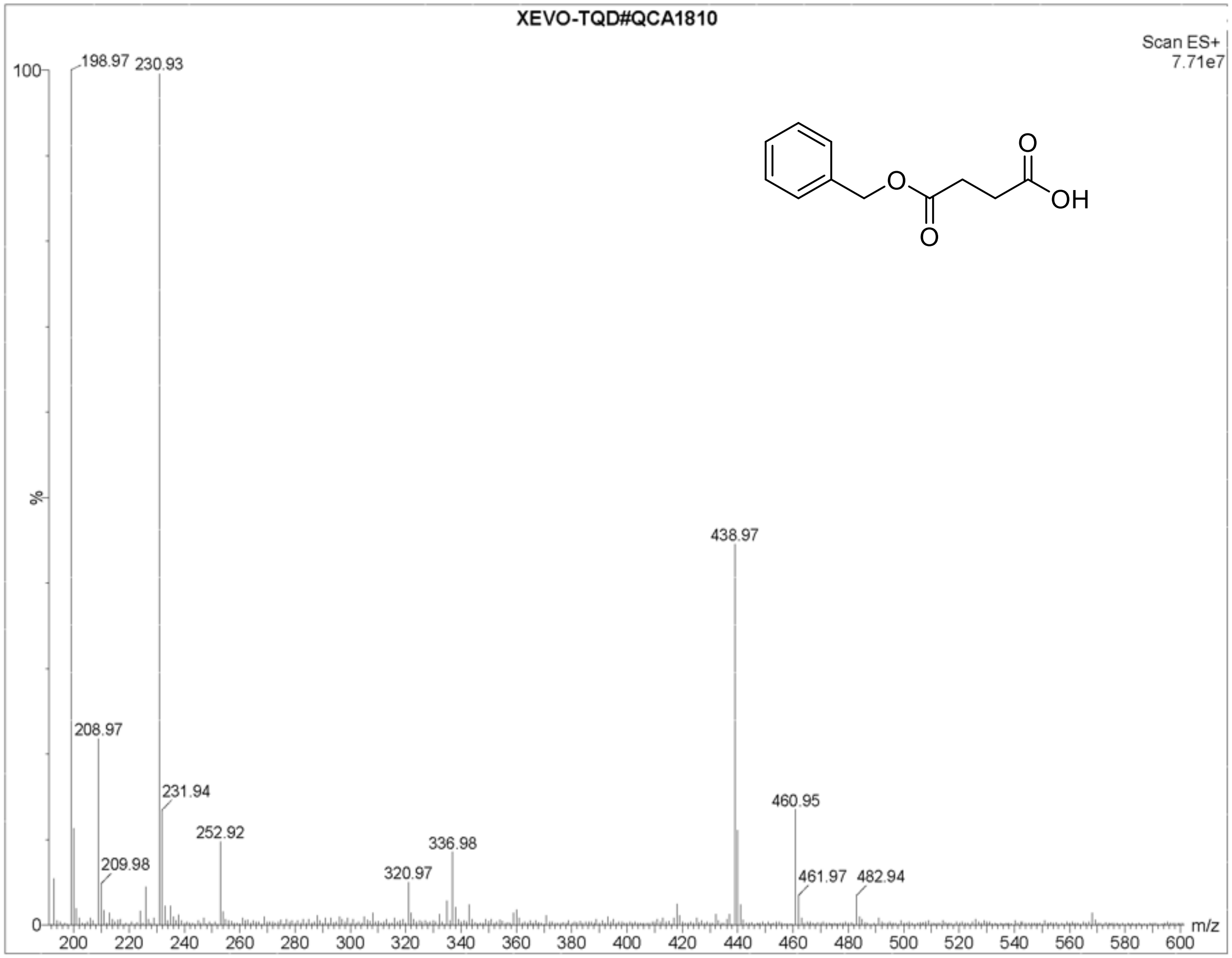

4-(cyclopentyloxy)-4-oxobutanoic acid (S10)

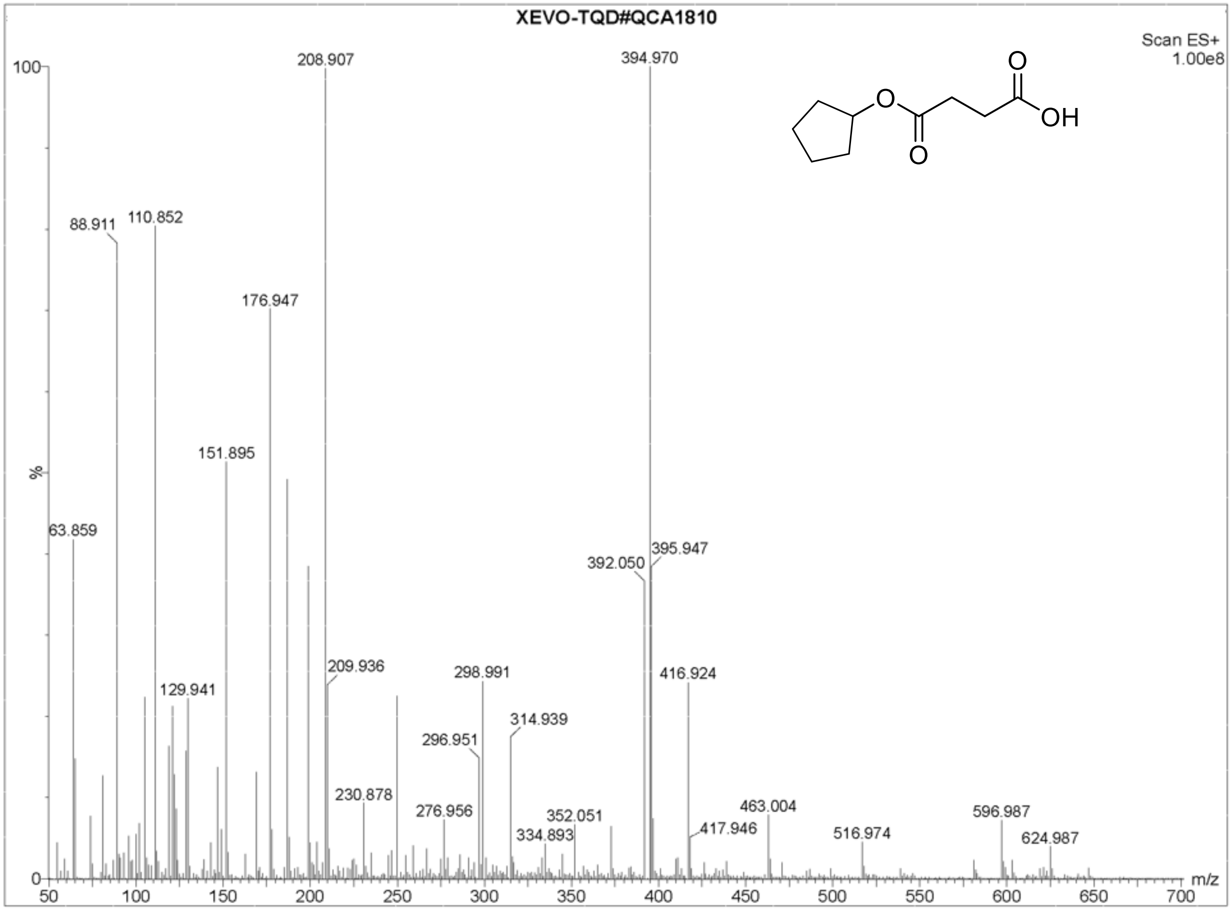

4-(allyloxy)-4-oxobutanoic acid (S11)

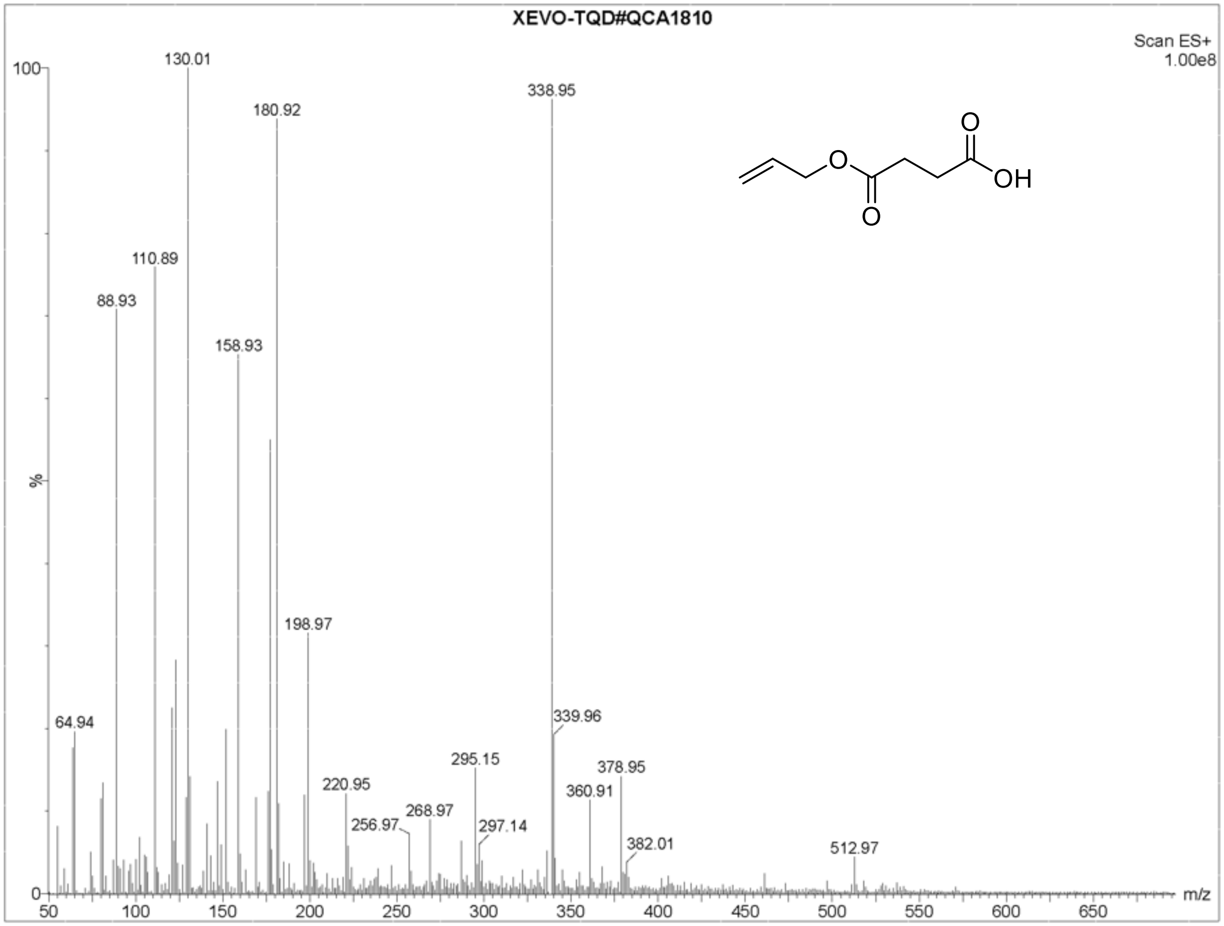

(Z)-O,O'-((3-hydroxy-5-oxohept-3-ene-1,7-diyl)bis(2-methoxy-4,1-phenylene)) di(prop-2-yn-1-yl) disuccinate (3)

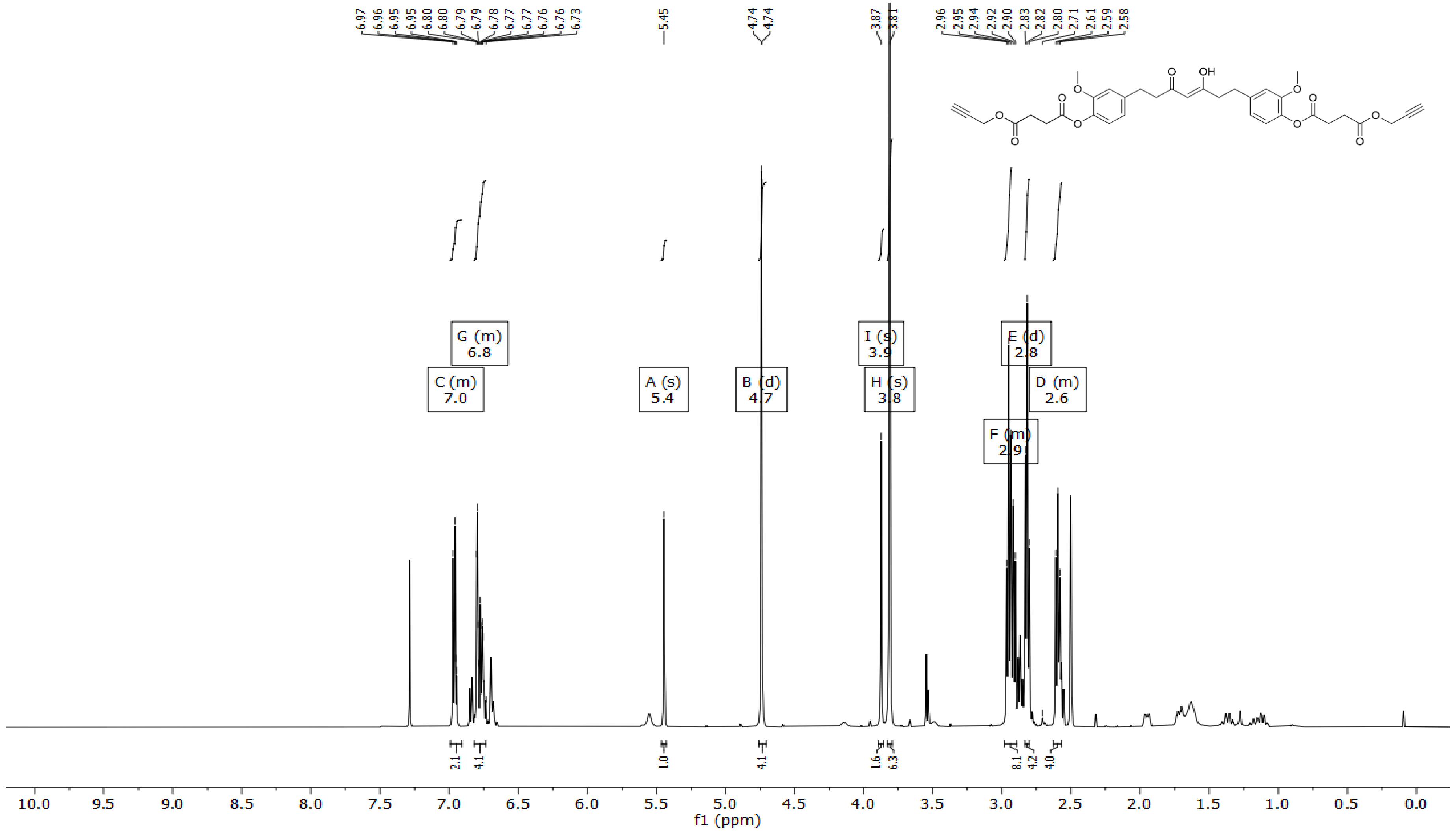

(Z)-O,O'-((3-hydroxy-5-oxohept-3-ene-1,7-diyl)bis(2-methoxy-4,1-phenylene)) di(prop-2-yn-1-yl) disuccinate (3)

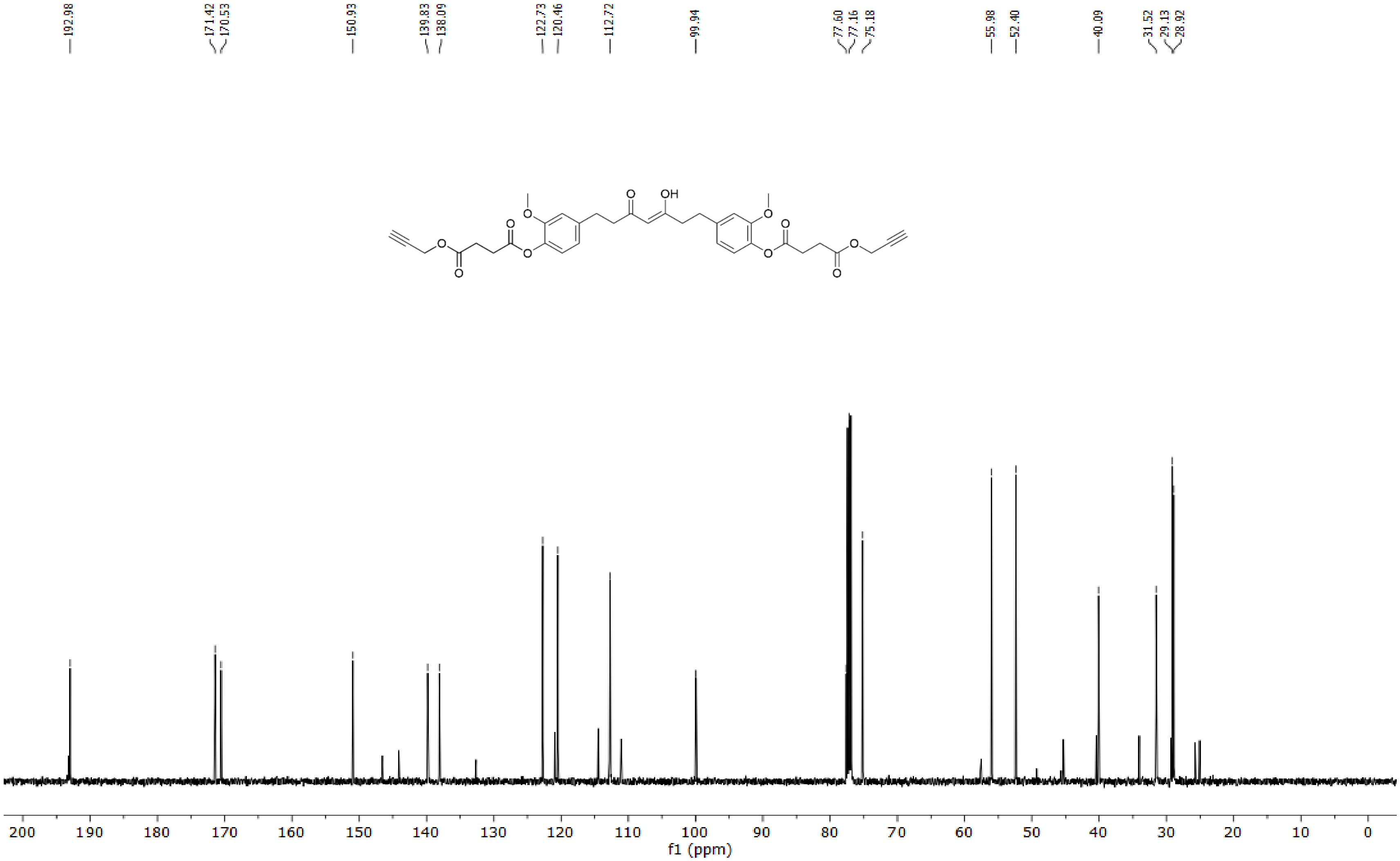

(Z)-di(9H-fluoren-9-yl) O,O'-((3-hydroxy-5-oxohept-3-ene-1,7-diyl)bis(2-methoxy-4,1-phenylene)) disuccinate (4)

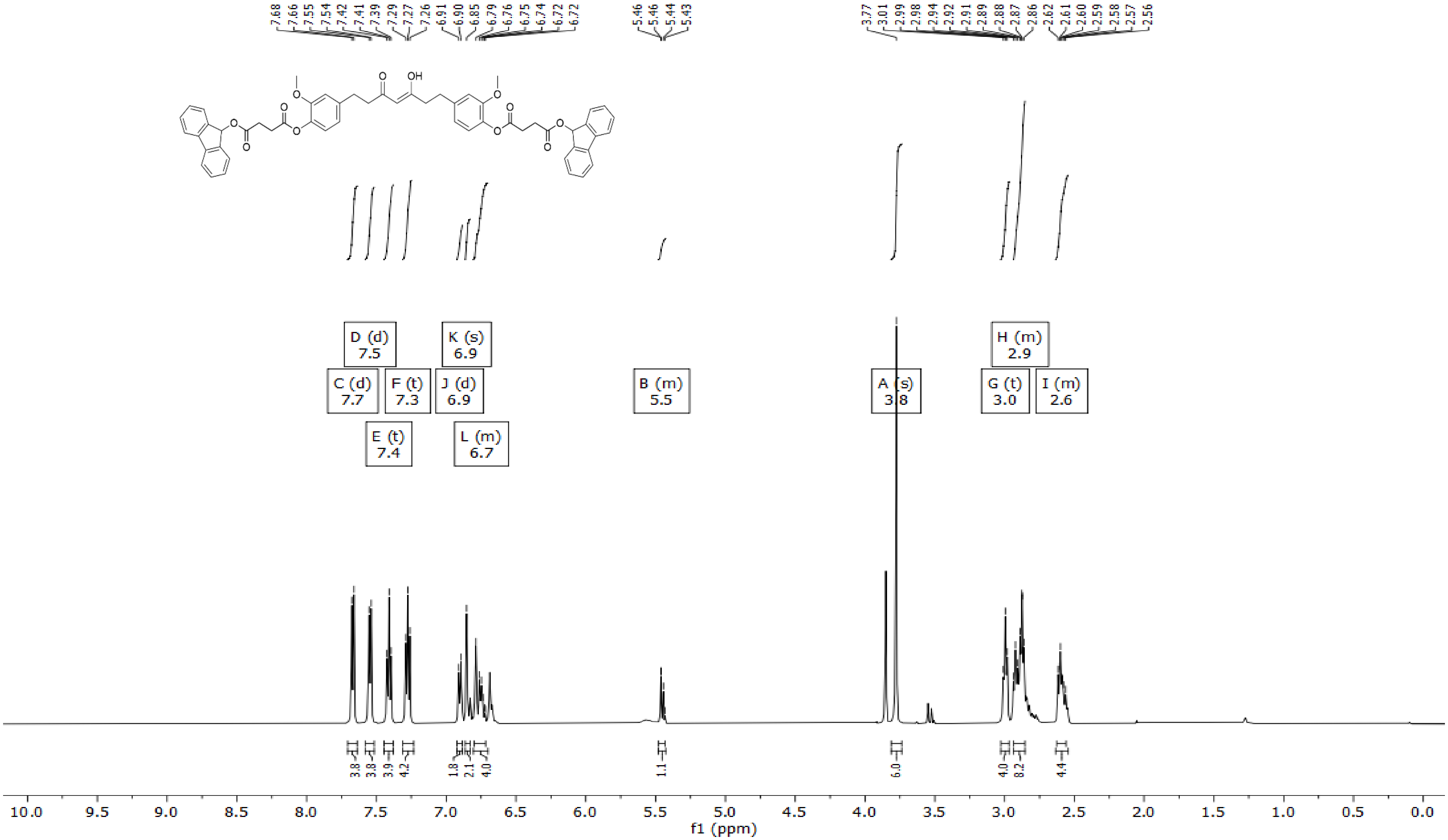

(Z)-di(9H-fluoren-9-yl) O,O'-((3-hydroxy-5-oxohept-3-ene-1,7-diyl)bis(2-methoxy-4,1-phenylene)) disuccinate (4).

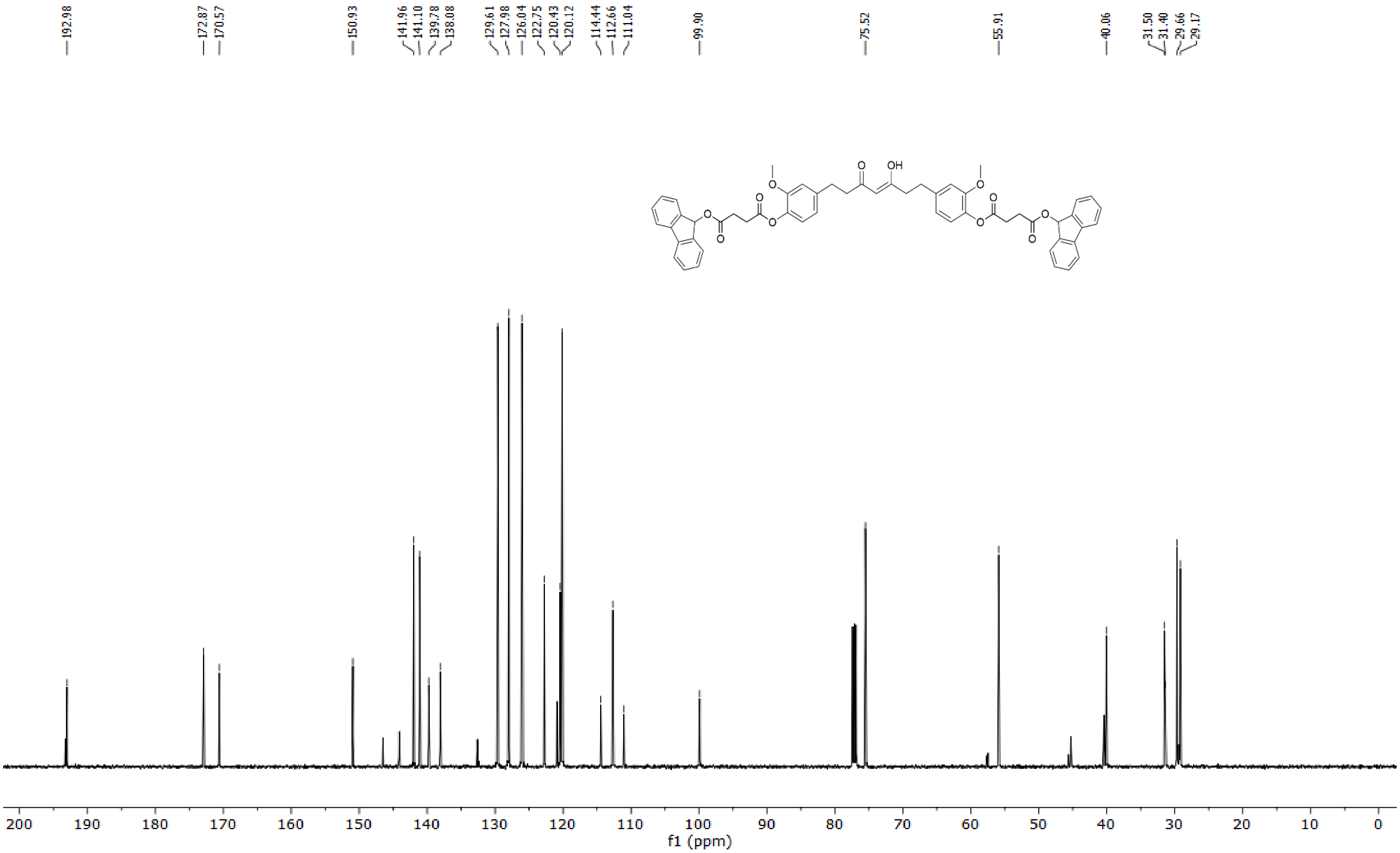

(Z)-bis(2,3-dihydro-1H-inden-2-yl) O,O'-((3-hydroxy-5-oxohept-3-ene-1,7-diyl)bis(2-methoxy-4,1-phenylene)) disuccinate (5)

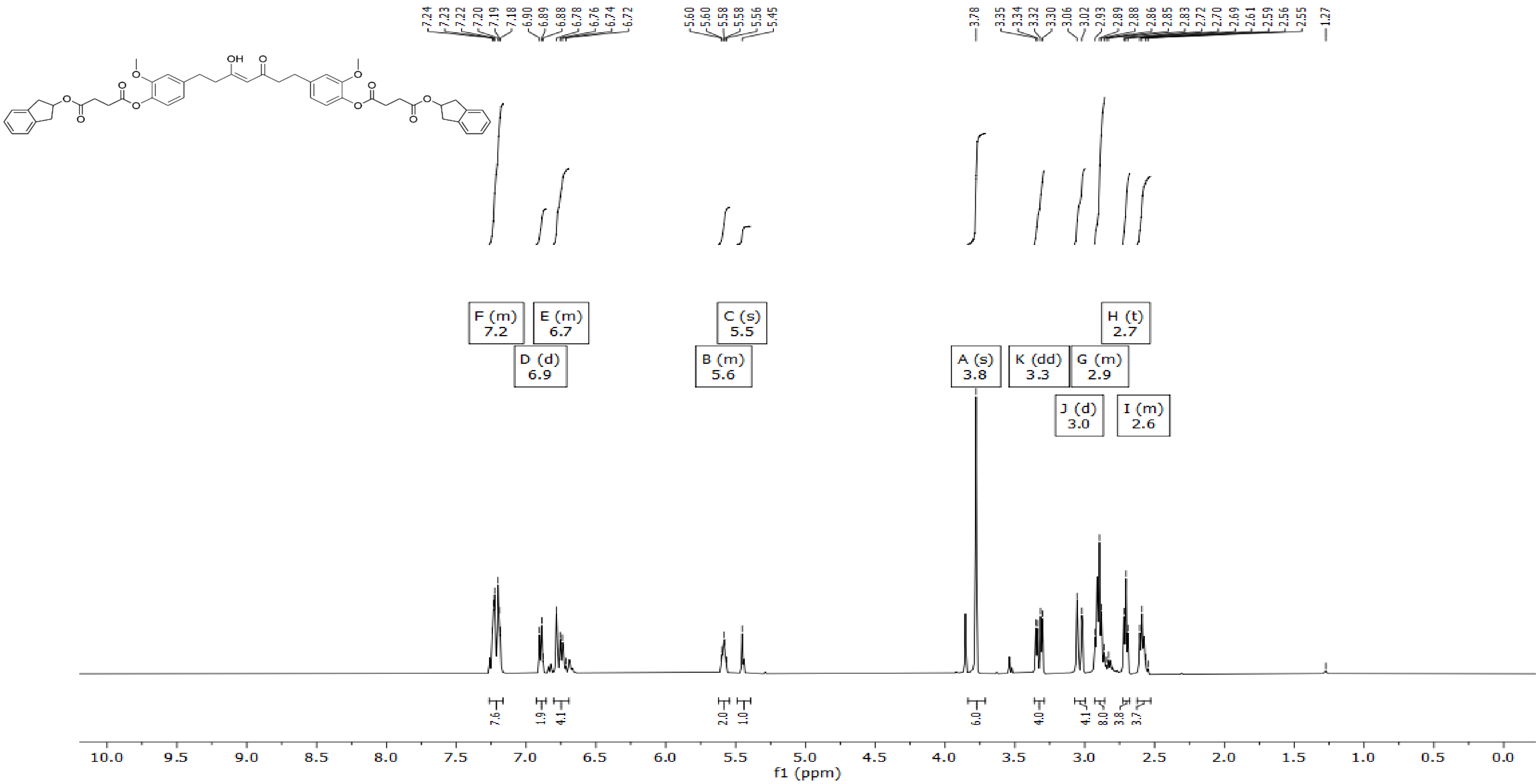

(Z)-bis(2,3-dihydro-1H-inden-2-yl) O,O'-((3-hydroxy-5-oxohept-3-ene-1,7-diyl)bis(2-methoxy-4,1-phenylene)) disuccinate (5)

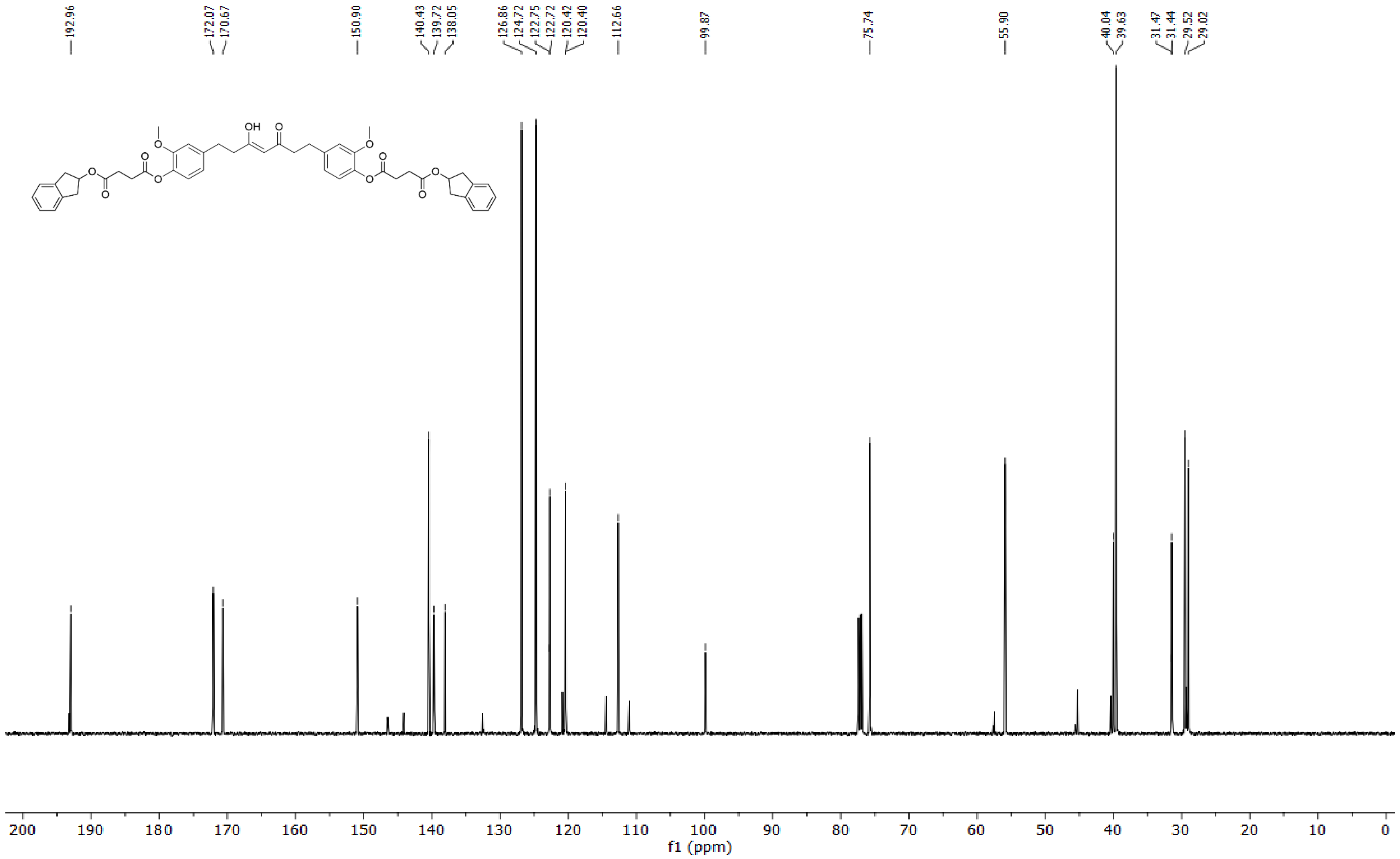

(Z)-dibenzhydryl O,O'-((3-hydroxy-5-oxohept-3-ene-1,7-diyl)bis(2-methoxy-4,1-phenylene)) disuccinate (6)

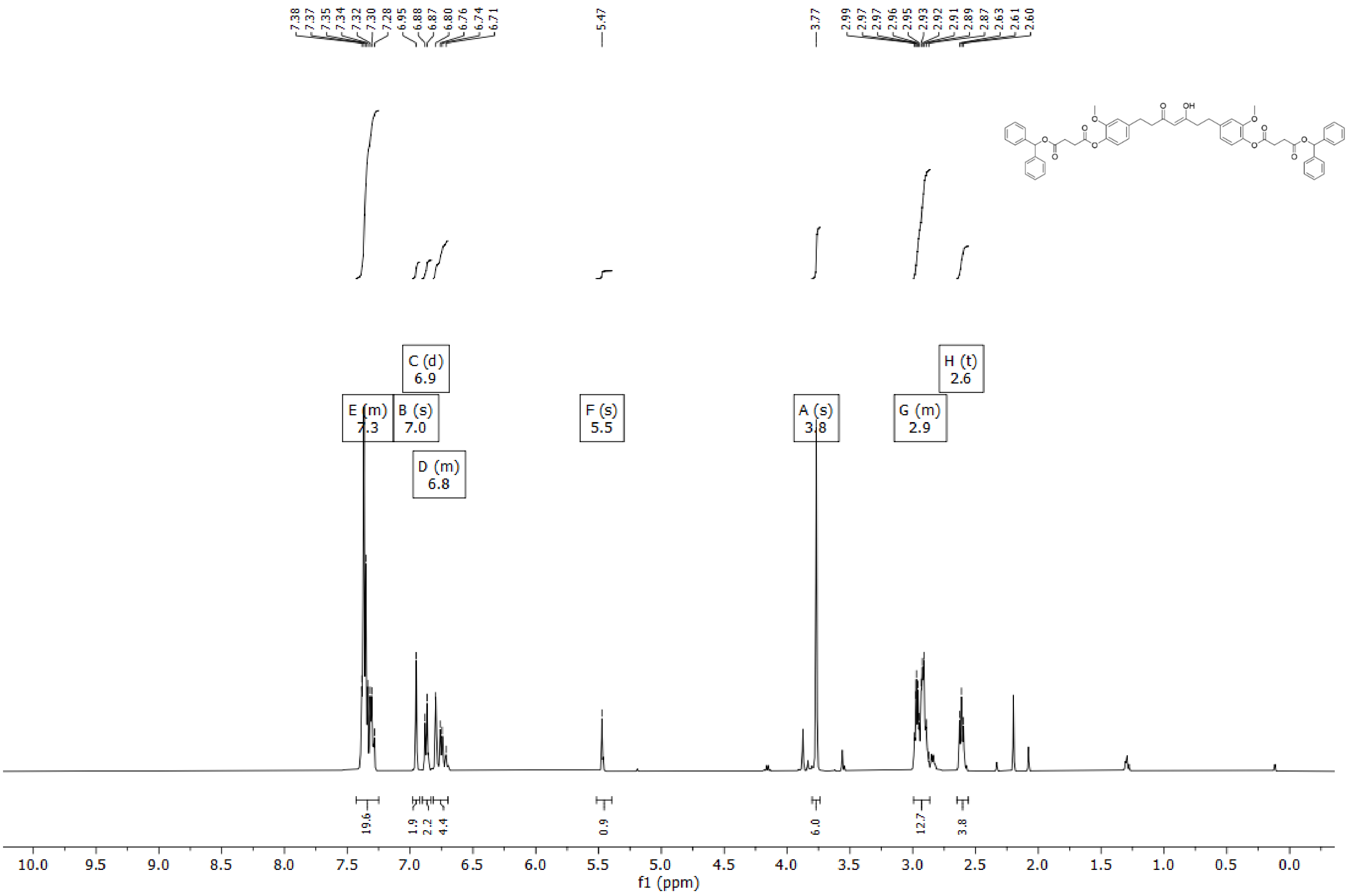

(Z)-dibenzhydryl O,O'-((3-hydroxy-5-oxohept-3-ene-1,7-diyl)bis(2-methoxy-4,1-phenylene)) disuccinate (6)

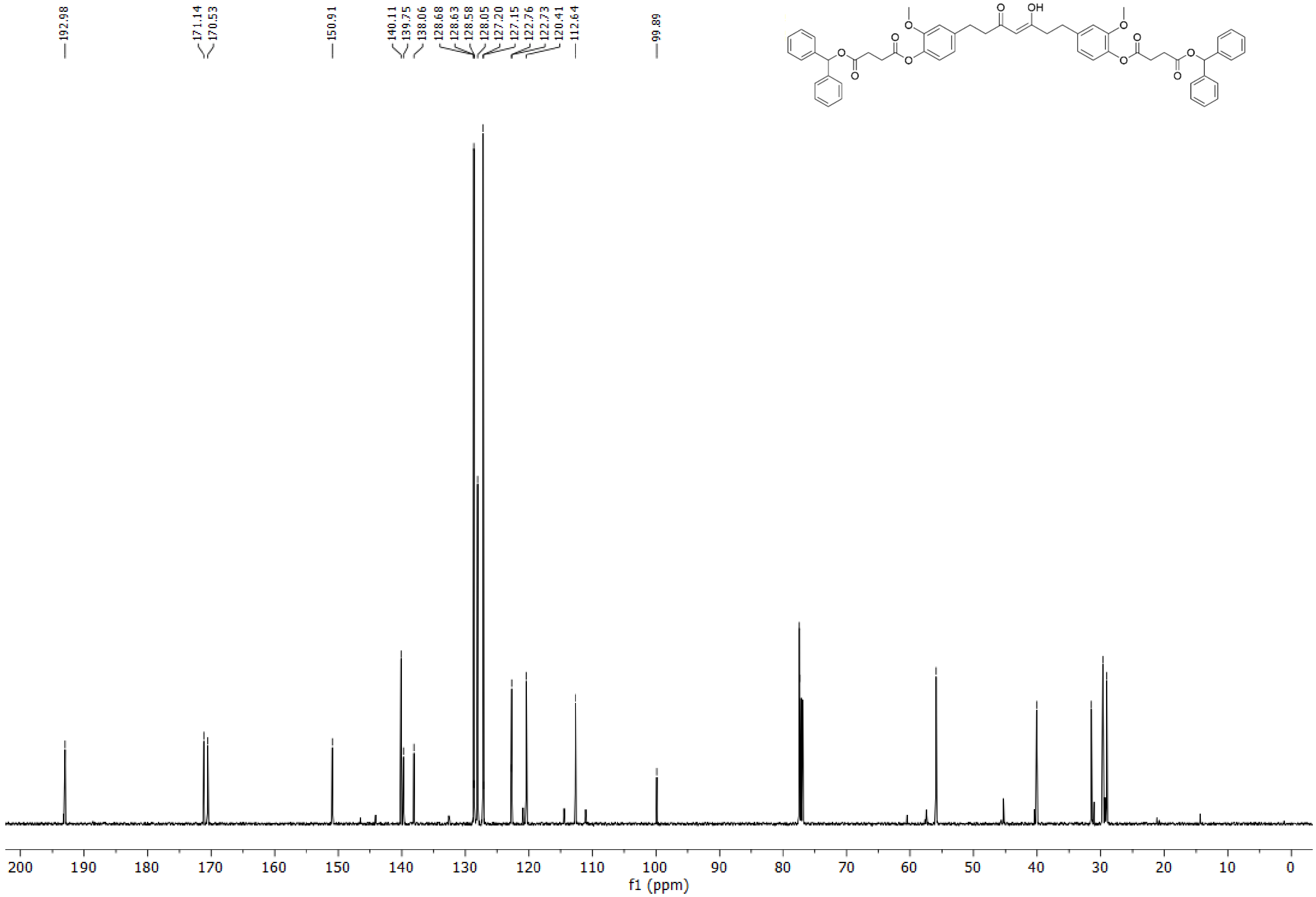

O,O'-(((Z)-3-hydroxy-5-oxohept-3-ene-1,7-diyl)bis(2-methoxy-4,1-phenylene)) bis((1R,2S,5R)-2-isopropyl-5-methylcyclohexyl) disuccinate (7)

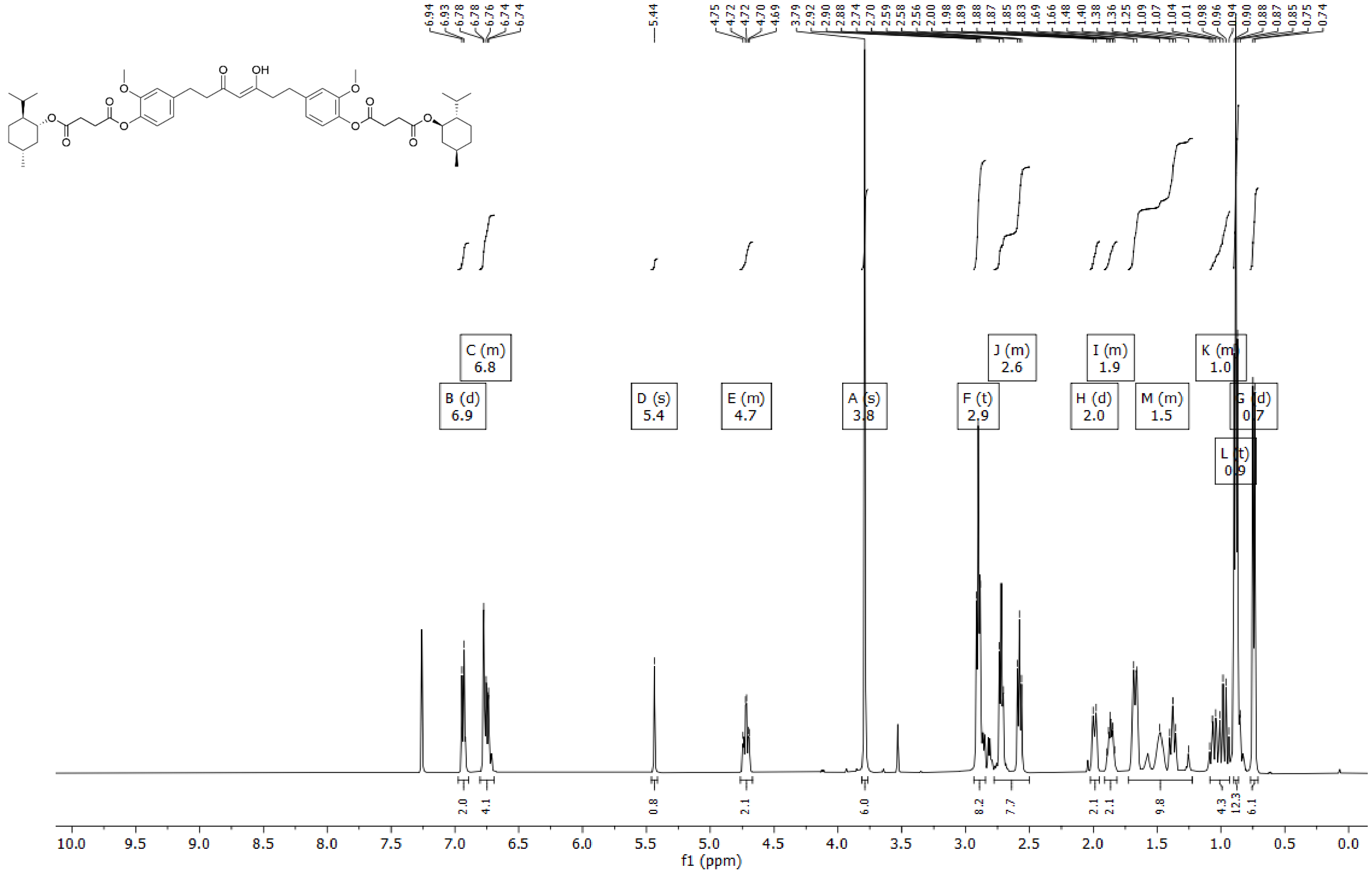

O,O'-(((Z)-3-hydroxy-5-oxohept-3-ene-1,7-diyl)bis(2-methoxy-4,1-phenylene)) bis((1R,2S,5R)-2-isopropyl-5-methylcyclohexyl) disuccinate (7)

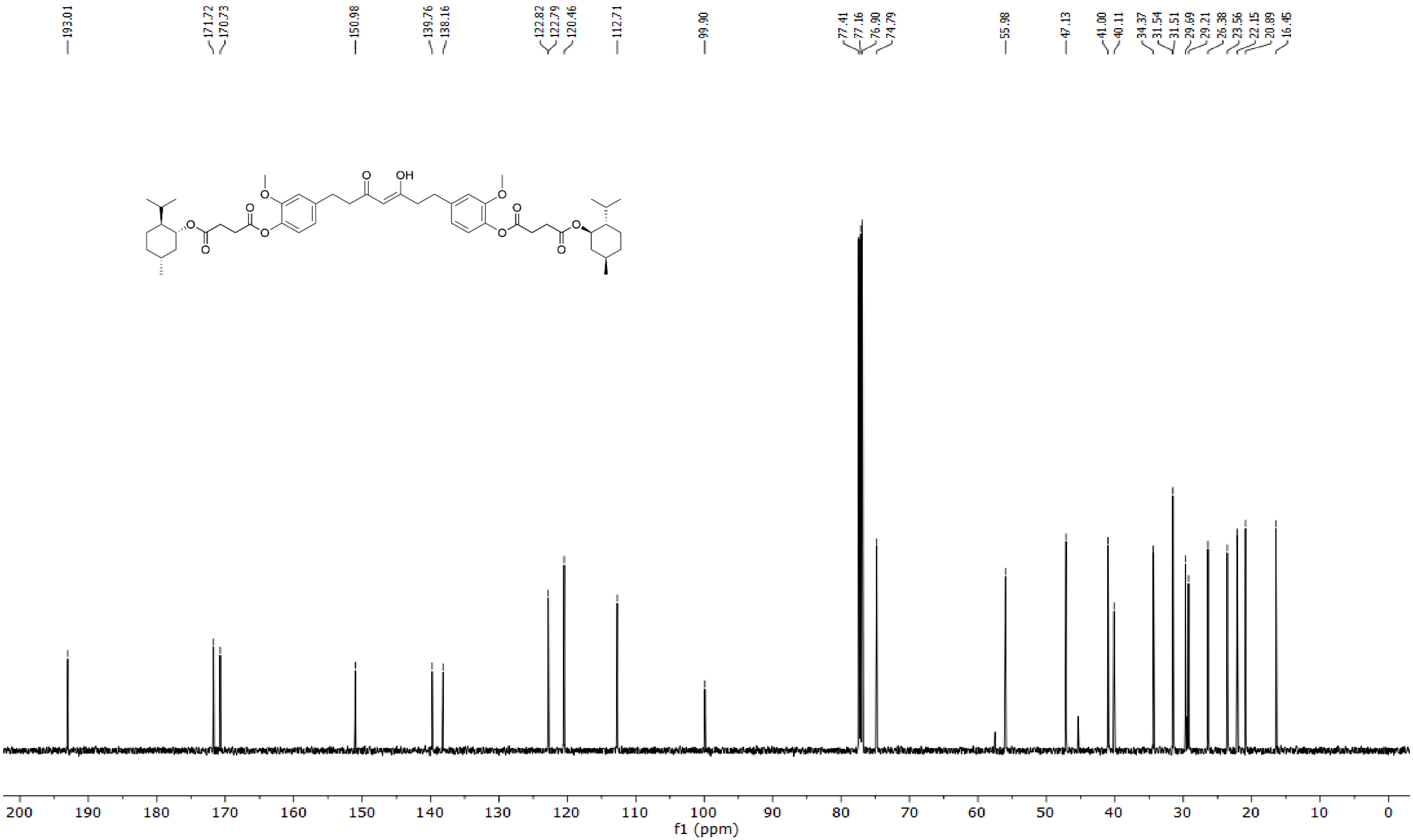

(Z)-O,O'-((3-hydroxy-5-oxohept-3-ene-1,7-diyl)bis(2-methoxy-4,1-phenylene)) bis(1,2,3,4-tetrahydronaphthalen-1-yl) disuccinate (8)

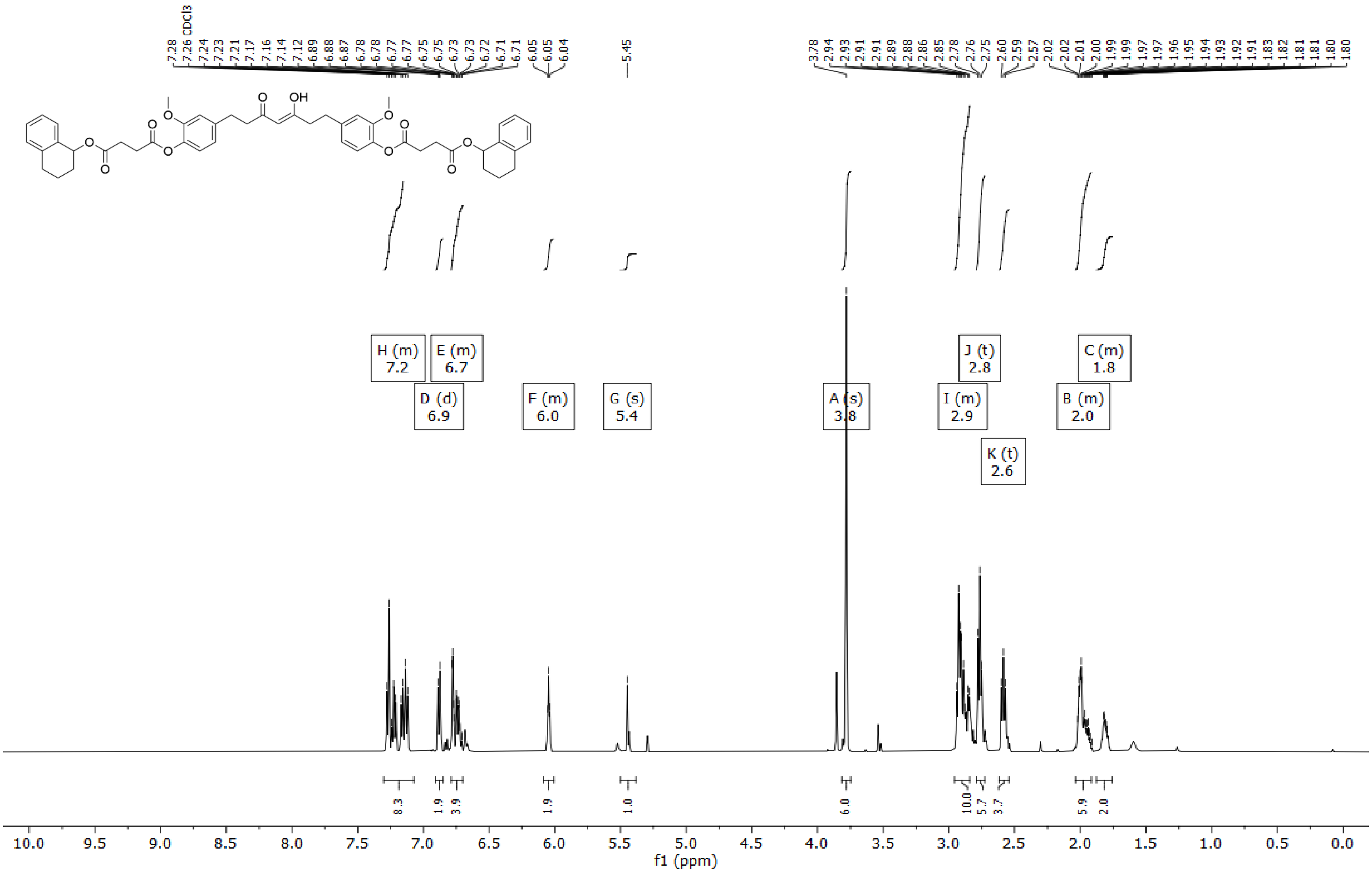

(Z)-O,O'-((3-hydroxy-5-oxohept-3-ene-1,7-diyl)bis(2-methoxy-4,1-phenylene)) bis(1,2,3,4-tetrahydronaphthalen-1-yl) disuccinate (8)

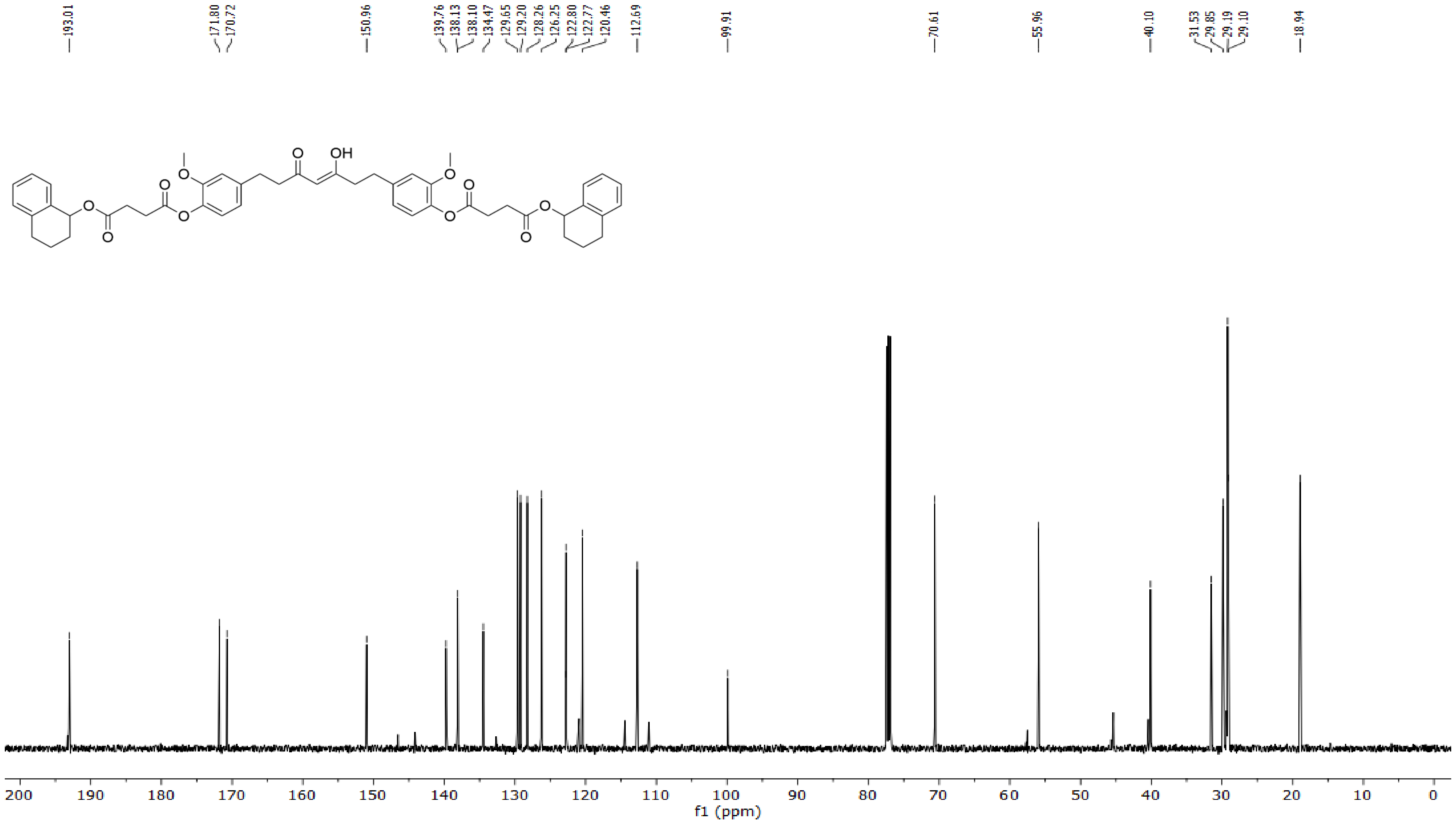

(Z)-dibenzyl O,O'-((3-hydroxy-5-oxohept-3-ene-1,7-diyl)bis(2-methoxy-4,1-phenylene)) disuccinate (9)

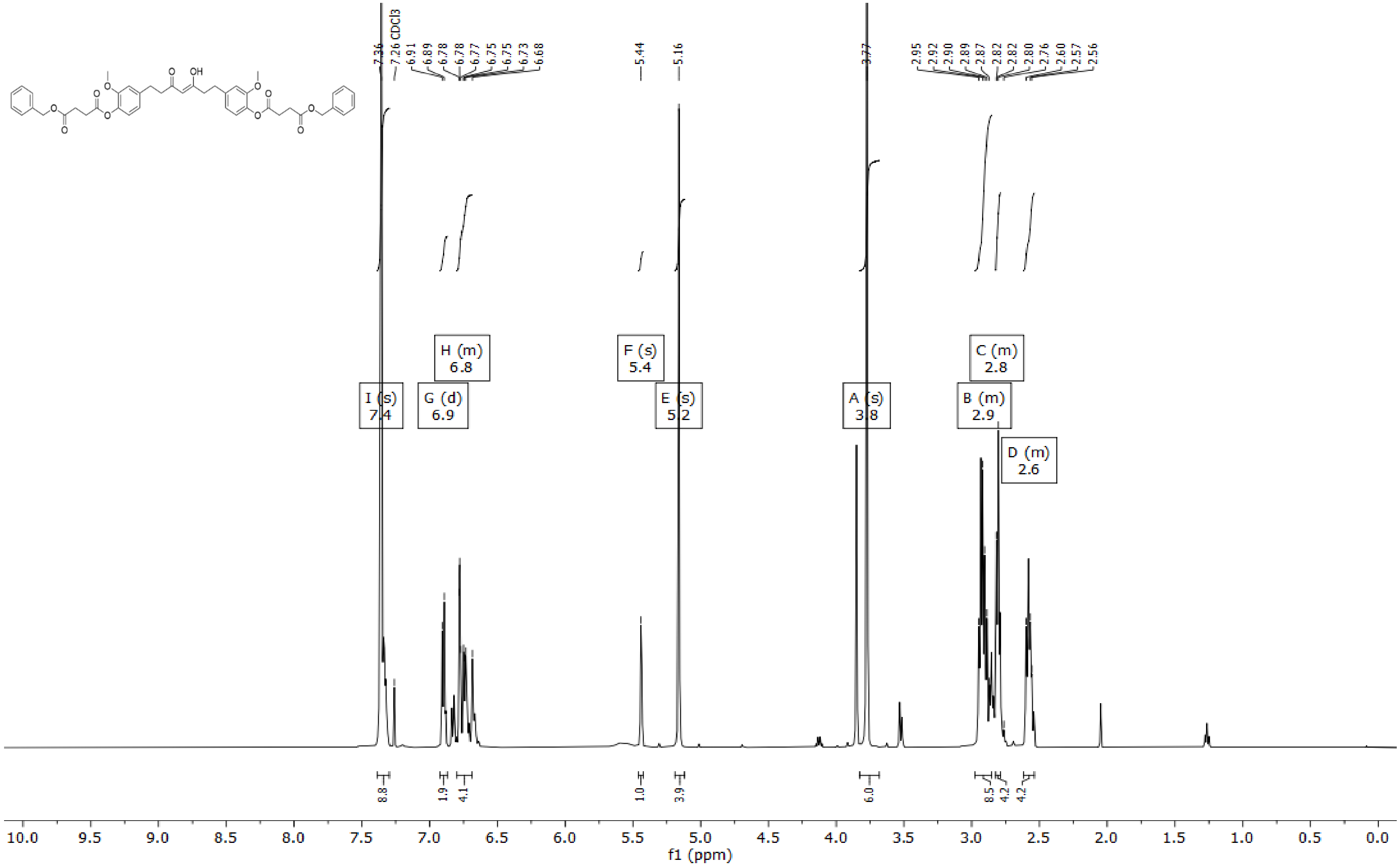

(Z)-dibenzyl O,O'-((3-hydroxy-5-oxohept-3-ene-1,7-diyl)bis(2-methoxy-4,1-phenylene)) disuccinate (9)

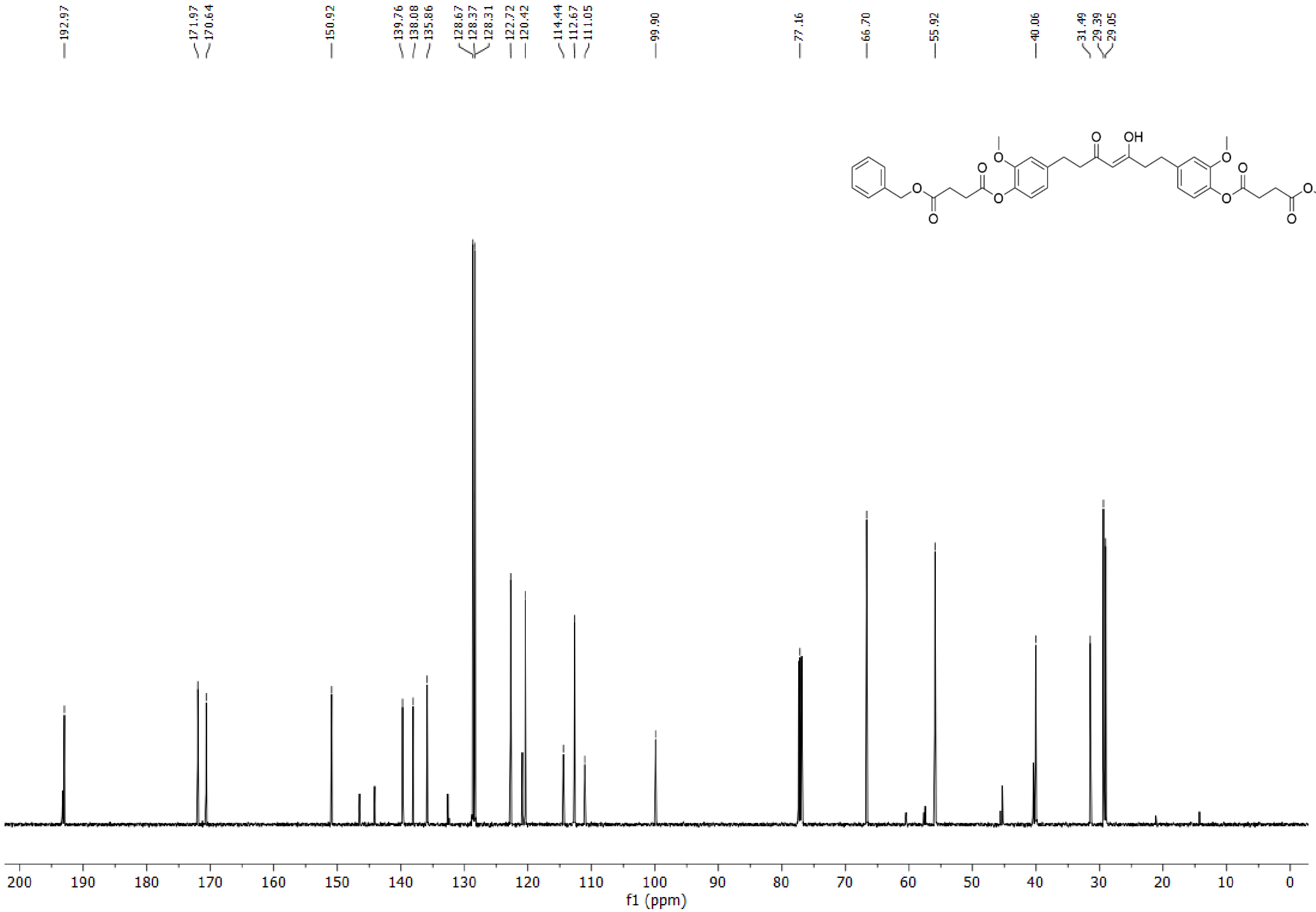

(Z)-dicyclopentyl O,O'-((3-hydroxy-5-oxohept-3-ene-1,7-diyl)bis(2-methoxy-4,1-phenylene)) disuccinate (10)

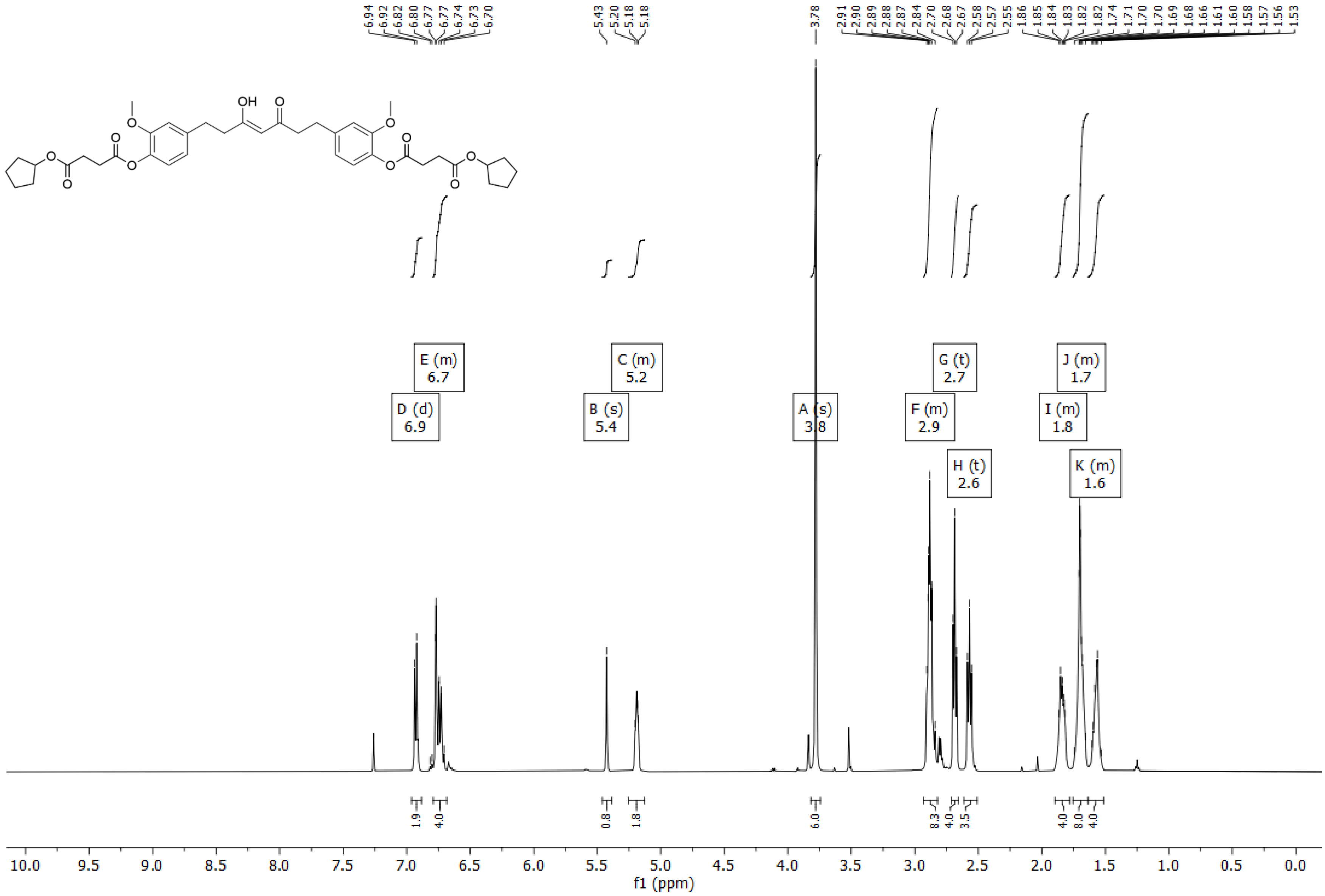

(Z)-dicyclopentyl O,O'-((3-hydroxy-5-oxohept-3-ene-1,7-diyl)bis(2-methoxy-4,1-phenylene)) disuccinate (10)

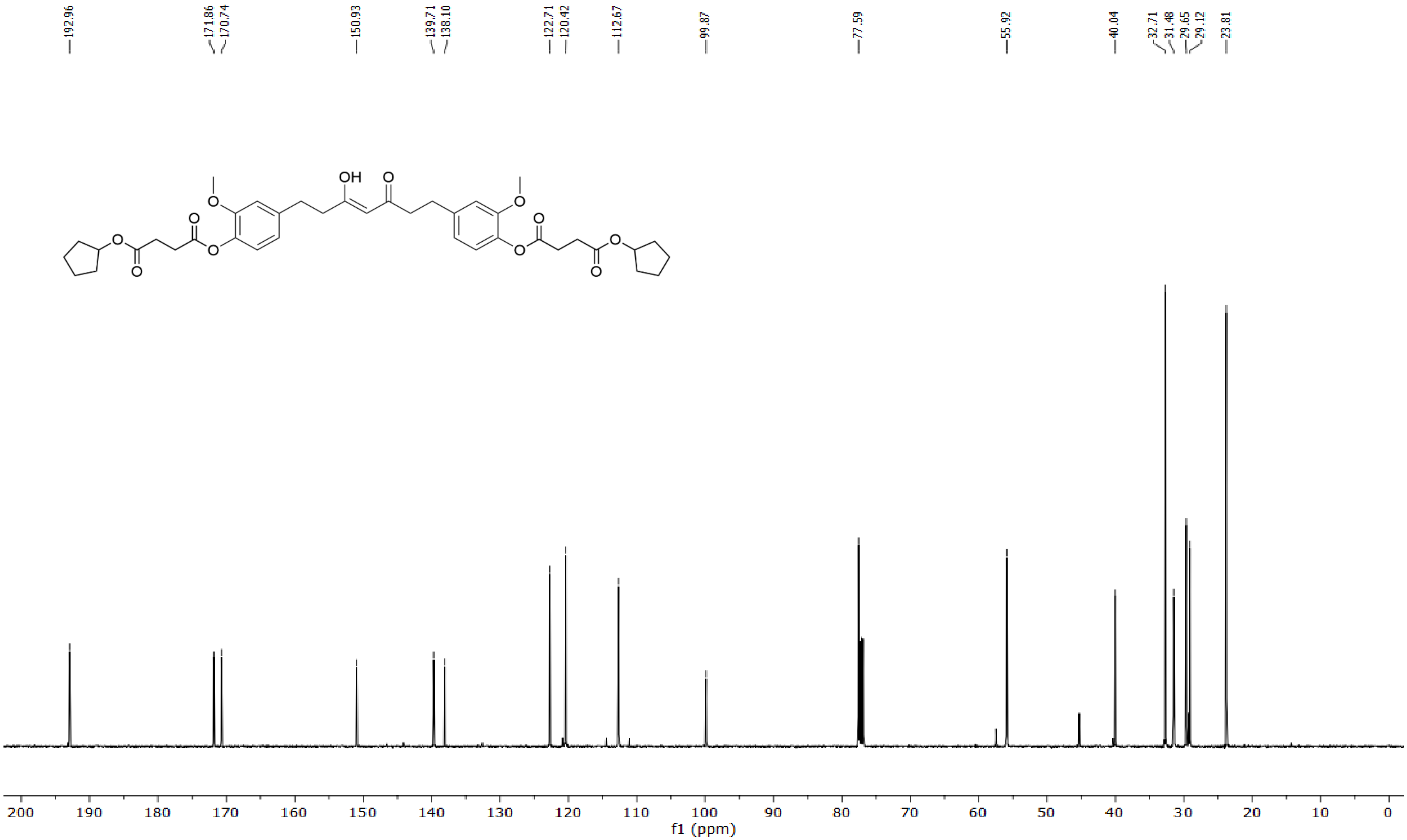

(Z)-O,O'-((3-hydroxy-5-oxohept-3-ene-1,7-diyl)bis(2-methoxy-4,1-phenylene)) dimethyl disuccinate (11)

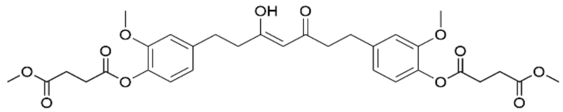

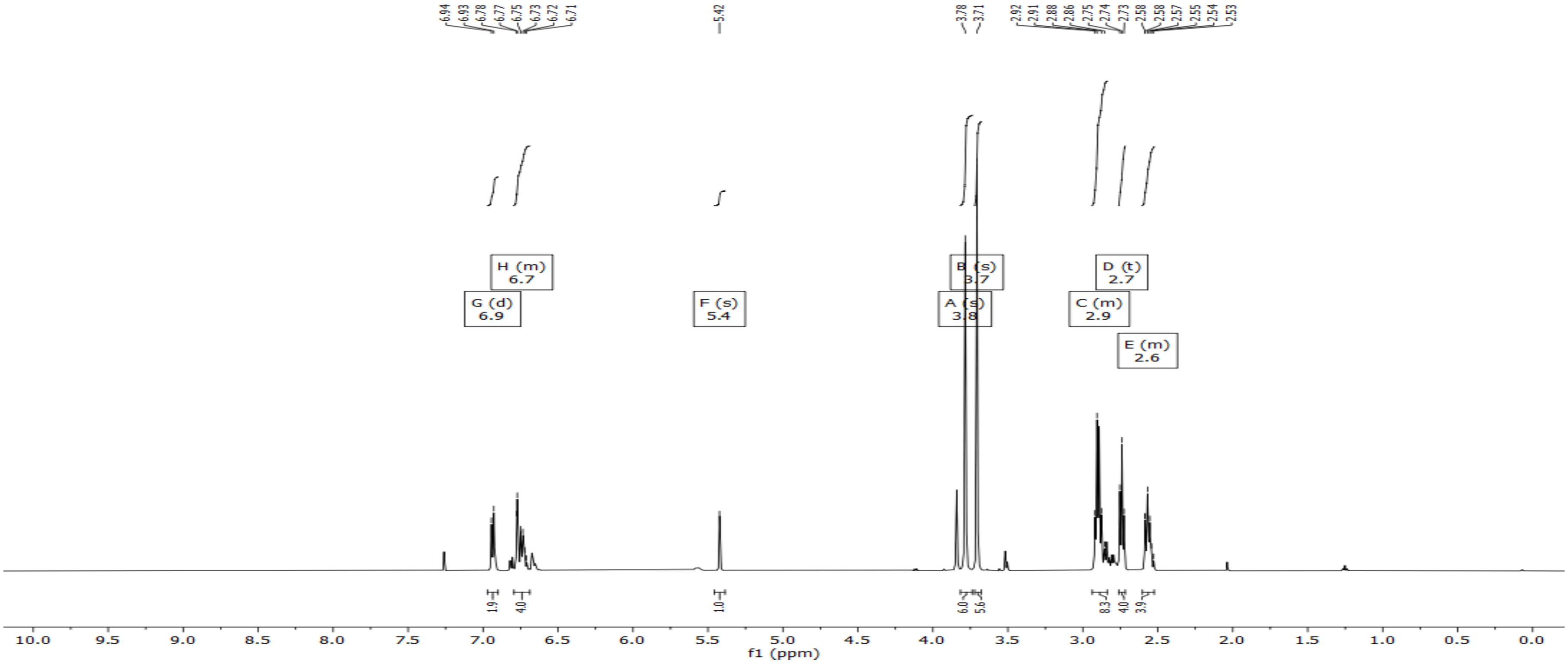

(Z)-O,O'-((3-hydroxy-5-oxohept-3-ene-1,7-diyl)bis(2-methoxy-4,1-phenylene)) dimethyl disuccinate (11)

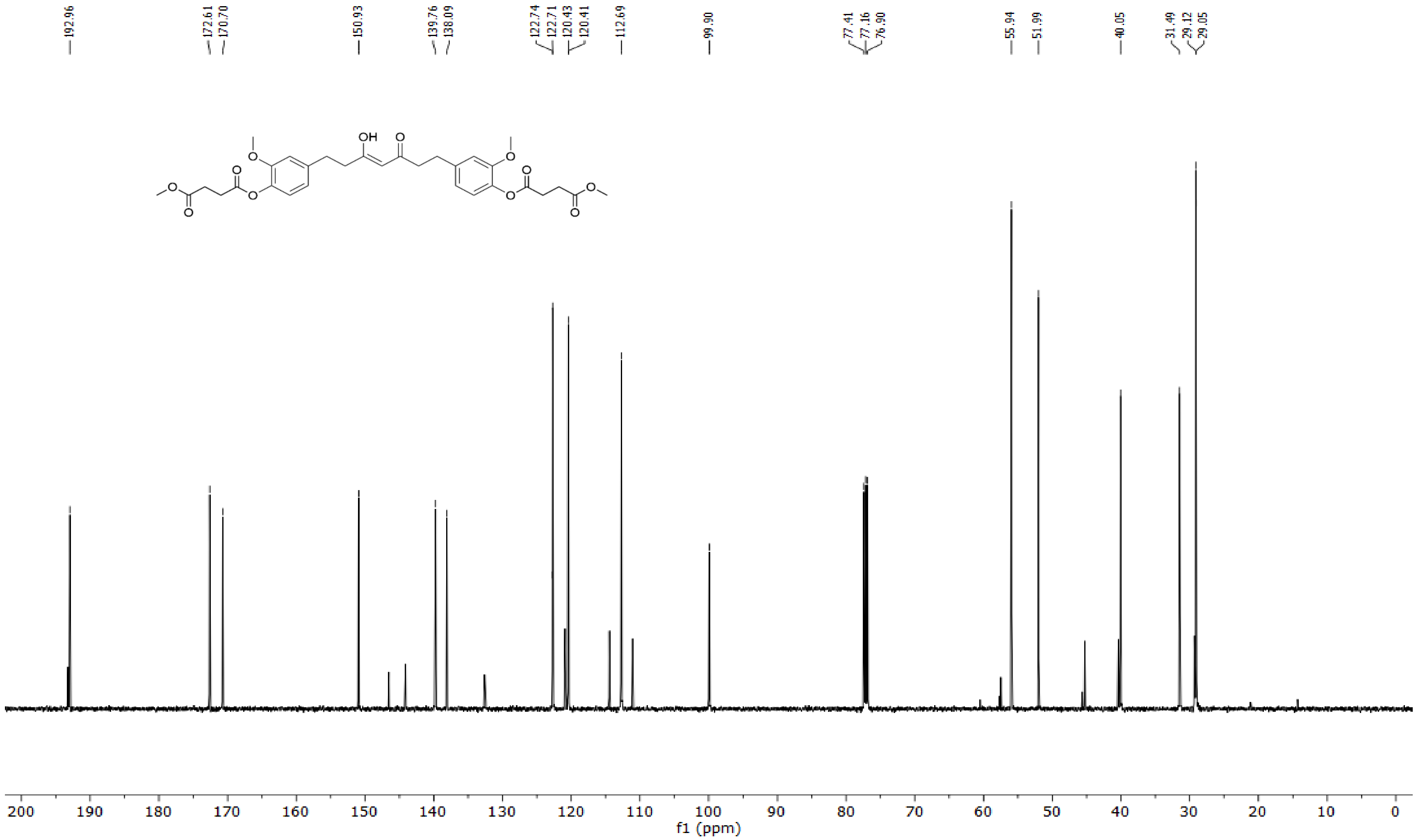

(Z) -diallyl O,O'-((3-hydroxy-5-oxohept-3-ene-1,7-diyl)bis(2-methoxy-4,1-phenylene)) disuccinate (12)

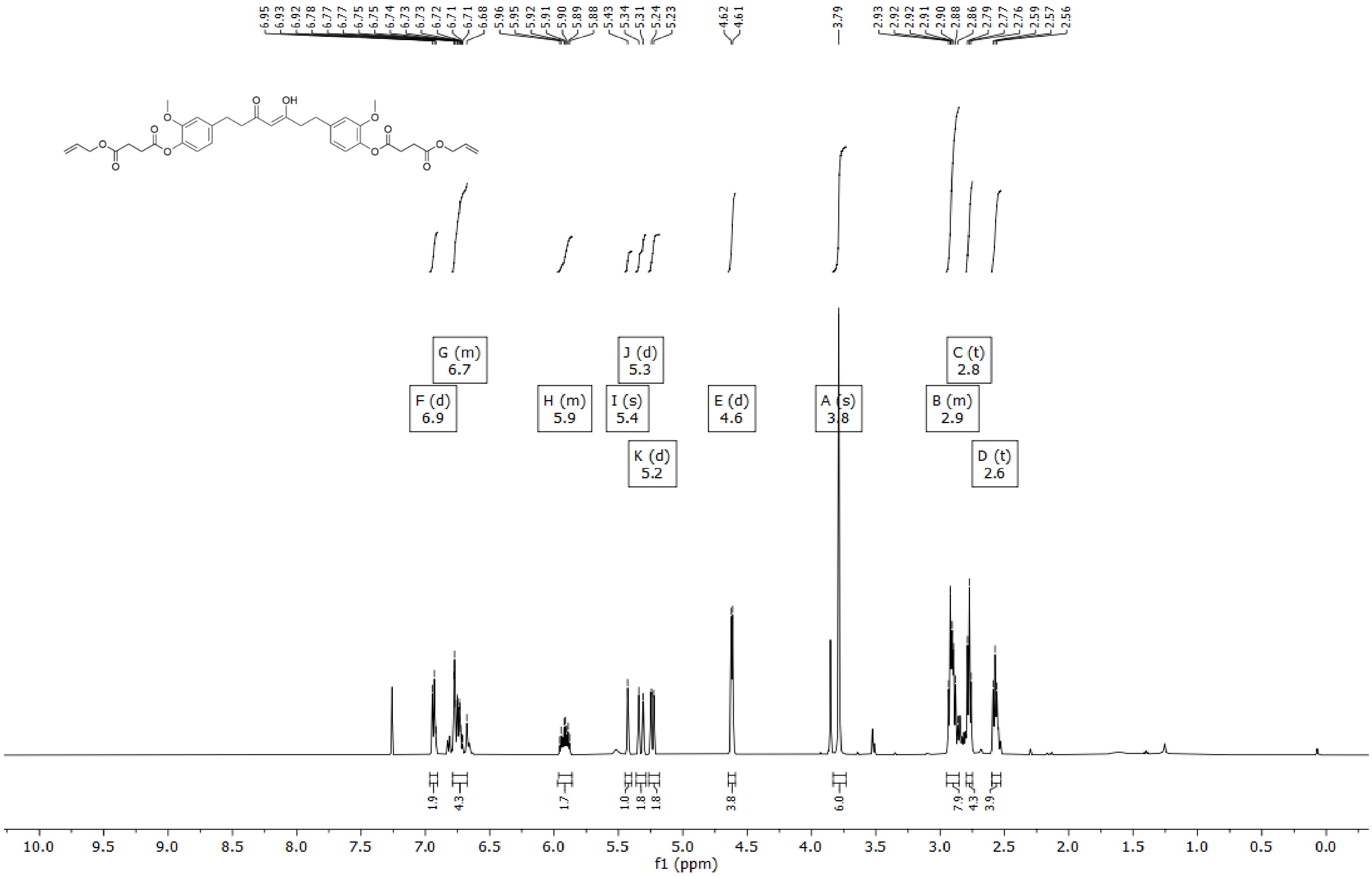

(Z)-diallyl O,O'-((3-hydroxy-5-oxohept-3-ene-1,7-diyl)bis(2-methoxy-4,1-phenylene)) disuccinate (12)

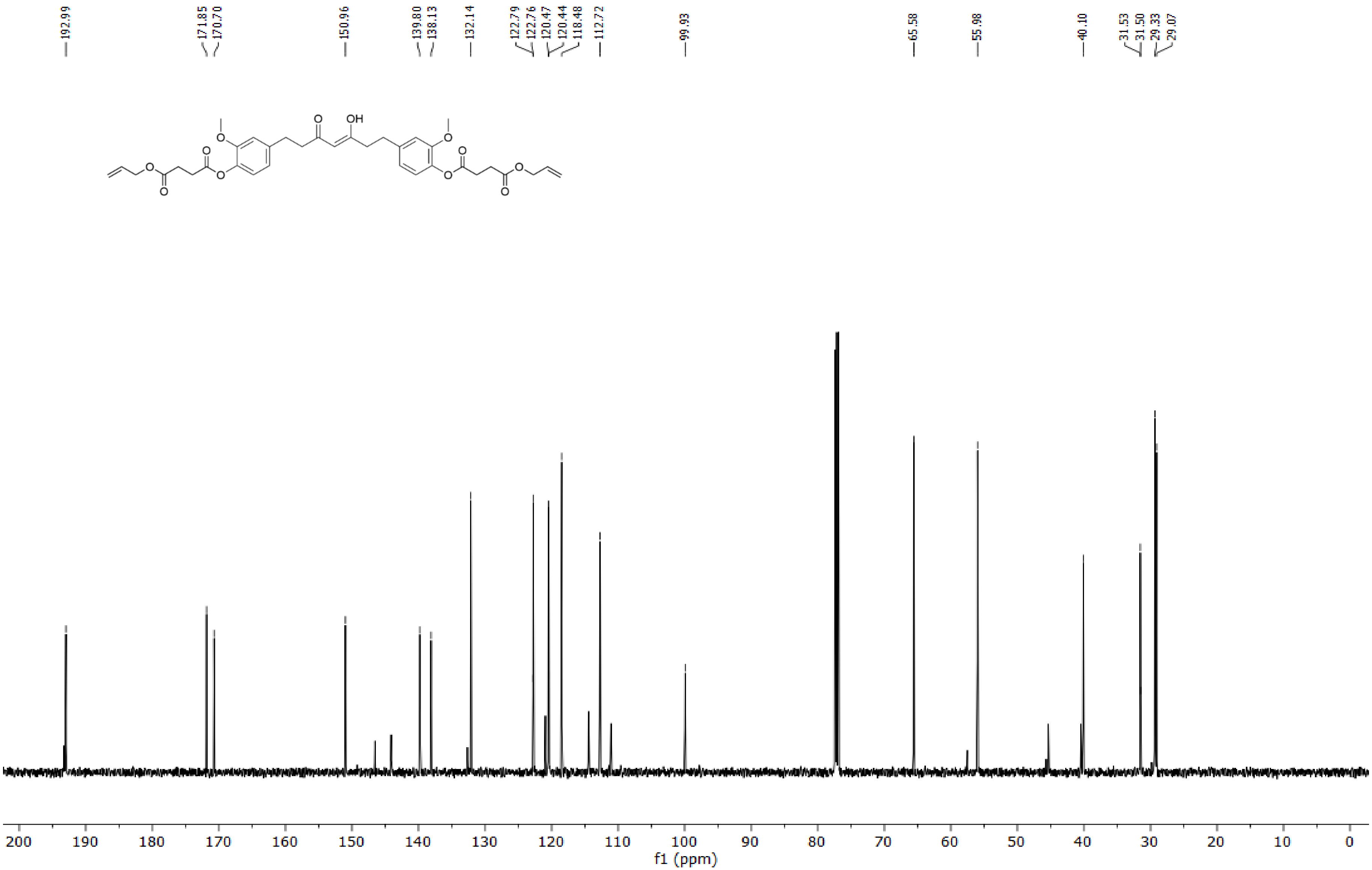

di((1r,3r,5r,7r)-adamantan-2-yl) O,O'-(((Z)-3-hydroxy-5-oxohept-3-ene-1,7-diyl)bis(2-methoxy-4,1-phenylene)) disuccinate (13)

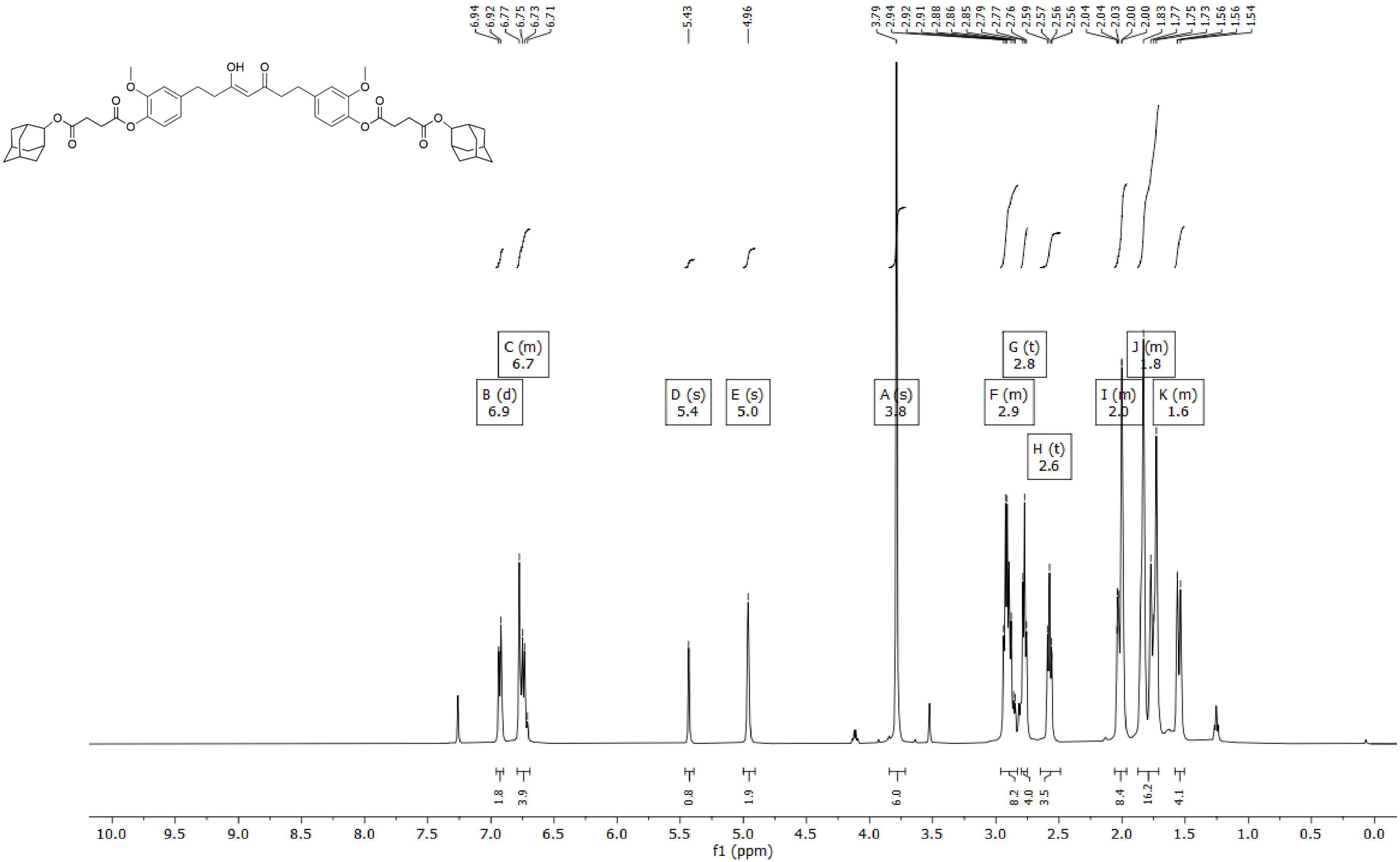

di((1r,3r,5r,7r)-adamantan-2-yl) O,O'-(((Z)-3-hydroxy-5-oxohept-3-ene-1,7-diyl)bis(2-methoxy-4,1-phenylene)) disuccinate (13)

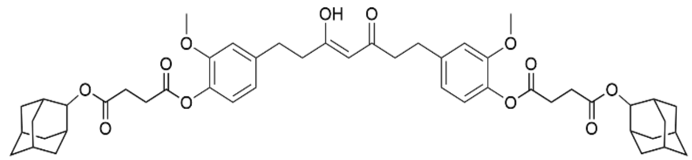

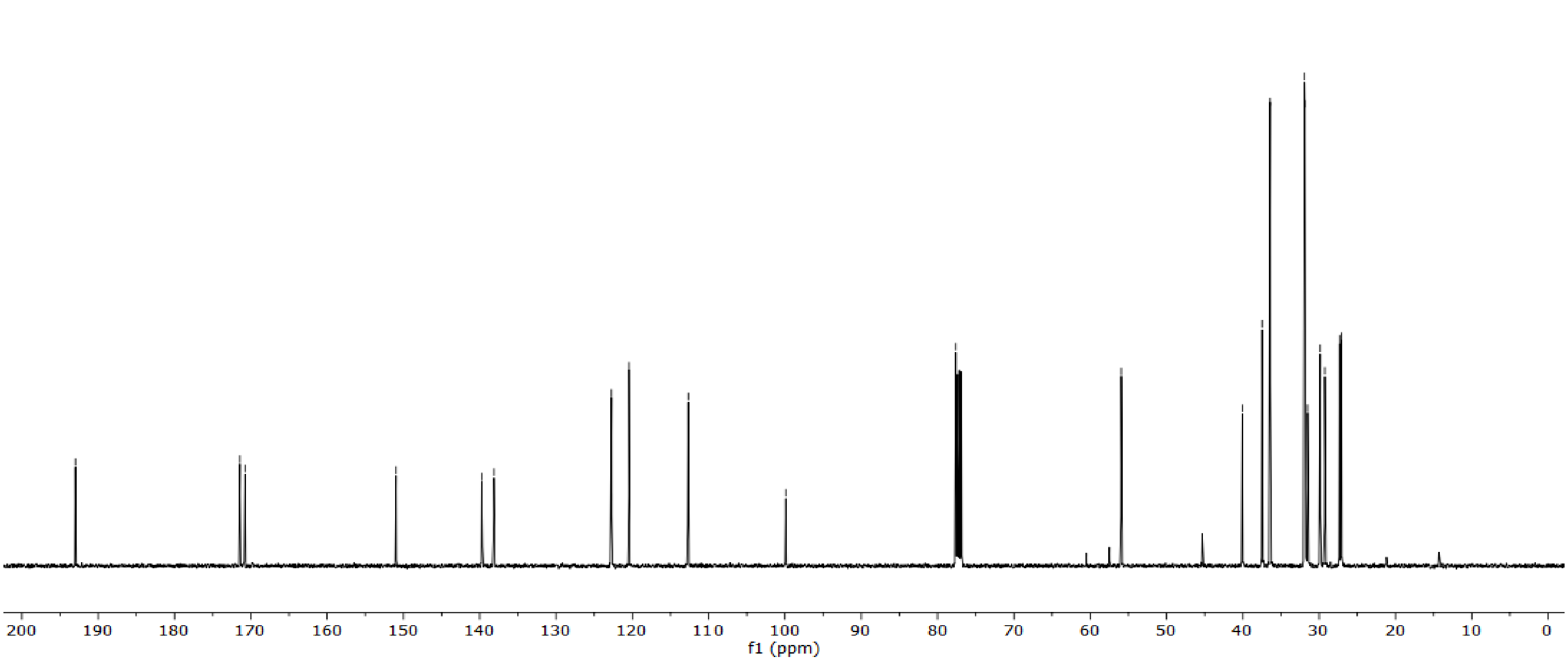

Supplement: Supplementary file 1 [file molecules-28-07787-s001.zip › molecules-2697573-supplementary.pdf]
